# Modulation of steroidogenesis by *Actaea racemosa* and vitamin C combination, in letrozole induced polycystic ovarian syndrome rat model: promising activity without the risk of hepatic adverse effect

**DOI:** 10.1186/s13020-021-00444-z

**Published:** 2021-04-29

**Authors:** Asmaa A. Azouz, Sara E. Ali, Reham M. Abd-Elsalam, Shimaa R. Emam, Mona K. Galal, Sherif H. Elmosalamy, Muhammed A. Alsherbiny, Bardes B. Hassan, Chun Guang Li, Shymaa A. El Badawy

**Affiliations:** 1 Department of Pharmacology, Faculty of Veterinary Medicine, Cairo University, Giza, 12211 Egypt; 2 Department of Physiology, Faculty of Veterinary Medicine, Cairo University, Giza, 12211 Egypt; 3 Department of Pathology, Faculty of Veterinary Medicine, Cairo University, Giza, 12211 Egypt; 4 Department of Biochemistry, Faculty of Veterinary Medicine, Cairo University, Giza, 12211 Egypt; 5 Department of Pharmacognosy, Faculty of Pharmacy, Cairo University, Cairo, 12613 Egypt; 6 NICM Health Research Institute, Western Sydney University, Westmead, NSW 2145 Australia

**Keywords:** *Actaea racemosa*, Vitamin C, Polycystic ovarian syndrome, Hepatotoxicity, PCOS rat model, UPLC-QTOF-MS, Metabolomic profiling

## Abstract

**Background:**

Complementary remedies such as the Chinese herb ‘Sheng Ma’ (Black cohosh; *Actaea racemosa* ‘AR’) are being sought to overcome the shortcomings of conventional hormonal and surgical therapies developed for the treatment of polycystic ovary syndrome (PCOS). However, AR-induced hepatotoxicity necessitates a cautionary warning to be labeled on its products as recommended by the United States Pharmacopeia, where four out of seven hepatotoxic cases in Sweden were possibly associated with black cohosh products.

**Methods:**

We investigated the effects, safety, and molecular targets of black cohosh ethanolic extract and/or vitamin C on ovarian functionality and oxidative response in hyperandrogenism-induced PCOS rats. A well-established rat model using oral letrozole, daily, for 21 days was employed. The rats then received the AR extract with and without vitamin C for 28 days. The hormonal evaluation, antioxidant status, histopathological examination, immunohistochemical analysis, cell proliferation, and the expression ratio of the aromatase (Cyp19α1) gene were evaluated. Additionally, holistic profiling of the AR arsenal of secondary metabolites was performed using ultra-high-performance liquid chromatography (UHPLC) coupled with quadrupole high-resolution time of flight mass spectrometry (QTOF-MS).

**Results:**

Beneficial effects were exerted by AR in PCOS rats as antioxidant status, hormonal profile, lipid profile, glucose level, liver functions, and the induced Ki-67 expression in the granulosa, theca cell layers and interstitial stromal cells were all improved. Notably, the combination of AR with vitamin C was not only more effective in reversing the dysregulated levels of testosterone, luteinizing hormone, and mRNA level of Cyp19α1 gene in the PCOS rat, but also safer. The combination regulated both ovarian and hepatic malondialdehyde (MDA) and glutathione (GSH) levels with histological improvement observed in the liver and ovaries. In addition, the untargeted metabolomic profiling enabled the identification of 61 metabolites allocated in five major chemical classes.

**Conclusion:**

This study demonstrated the benefit of the combinatorial effects of AR and vitamin C in mitigating the reproductive and metabolic disorders associated with PCOS with the elimination of AR hepatotoxic risk.

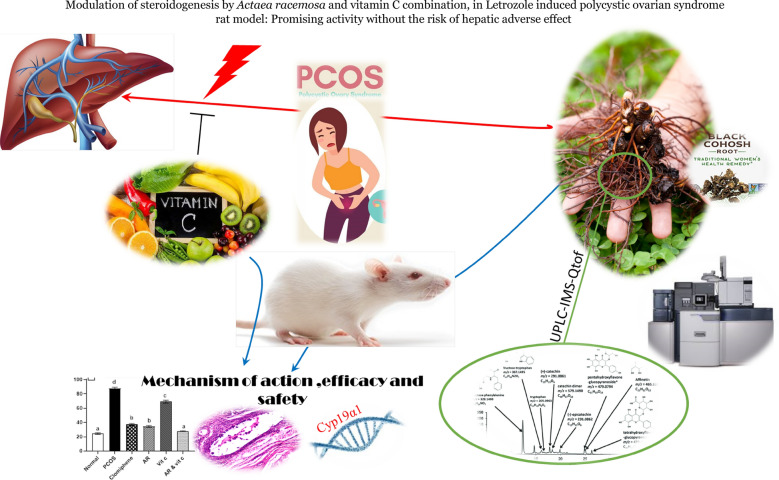

**Supplementary Information:**

The online version contains supplementary material available at 10.1186/s13020-021-00444-z.

## Background

Polycystic ovary syndrome (PCOS) is a complex metabolic–endocrine disorder [1] that affects 4–18% of women at the age of reproduction [2]. The genetic predisposition to PCOS is uncertain, and no genetic screening test has been validated [3]. PCOS is characterized by anovulation, menstrual irregularity, amenorrhea, hirsutism and infertility [2]. Furthermore, various metabolic and clinical complications have been reported such as insulin resistance and diabetes, obesity, extensive coronary artery disease, hypertension, endometrial hyperplasia, ovarian and breast cancers [4, 5]. The secretion and metabolism of estrogens and androgens are disturbed in PCOS [6]. The leading cause of PCOS is the excessive androgen level [7], that induced by overstimulation of ovarian theca cells by gonadotropin-releasing hormone (GnRH) [8].

Various side effects are associated with chemical and hormonal PCOS therapies such as hyperplasia, uterine bleeding and uncertain risks [9, 10] along with considerable cost [11]. Nonetheless, these therapies may not be effective in some cases. Therefore, several studies are focused on the investigation of complementary herbal medicine as a potential treatment of PCOS [9, 12–16]. For instance, promising effects of hops (*Humulus lupulus*), female ginseng (*Angelica sinensis*), ginseng (*Panax ginseng*), liquorice (*Glycyrrhiza glabra*), black cohosh (*Actaea racemosa*) and kelp (*Nereocystis luetkeana*) were reported against PCOS [17].

Black cohosh (*Actaea racemosa* (AR) or formerly; *Cimicifuga racemosa*) is a common herbal treatment in Europe, Asia, Australia, and America for a variety of women’s health disorders [18–21]. Although the previous testing has been undertaken with regards to the effects of AR for various gynecological conditions as oligo/amenorrhea, hyperandrogenism and PCOS, the limited quantity of data and subsequent variability in quality warranted further research to be undertaken [22, 23]. Recent studies revealed the beneficial effect of black cohosh for treating postmenopausal symptoms, however, there is a shred of conflicting evidence concerning its hepatic safety [24–26]. The Medical Products Agency in Sweden found that four of seven hepatotoxic cases were possibly associated with black cohosh products, but three of those were confused by the use of other liver injury-related drugs [27]. The consumption of black cohosh was correlated with acute liver injury [28]. Additionally, the analysis of the case reports from the Expert Committee of the US Pharmacopeia’s Council of Experts and others led to the recommendation of cautionary warning in regard to hepatotoxicity should be labeled for AR products [29, 30]. However, the Council made its analysis using the Naranjo scale, which has already then been considered not to be of use in the assessment of liver case reports. Still, none of the 30 examined cases were found to be indicative of a probable causality by AR, a finding that has later been confirmed using the Roussel Uclaf Causality Assessment Method (RUCAM) scale [30].

Protective action of vitamin C/ascorbic acid against hepatotoxicity has been reported [31, 32]. Moreover, antioxidants such as vitamin C have been associated with fertility enhancement depending on their role as a reducing agent [33–36]. Ascorbic acid has three reproductive actions, where it is needed for collagen biosynthesis, steroid and peptide hormone production and for the protection of cells from free radicals [34]. Ascorbic acid also showed a chemo-protective effect against degenerative changes in ovarian tissue [37].

The objectives of the present study were to determine the mechanism and molecular targets of AR for the treatment of PCOS along with any anticipated combination benefits with vitamin C and to investigate hepatic safety. In addition to the holistic metabolomics profiling via Ultra performance liquid chromatography (UPLC) coupled with high-definition mass spectrometry (HMDS) of AR where ion mobility spectroscopy (IMS) will be utilized for a higher confidence level of identification to support future chemometric and/or bioactive molecular network studies identifying potential active metabolites.

## Materials and methods

### Preparation of AR extract

Black cohosh roots and rhizomes were obtained from medicinal plant store (Haraz) in Cairo, Egypt and were authenticated at the herbarium of Botany Department, Faculty of Science, Cairo University, Giza, Egypt, using American Herbal Pharmacopoeia [38]. Plant sample (0.5 kg) was initially washed with running tap water followed by deionized distilled water, sun-dried and grounded, then was extracted by 70% ethanol (2 × 1.5 L) at 25 °C for 48 h. Solvents were evaporated under vacuum, in an evaporator (Büchi, Switzerland), at 45–55 °C and 60 rpm [39]. The obtained viscous extract was dried at 37 °C for 24 h, then weighed and percentage yields were calculated (7.6% w/w). The extract was stored at − 20 °C until use, where it was freshly dissolved in 0.5% carboxy methylcellulose (CMC) for the preparation of doses.

### UPLC–MS fingerprinting of the AR extract

The AR extract was dissolved in acetonitrile (2 mg/mL) and centrifuged at 13,000*g* for 15 min at 4 °C. The supernatant was filtered through a 0.2 μm PTFE filter while the first 2 mL were discarded. The metabolomic analysis was performed in ACQUITY UPLC (Waters, USA) coupled with SYNAPT G2-S high-definition mass spectrometry (Waters Corp, Manchester, England) as reported in the literature [40]. Briefly, a 5 µL sample was injected at a flow rate of 400 µL/min. Chromatographic separation was achieved using ACQUITY UPLC HSS T3 Column (1.8 μm, 2.1 × 150 mm, Waters Corporation, Milford, USA). The column temperature was kept at 45 °C and gradient elution was implemented utilizing water with 0.1% formic acid (A), and acetonitrile with 0.1% formic acid (B). Initially, 1% of the mobile phase B was used for 2 min, and linearly inclined as the following gradient: 35–60% B (2–4 min), 60–80% B (4–8 min), and 99% B (8–8.5 min) and finally declined to 10% B till 11.5 min.

G2-S high-definition mass spectrometer (HDMS) (Waters Corp, Manchester, England) equipped with Z-spray source controlled by MassLynx v4.1 was used for mass spectrometry analysis in both positive and negative ESI ionization modes using HDMS mode of operation. The scanning mode parameters were: source temperature; 120 °C, desolvation temperature; 500 °C, cone gas flow; 50 L/h, desolvation gas flow; 1000 L/h, collision energy ramp; 20–50 eV, capillary voltage; 2.5 kV, and acquisition mass range; 50–1200 m/z [41]. Data acquired in a profile mode and corrected with separate lock mass spray switching between the injected samples and external reference permitting the MassLynx to continuously ensure mass analysis accuracy. Leucine enkephaline (1 ng/μL) was used as an external reference in 1:1 acetonitrile–water containing 0.1% formic acid at a flow rate 5 uL/min via a lock-Spray interface, generating a reference ion for positive ion mode [M−H]^+^ and negative ion mode [M−H]^−^ of 556.2771 and 554.261 m/z, respectively [42].

Three technical replicates were implemented in a randomized batch sequence. To enable proper column equilibration and conditioning, the mobile phase was run for 1.5 h, followed by six quality control (QC) samples before each batch analysis. In line with the published guidelines and to overcome the UPLC–MS analytical drifts, QC samples were injected at regular intervals during the experimental sequence [43]. Features were considered reproducible if their coefficient of variation (CV) among the sample replicates were < 25%, and the fold change (FC) > 2, ANOVA P-value and Q value < 0.01 against blank samples Q value is an adjusted P-value, calculated using an optimized false discovery rate (FDR) approach.

Progenesis QI software (Waters Corp., USA) was used for data processing and putative identification of metabolites of interest by comparison with literature and available databases such as metabolomic profiling CCS library (Waters Corp., USA), LipidBlast and Progenesis MetaScope imported databases including HMDB, MONA, and GNPS and Chemspider imported data sources such as NIST and PlantCys.

### In vivo experiments

The experimental protocol was approved by the Institutional Animal Care and Use Committee of Cairo University (CU-IACUC; VetCU11112018017).

Virgin, cyclic, adult female Wistar Albino rats (160–200 g) were used in this study. Animals were acquired from Laboratory Animal Colony, Helwan, Egypt, and housed in the animal house of the Faculty of Veterinary Medicine, Cairo University, Egypt, and allowed to acclimatize for 2 weeks. During the study, all animals were caged in standard polypropylene cages and maintained in a controlled environment of (22 ± 3) °C temperature, (55 ± 5) % humidity and a 12 h light/dark cycle. The rats were provided with a standard diet and water ad libitum.

### Experimental protocol

Forty-eight female rats were randomly allocated into six groups of eight each. Animals of group one served as negative control and received a daily oral dose of (1 mL) of the vehicle (0.5% CMC) using gavage for 49 days. The induction of PCOS was done guided by an established rat model that was previously described [44]. Animals of groups two to six received letrozole (LTZ) (Natco Pharma Limited Hyderabad) at a dose of 1 mg/kg dissolved in 0.5% CMC once daily for 21 days for induction of PCOS. Different samples (vehicle, standard and extracts) were then received orally for 28 days. Group 2 (positive control) received only the vehicle and Group 3 received clomiphene citrate in 0.5% of CMC at a dosage of 1 mg/kg (Fertyl-Super^®^ tablets were obtained from Ar-Ex Laboratories Private Limited, Goregaon (E), Mumbai) as a standard ovulation induction drug [45]. Groups (4–6) were treated for 28 days with AR extract; 7.14 mg/kg, vitamin C; 500 mg/kg and AR extract with vitamin C, respectively. From day 6 of treatment, daily vaginal smears of all rats were obtained for ovulation monitoring, where PCOS was suggested by an indiscriminate estrous cycle with a prolonged diestrus phase [46, 47]. On day 50 of the study, all the rats were anesthetized with ketamine 91 mg/kg, i.p. Duplicate blood samples were collected in sodium heparin tubes for separation of plasma and in gel separator tubes for collection of serum samples. The serum was separated by blood centrifugation at 3000*g* at 4 °C for 10 min and used for different biochemical assays. The animals were then sacrificed, ovaries and uteruses excised, cleaned of fat, weighed and divided into triplicates; two sets; stored at − 80 °C to be used for real-time reverse transcriptase-PCR and antioxidant assays. Another set of ovaries in addition to liver tissue were fixed in 10% neutral buffered formalin for histopathological examination. The relative weight of the ovaries was calculated as the ratio of the ovary (wet weight in mg) to the body weight (g) for each animal on the day of scarification.

### Hormonal profile

Serum total testosterone was measured using a commercial ELISA kit (Chemux bioscience Inc, USA) and the beta subunit chain of luteinizing hormone (LH) level was measured using rat lutropin subunit beta ELISA kit (EIAab, China) following the instructions of the manufacturers.

### Malondialdehyde (MDA) and antioxidants biomarkers determinations

Ovarian and liver tissues were separately homogenized in 10 mL cold buffer per gram tissue using a glass homogenizer then tissue homogenate was centrifuged at 5000 rpm for 15 min at + 4 °C. This buffer consists of 50 mM potassium phosphate with a pH 7.5 for MDA and 50 mM potassium phosphate with a pH 7.5 and 1 mM ethylenediaminetetraacetic acid (EDTA) for reduced glutathione (GSH). The supernatant was used to measure GSH and MDA concentrations according to the standard protocols [48, 49].

### Biochemical parameters

Serum glucose level, lipid profile [cholesterol, triglycerides, and high-density lipoproteins (HDL)] and liver enzymes [alanine aminotransferase (ALT) and aspartate aminotransferase (AST)] were measured using commercial kits (Spectrum, Egypt). The very low-density lipoprotein (VLDL) and low-density lipoprotein (LDL) concentration was calculated as follows:

$$\left( {{\text{VLDL}}} \right) = {\text{triglycerides}}/{5}$$

$$\left( {{\text{LDL}}} \right) = {\text{total cholesterol}}{-}\left( {{\text{HDL}} + {\text{VLDL}}} \right)$$

### Histopathological examination

Ovaries and liver from different groups were collected and fixed in 10% neutral-buffered formalin and processed to obtain 3–4 µm paraffin-embedded sections. The sections were stained with hematoxylin and eosin (H&E) and the morphometric analysis of the ovaries was performed [50, 51]. The number of follicular cysts, its average and diameter alongside the thickness of granulosa cell layer and the thickness of theca cell layers were measured.

### Immunohistochemical analysis

The tissue sections were deparaffinized, rehydrated and pre-treated with 10 mM citrate buffer for antigenic retrieval. Sections were incubated overnight at 4 °C in a humidified chamber with the following primary antibody: rabbit monoclonal anti-Ki 67 antibody (Dako, Denmark) in 1:25 dilution. The tissue sections were incubated with a biotinylated goat anti-rabbit and mouse antibody (Thermo Scientific, USA), streptavidin peroxidase (Thermo scientific, USA) and 3,3′-diaminobenzidine tetrahydrochloride (DAB, Sigma). The slides were counterstained with Mayer’s hematoxylin then dehydrated and mounted. The primary antibody was replaced by PBS for negative controls. The stained sections were analyzed by Leica Qwin 500 Image Analyzer (Leica, Cambridge, England). In each field, the immunolabeled dark brown areas were recorded. The percentage of positively stained area in the granulosa cell layer, theca cell layers and interstitial stromal cell layer was calculated [52].

### Quantitative real-time PCR for aromatase (Cyp19α1) gene

Total RNA was extracted from female rat ovarian tissue samples using RNeasy mini kit (Qiagen, Hilden, Germany). The quantity and purity of the total RNA were measured by the NanoDrop spectrophotometer. The cDNA synthesis was carried out using reverse transcriptase (Invitrogen, California, USA) and oligo-dT following the manufacturer protocol. The cDNA samples were then submitted to qPCR using the following forward (5′-CTGCTGATCATGGGCCTCCT-3′) and reverse (5′-CTCCACAGGCTCGGGTTGTT-3′) primer pairs for Cyp19α1 gene. The cDNA was amplified by 40 cycles of denaturation at 95 °C for 45 s, annealing at 59 °C for 45 s and extension at 72 °C for 45 s. The 95 °C step was extended to 4 min during the first cycle. The amplicon size was confirmed by 2% agarose gel electrophoresis stained with SYBR Safe DNA gel stain (Invitrogen). The β-actin gene was furtherly amplified in the same reaction to serve as the reference gene [53]. Each measurement was repeated three times, and the values were used to calculate the gene/β-actin ratio, with a value of 1.0 used as the control (calibrator) and the normalized expression ratio was calculated [54].

### Statistical analysis

The different analytical measurements in the biological samples were carried out in triplicate and results are expressed as the mean ± SD, ^a, b, c, d^ superscripts indicate statistical significance compared to the negative control group at P < 0.05 (n = 5). Data for multiple variable comparisons were analyzed by one-way analysis of variance (ANOVA) test to analyze the significant differences (P < 0.05) between groups using SPSS version 24 package for Windows.

## Results

### Identification of metabolites via UPLC-QTOF-MS

Metabolites were tentatively identified based on their accurate mass, isotopic distribution, fragmentation patterns, comparison with common mass libraries alongside the dictionary of natural products (CRC Press, Taylor & Francis Group) and the reported literature. The untargeted metabolomic analysis enabled the putative identification of 61 compounds in both ionization modes and their structures were allocated as 13 flavonoids and glycosides, 12 fatty acids/lipids, 10 tannins, seven triterpenes, six steroids, two miscellaneous terpenoids, two carbohydrates and one phenolic acid derivative (Additional file 1: Table S1, Figure S1–S43).

### Relative ovarian and uterine weights

**Fig. 1:**
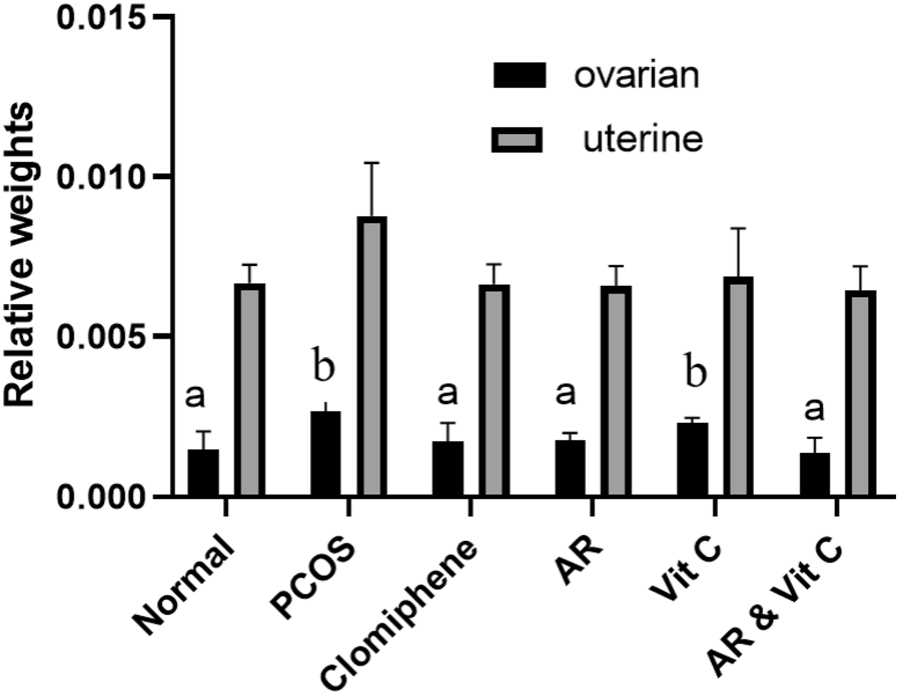
Effect of *Actaea racemosa* (AR) extract and/or vitamin C on relative ovarian and uterine weights in letrozole induced polycystic ovary syndrome (PCOS) rat model. Values are presented as Mean ± SD, where means carrying different letters are significantly different at P < 0.05 (n = 5)

In general, the relative weights of both organs were reduced in all groups after treatment compared to the PCOS group. Nevertheless, these differences were significant for ovaries and insignificant for the uterus (Fig. 1).

### Hormonal profiles

**Fig. 2:**
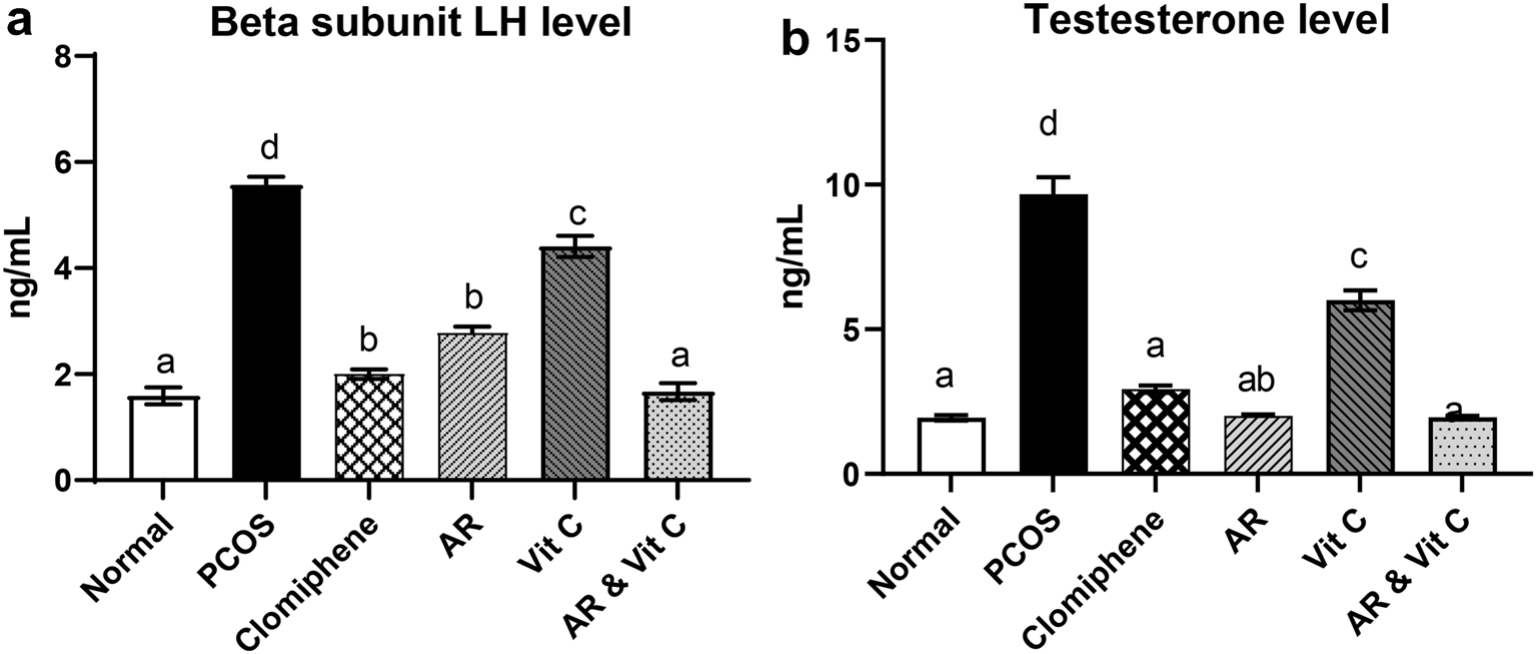
Effect of *Actaea racemosa* (AR) extract and/or vitamin C on **a** Beta subunit LH level **b** testosterone level in letrozole induced polycystic ovary syndrome (PCOS) rat model. Values are presented as Mean ± SD, where means carrying different letters are significantly different at P < 0.05

Induction of PCOS significantly increased LH and testosterone concentrations compared to the negative control group. However, rats treated with AR extract and its combination with vitamin C for 28 days showed significantly lowered LH and testosterone levels. The combination group provoked a superior hormonal effect, in contrast to vitamin C which displayed the least effect (Fig. 2a, b).

### Oxidative stress biomarkers in ovary and liver tissues

**Fig. 3:**
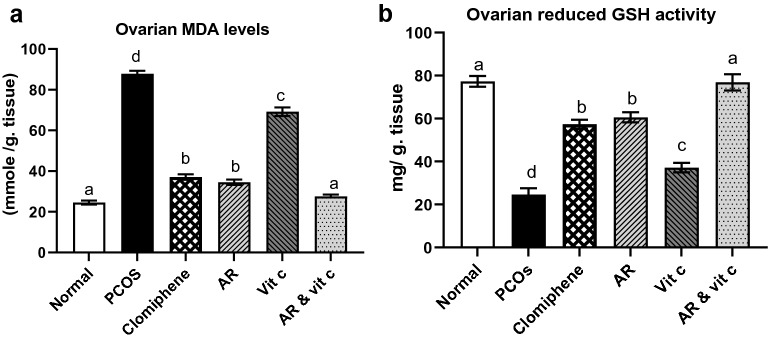
Effect of *Actaea racemosa* (AR) extract and/or vitamin C on ovarian **a** MDA level **b** GSH activity in letrozole induced polycystic ovary syndrome (PCOS) rat model. Values are presented as Mean ± SD, where means carrying different letters are significantly different at P < 0.05 (n = 5)

**Fig. 4:**
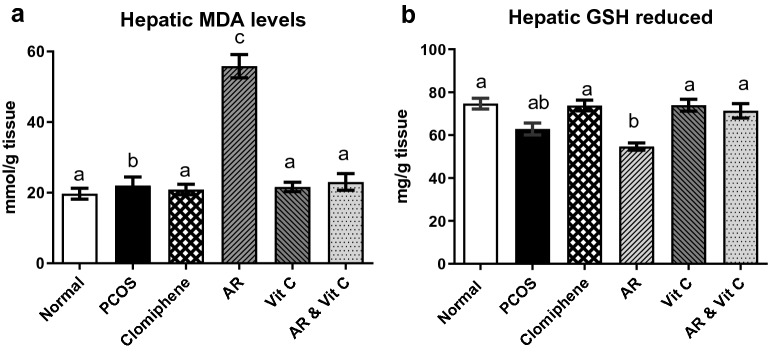
Effect of *Actaea racemosa* (AR) extract and/or vitamin C on hepatic **a** MDA level **b** GSH activity in letrozole induced polycystic ovary syndrome (PCOS) rat model. Values are presented as Mean ± SD, where means carrying different letters are significantly different at P < 0.05 (n = 5)

Induction of PCOS significantly increased MDA levels, while GSH activity was significantly decreased compared to the negative control. *Actaea racemosa* hydroethanolic extract with and without vitamin C restored normal ovarian MDA and GSH activities (Fig. 3a, b). Figure 4a, b demonstrated the hepatic MDA and GSH activities, where the exerted mild hepatic oxidative stress by AR and to less extent by LTZ in the PCOS model group, was prevented in the combination group (AR with vitamin C).

### Biochemical parameters

The changes in lipid profile (total cholesterol, triglyceride, LDL cholesterol, VLDL cholesterol), glucose concentrations, and the activities of ALT and AST were reported in Table 1. Interestingly, ALT and AST activities were significantly increased in both AR and PCOS groups when compared with the negative control group. Nonetheless, they were significantly decreased in the vitamin C group and to a lesser extent in the combination group.

**Table 1:** Effect of *Actaea racemosa* (AR) extract and/or vitamin C on lipid profile, glucose level, liver function tests in letrozole induced polycystic ovary syndrome (PCOS) rat model

Parameters	Normal	PCOS	Clomiphene	AR	Vitamin C	AR and Vitamin C
Cholesterol (mg/dL)	104.8 ± 4.5^a^	148.78 ± 1.6^c^	141.5 ± 3.2^c^	143.3 ± 5.94^c^	115.9 ± 2.64^b^	121.8 ± 4.73^b^
Triglyceride (mg/dL)	44.03 ± 4.1^a^	72.24 ± 3.81^d^	74.51 ± 4.1^d^	70.99 ± 3.69^d^	53.52 ± 0.86^b^	67.23 ± 3.11^c^
HDL (mg/dL)	76.57 ± 3.1^a^	58.42 ± 1.1^c^	57.66 ± 1.8^c^	55.11 ± 5.08^c^	66.23 ± 1.38^b^	63.44 ± 3.02^b^
LDL (mg/dL)	19.46 ± 2.2^a^	75.92 ± 0.4^c^	73.54 ± 1.12^c^	72.14 ± 1.49^c^	29.17 ± 1.03^b^	31.69 ± 2.13^b^
VLDL (mg/dL)	8.80 ± 0.8^a^	14.44 ± 0.8^c^	15.30 ± 0.92^c^	15.79 ± 0.74^c^	10.5 ± 0.17^b^	11.65 ± 0.62^b^
Glucose (mg/dL)	82.06 ± 4.3^a^	128.4 ± 0.8^c^	123.03 ± 1.39^c^	130.13 ± 2.5^c^	104.4 ± 2.9^b^	107.34 ± 1.2^b^
ALT(U/L)	19.27 ± 1.1^a^	36.32 ± 3.76^c^	31.44 ± 2.1^b^	94.85 ± 6.20^d^	22.35 ± 1.77^a^	24.22 ± 1.89^ab^
AST (U/L)	34.16 ± 1.3^a^	51.43 ± 4.85^c^	44.46 ± 2.47^b^	93.96 ± 7.14^d^	36.41 ± 3.17^a^	40.82 ± 2.2^ab^

Values are presented as Mean ± SD, where means carrying different superscript letters are significantly different at P < 0.05 (n = 5)

### Histopathology of ovaries

**Fig. 5:**
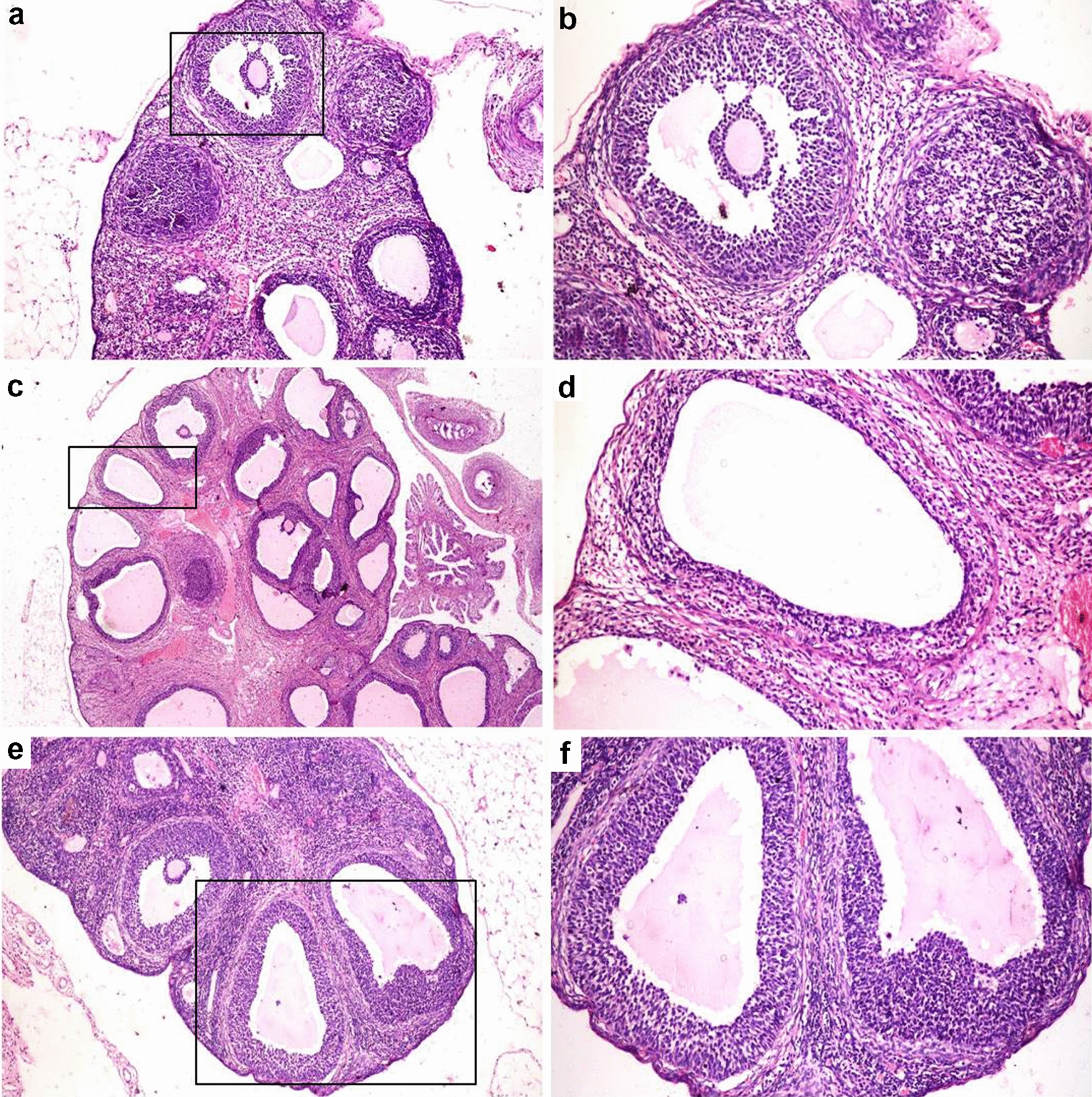
Photomicrographs of hematoxylin and eosin stained sections of ovaries in different experimental groups. Rectangle refers to an area with higher magnification in the next picture. **a** Ovary of negative control group showing the normal histological picture. **b** Normal secondary follicle. **c** Ovary of PCOS group showing multiple ovarian cysts. **d** Ovarian cyst with a thin layer of flattens granulosa cells. **e**, **f** Clomiphene treated group showing a reduction in the number of ovarian cysts and restoration of granulosa cell thickness

**Fig. 6:**
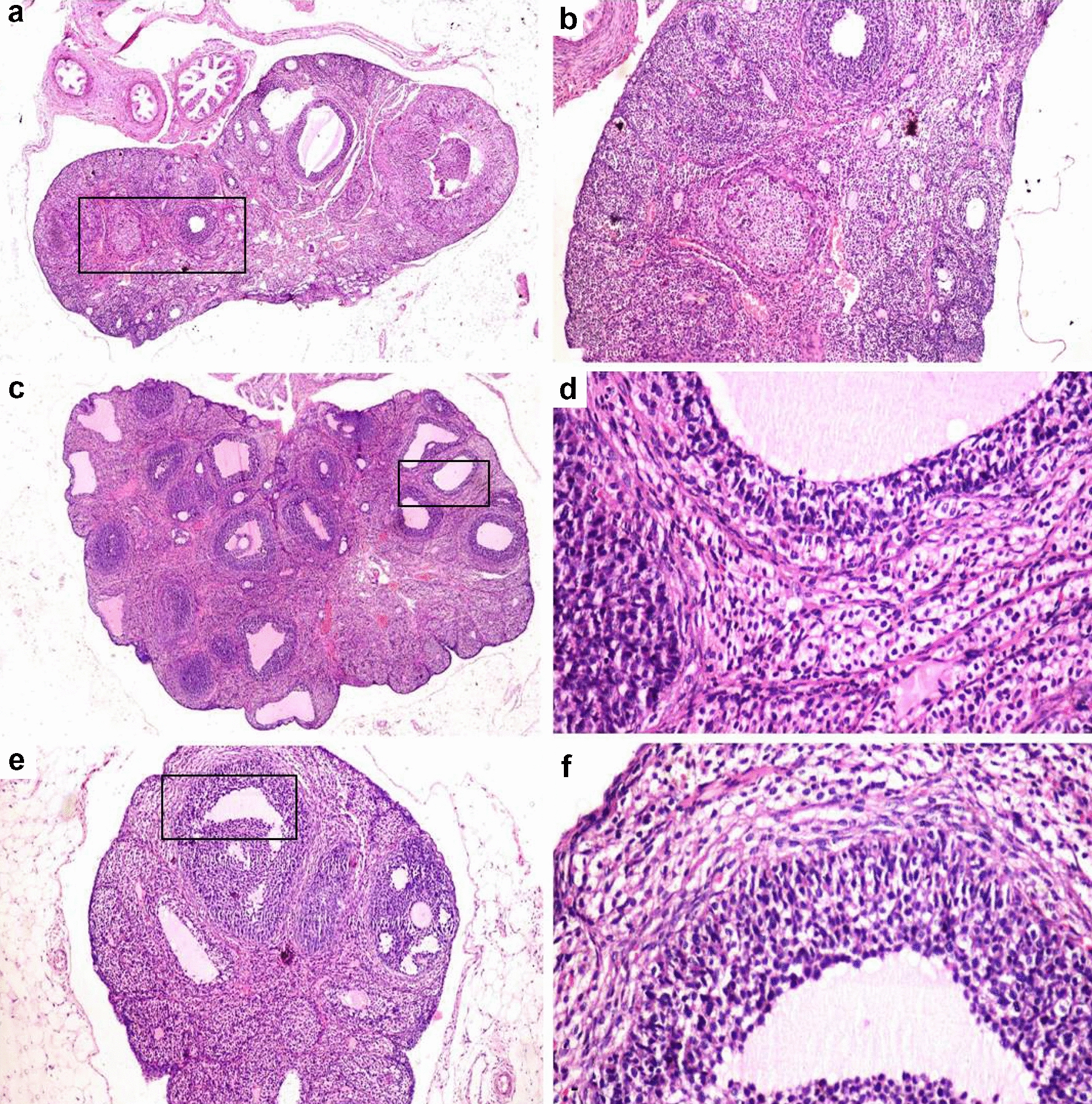
Photomicrographs of hematoxylin and eosin stained sections of ovaries in different experimental groups. Rectangle refers to an area with higher magnification in the next picture. **a** Ovary of *Actaea racemosa* (AR) group showing a marked reduction in the number of ovarian cysts with the presence of growing follicles. **b** Presence of corpora lutea. **c** Ovary of vitamin C treated group showing polycystic ovary. **d** Ovarian cyst with degenerated and necrosed granulosa cell layer. **e** *Actaea racemosa* (AR) combined with vitamin C treated group showing a marked reduction in the number of cysts with the presence of secondary follicles and corpora lutea. **f** Follicle showing proliferated granulosa cell layer

**Fig. 7:**
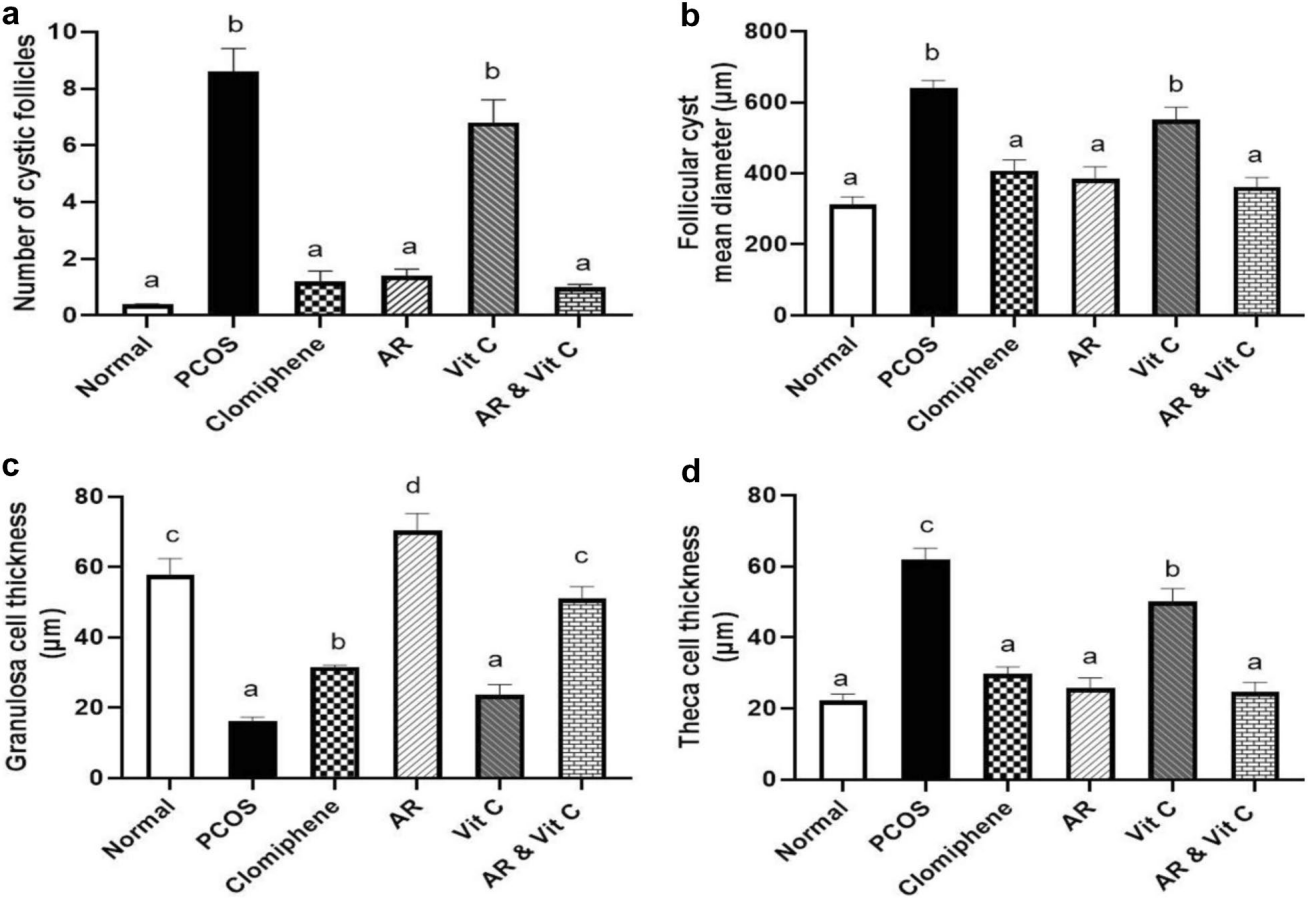
Morphometrical analysis of ovaries in the different treated groups. **a** Number of ovarian cysts per ovary. **b** Mean diameter of follicular cysts. **c** Granulosa cell thickness. **d** Theca cells thickness. Values with different superscripts are significantly different (P < 0.05). PCOS; polycystic ovary syndrome, AR; *Actaea racemose*, Vit. C; vitamin C

The negative control group showed normal ovary histology in the form of multiple follicles at different stages of development with normal granulosa, theca and interstitial stromal cell layers in addition to presence of various corpora lutea (Fig. 5a, b). PCOS group revealed numerous ovarian cysts in addition to small follicles in the early developmental stage and without any evidence of the existence of corpora lutea (Fig. 5c). The follicular walls of the cystic follicle contained a very thin layer of flattening granulosa cells (Fig. 5d) that most of them exhibited necrosis and apoptosis. The theca cell layers showed marked proliferation of theca interna and theca externa cells. Some ovarian cysts did not contain any granulosa cells with only theca interna and theca externa cells. The granulosa cell layers of other ovarian cysts were hyperplastic and folded. The interstitial stromal cell layer was very thick with proliferated and hyperplastic cells forming a cord-like structure. The same ovarian architecture was observed in the vitamin C treated group (Fig. 6c, d). The groups treated with clomiphene (Fig. 5e, f), AR extract (Fig. 6a, b) and AR combined with vitamin C (Fig. 6e, f) restored the ovarian functions and showed a reduction in the number of ovarian cysts, restoration of granulosa cell thickness and presence of corpora lutea. Furtherly, the morphometric analysis of ovaries in the different experimental groups revealed a significant reduction in the number and diameter of ovarian cysts with a significant restoration of the granulosa cell layer thickness and a significant reduction in both theca cells compared to PCOS treated groups (Fig. 7a–d).

### Immunohistochemical analysis of Ki-76 expression in ovaries

**Fig. 8:**
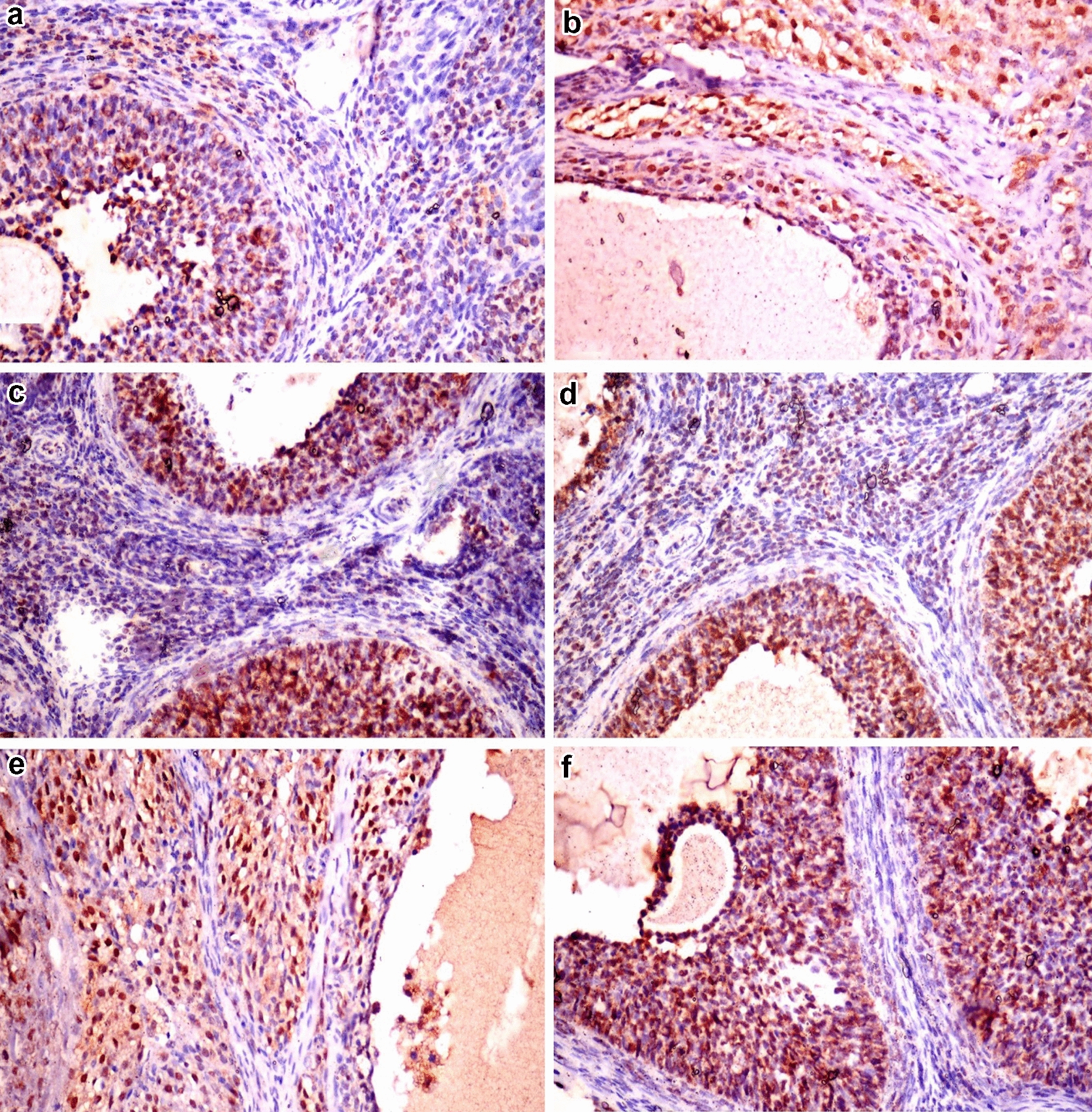
Photomicrographs of regions of ovaries showing immunohistochemical expression of Ki-67 in the different treated groups (×200). **a** Negative control group showing strong immunopositive staining in the granulosa cell layer with weak immunoreactivity in the theca cells and stromal cells. **b** PCOS group showing strong immunopositive reaction in theca cells and interstitial stromal cells. **c** Clomiphene treated group showing strong immunopositive staining in the granulosa cell layer. **d** *Actaea racemosa* (AR) treated group showing strong immunolabeling in granulosa cell layer and slight immunopositive staining in theca cells and stromal cells. **e** Vitamin C group showing intense immunolabeling in the theca cells and interstitial stromal cells. **f** AR combined with vitamin C group showing very strong immunopositive staining in granulosa cell layer with a faint reaction in the theca cells and interstitial stromal cells

**Fig. 9:**
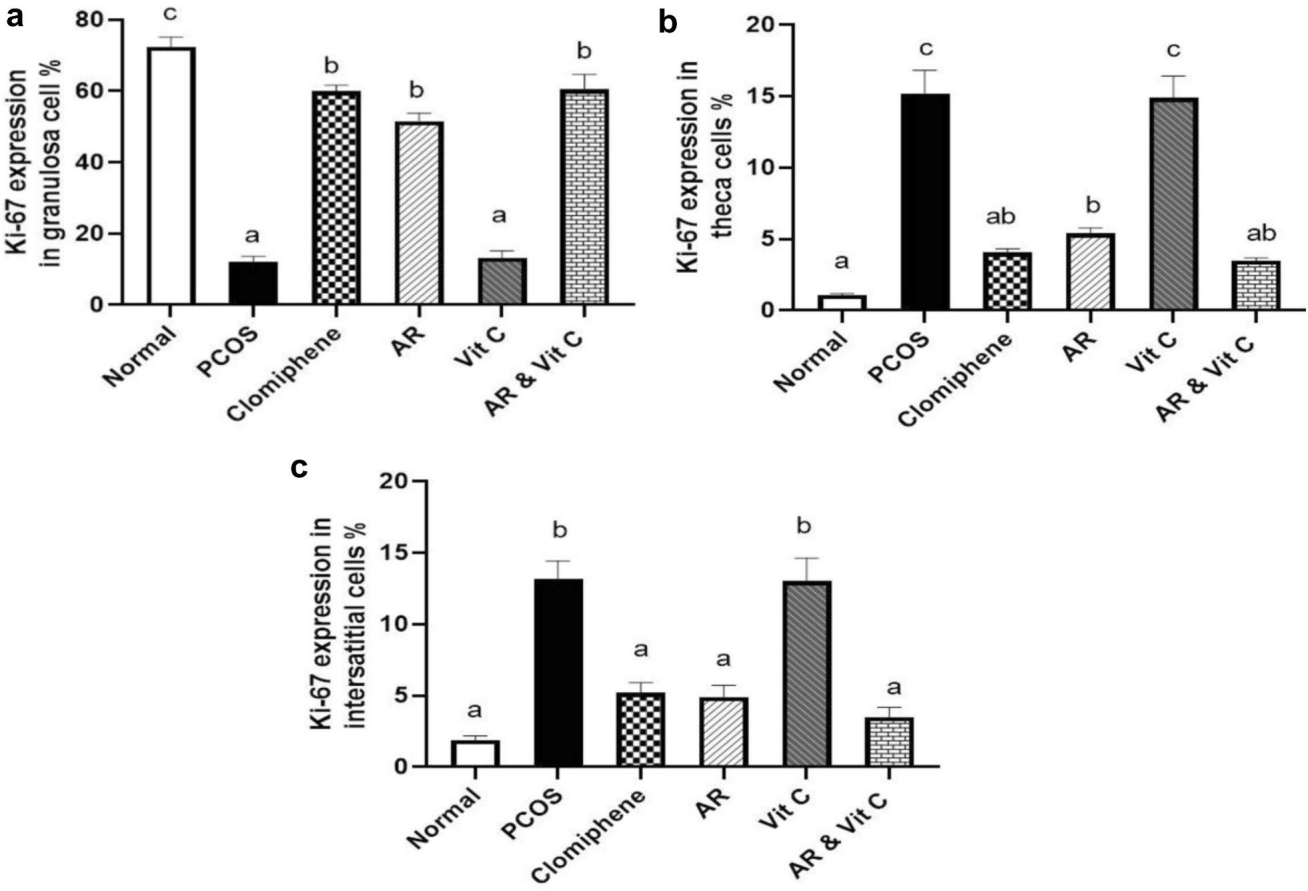
Morphometrical analysis of the percentage of Ki-67 expression in the ovaries of the different treated groups. **a** Ki-67 expression in the granulosa cell layer. **b** Ki-67 expression in the theca cell layers. **c** Ki-67 expression in the interstitial stromal cells. Values with different superscripts are significantly different (P < 0.05). PCOS; polycystic ovary syndrome, AR; *Actaea racemose*, Vit. C; vitamin C

Figure 8 summarizes the results of immunohistochemical analysis of Ki-67, a cell proliferation marker and its expression in the granulosa cell, theca cell and interstitial stromal cells of the ovaries among different groups. Ovaries in the negative control group showed strong immunopositivity to Ki-67 expression in the granulosa cell layer of the follicles (Fig. 8a). Both PCOS and vitamin C treated groups revealed weak immunoreactivity in the granulosa cell layer and strong immunopositivity in the theca cell and interstitial stromal cell layers (Fig. 8b, e). The groups treated with clomiphene, AR and AR combined with vitamin C showed strong immunopositivity in the granulosa cell layer of follicles with weak immunoreactivity in the theca cell and interstitial stromal cell layers (Fig. 8c, d and f). The morphometric analysis of different regions of the ovary was performed in the different treated groups. Notably, a significant increase in the percentage of Ki-67 expression in the granulosa cell layer (Fig. 9a) was demonstrated in the groups treated with clomiphene, AR and AR with vitamin C compared to the PCOS group unlike its significantly decreased expression in both theca cells and interstitial cells (Fig. 9b, c).

### Histopathological examination of liver

**Fig. 10:**
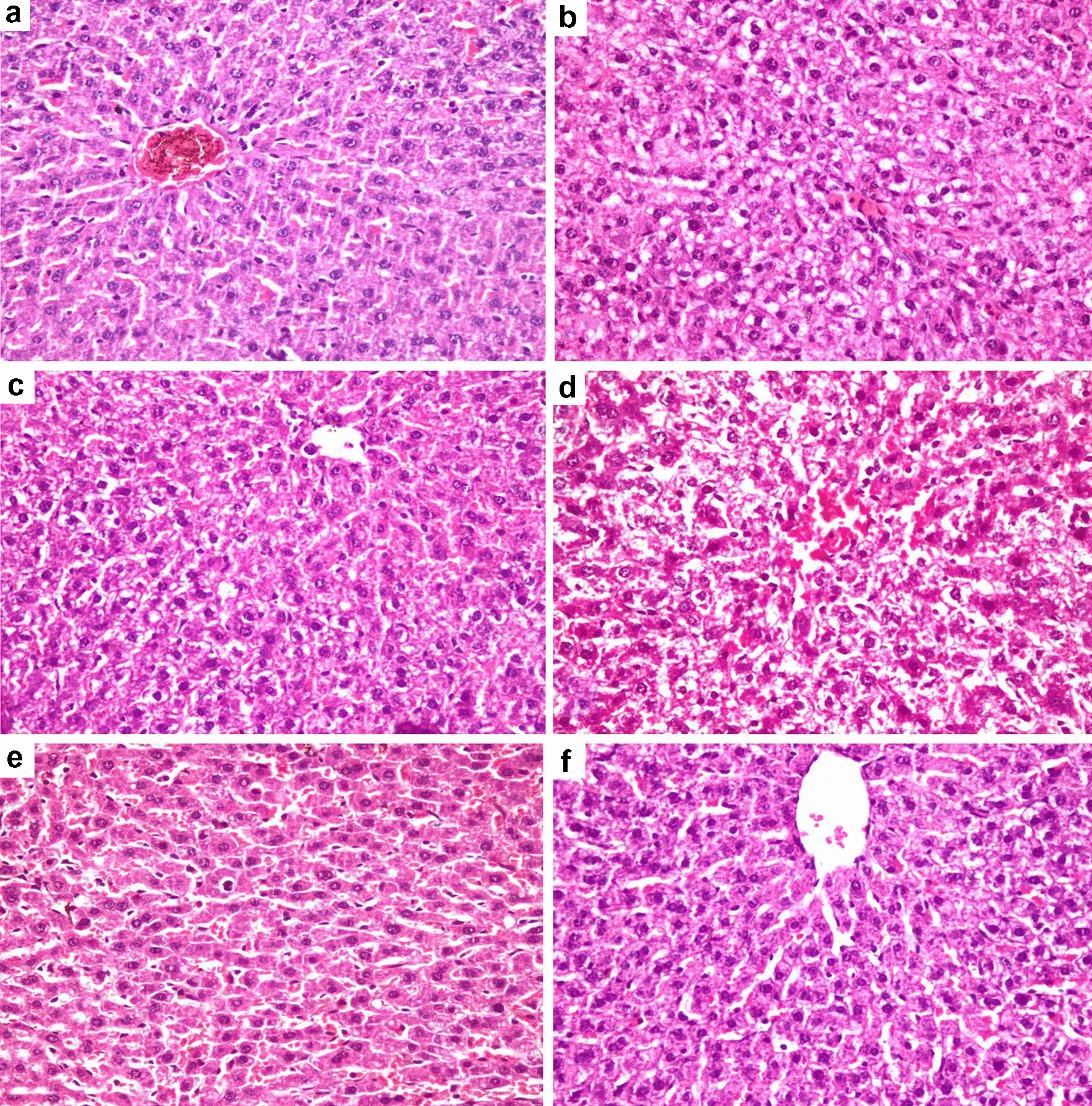
Photomicrographs of hematoxylin and eosin stained sections of the liver in the different treated groups (×400). **a** Negative control group showing normal hepatic architecture, **b** PCOS group showing moderate vacuolation of hepatocytes. **c** Clomiphene treated group showing mild vacuolation of hepatocytes. **d** *Actaea racemose* (AR) treated group showing hepatocellular necrosis with sinusoidal dilation and disorganization of hepatic cords. **e** Vitamin C group showing normal histological findings. **f** AR combined with vitamin C group showing mild vacuolation of hepatocytes with sporadic cell necrosis

The livers of the negative control and vitamin C treated groups revealed a normal hepatic architecture (Fig. 10a, e). The livers of PCOS and clomiphene treated groups showed moderate and mild vacuolar degeneration with single hepatocyte necrosis, respectively (Fig. 10b, c). The liver of AR treated groups revealed moderate vacuolar degeneration, apoptosis, hepatocellular necrosis (Fig. 10d) and sinusoidal dilation with leukocytosis. However, the group treated with AR combined with vitamin C exhibited marked improvement of the previously described hepatic lesions induced by AR extract treatment alone. Additionally, mild vacuolar degeneration and sporadic cell necrosis were demonstrated in the combination-treated group (Fig. 10f).

### Quantitative real-time PCR for aromatase Cyp19α1

**Fig. 11:**
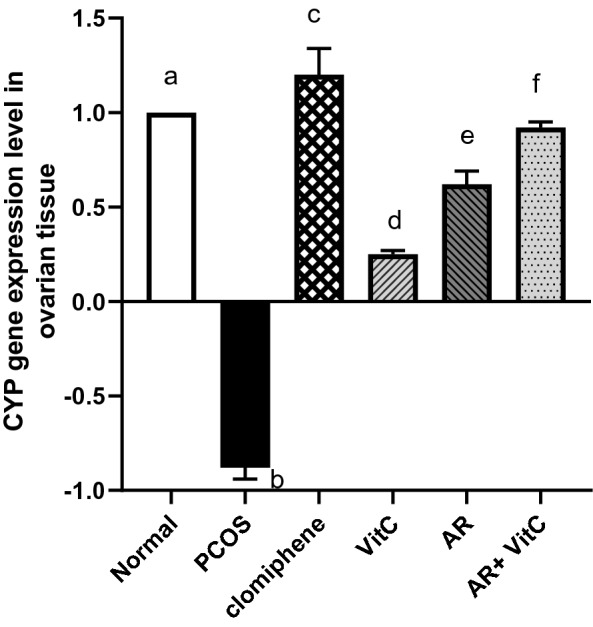
Effect of *Actaea racemose* (AR) extract and/or vitamin C on the expression level of Cyp19α1 gene in polycystic ovary syndrome (PCOS) rat model. Values are presented as mean ± SD. Means carrying different letters are significantly different at P < 0.05. PCOS; polycystic ovary syndrome, AR; *Actaea racemose*, Vit. C; vitamin C

Real-time PCR was implemented to evaluate the mRNA expression level of the aromatase enzyme which is a key enzyme in the steroid biosynthesis pathway. The quantitative real-time PCR analysis for the Cyp19α1 gene in ovarian tissue from different groups showed that its mRNA expression level was significantly downregulated in the PCOS group compared to the negative control. However, the groups treated with AR extract and/or vitamin C significantly upregulated the mRNA expression level for Cyp19α1 where the combination treatment was more effective (Fig. 11).

## Discussion

Regardless the growing literature on the AR effectiveness for menopausal symptoms, definite assumptions on its exact mechanism of action cannot be deduced from clinical studies not only related to blinding issues, but also to the fact that such things require a very special study design [55]. In the present study, the plethora of active secondary metabolites such as triterpenes, steroids, flavonoids, and tannins of the black cohosh root ethanolic extract have been tentatively identified using UPLC-HDMS (Additional file 1: Table S1). An extra dimension of separation was achieved via IMS based on the size, shape and charge of the analytes, where the characteristic CCS values were reported and could be used in combination with other molecular identifiers to enhance the confidence level of metabolite recognition.

The efficacy of LTZ rat model in the induction of PCOS is well established and represents the human PCOS in various aspects [16, 56, 57] where the oxidative stress may be associated with the pathogenesis of PCOS [58]. The oxidative stress induced by LTZ in ovaries [59] was similar to that observed in natural PCOS [6]. Our results showed a significant increase in MDA level and reduction in GSH activity in the PCOS model, which may be attributable to the increased oxidation of biomolecules resulting in excessive peroxidation and consequent tissue deterioration by ROS-mediated mechanisms [60]. The provoked enhancement in ovarian MDA and GSH activities by AR may be associated with its enzymatic antioxidant mechanisms for PCOS treatment. Furthermore, the effect of AR and vitamin C combination on the regulation of ovarian MDA and GSH was statistically significant compared to the single treatments (Fig. 3).

In LTZ model of PCOS, the conversion of androgen substrates into estrogens in the granulosa cells was blocked leading to an accumulation of androgen [56]. As a result, early luteinization of the ovarian granular cell layer and a cessation of follicular development with eventual anovulation or poor ovulation will be anticipated [61]. Moreover, theca cell layers are responsible for the synthesis of androgen [62]. In our study, LTZ treated group exhibited numerous ovarian cysts without any evidence of corpora lutea where the follicular walls contained a very thin layer of flattening granulosa cells with a marked proliferation of both theca interna and theca externa cells contributing to hyperandrogenism (Fig. 6), similar findings have previously been published [44, 51]. Interestingly, both the AR and the combination-treated groups restored the ovarian functions of LTZ induced-PCOS model shown by the restoration of granulosa cell thickness, the reduction of both thecal cell layers and the presence of corpora lutea.

Previous studies have shown that ovarian cysts were formed by apoptosis of both ovarian oocytes and granulosa cells, where Ki-67 can be used as an indicator for cellular proliferation [63]. In our work, we observed a higher expression of Ki-67 in the theca cell layers responsible for excess androgen production and in the interstitial cells in the PCOS group [44]. The groups treated with AR with and without vitamin C showed significant intense immunolabeling of Ki-67 in the granulosa cell layer with weak immunolabeling of Ki-67 in the theca cell layers and interstitial cells, suggesting their role in the reduction of the high androgen level induced by PCOS. This finding outlined that AR could be a useful therapeutic approach in the case of PCOS by protecting the granulosa cell layer from apoptosis and necrosis and it could help in their maintenance and proliferation with androgen level reduction.

The androgen aromatization to estradiol in dominant follicles is conducted in the granulosa cells by the aromatase enzyme. Aromatase (Cyp19α1) is a key steroidogenic enzyme that separately catalyzes the conversion of testosterone to estradiol and estrone. Aromatase is encoded by the Cyp19α1 gene [64]. Intriguingly, the Cyp19α1 encoding gene can be included as a major risk determinant for PCOS [65]. Our findings unveiled a significant downregulation of Cyp19α1 mRNA expression level in PCOS ovary tissues. These results were consistent with previously published data [16, 65, 66]. Remarkably, the AR and vitamin C combination showed superior and significant gene upregulation versus single treatments. Cyp19α1 expression could be regularly suppressed in PCOS ovaries owing to the promoter hypermethylation such as the promoter hypermethylation of Cyp19α1 which may play a key role in the PCOS pathogenesis [67]. Also, testosterone was reported as a crucial factor responsible for aromatase downregulation in PCOS with the downregulation of both aromatase mRNA and protein levels in cultured luteinized granulosa cells [66]. The present study proposed the reversal of PCOS downregulation of the steroidogenic Cyp19α1 gene expression as a possible contributing mechanism for the beneficial effects of AR on PCOS with superior effect when combined with vitamin C.

The reported elevation in testosterone and LH hormones in LTZ induced PCOS rat models [9, 68, 69] were significantly restored by AR (Fig. 2a, b). Similar data were reported on the ovariectomized rats [70] and isolated cells from ovariectomized rats [22, 71]. That was probably mediated by interference with the LTZ inhibitory effect on androgen aromatization, preventing the excessive androgens accumulation in the ovaries as mentioned before. Moreover, AR has an estrogen-like effect, where it acts directly on the hypothalamus to decrease GnRH and the subsequent reduction of LH hormone [72, 73].

The hormonal disorders in PCOS are combined with metabolic disorders. High testosterone concentrations in PCOS lead to pancreatic β cell dysfunction, insulin resistance, and thus hyperglycemia and dyslipidemia [74, 75], in addition to the antilipolytic effects of androgens [76]. Consistently, our results showed hyperglycemia and significant elevation in all lipid profile parameters such as cholesterol, triglyceride, LDL, and VLDL with a declined HDL level in LTZ induced PCOS rats compared with negative control (Table 1), in agreement with other published reports [77–79]. Hyperglycemic and dyslipidemic effects observed in black cohosh rats were related to PCOS, while AR did not affect the lipid profile and glucose in women [80, 81]. However, vitamin C exerted an obvious enhancement in glucose and lipid metabolism, and increased the HDL levels, which were previously detected in both diabetic patients [82] and rats [83].

The dose of 500 mg/kg for vitamin C and 7.14 mg/kg for AR was selected based on previous studies [84, 85]. This selected dose of AR was also formerly implemented to study its effects on PCOS [86] and breast cancer [87] in rats and its equivalent was used in clinical trials in women [81]. Noteworthy, the No Observed Effect Level (NOEL) of AR was defined as 22.5 mg/kg b. wt. in 6-months oral toxicity study with its isopropanolic extract in Wistar rats [87]. AR extract doses of 0.6–2.25 mg/kg b. wt. in rats is like those recommended to postmenopausal women and PCOS which is 40–150 mg/day, considering a woman weighing 60 kg [24, 73, 88, 89].

Hepatic safety of AR is still a debatable issue. Although, we observed a significant increase in the ALT, AST and hepatic MDA levels alongside with a reduction in hepatic GSH activity in the AR group when compared to the negative control, vitamin C and the combination group. It was not dramatic changes as supported by the pathological finding of only mild hepatocellular necrosis with sinusoidal dilation and disorganization of hepatic cords (Table 1, Figs. 4a, b, 10d). Similar findings for AR induced hepatic oxidative damage were reported [28, 90, 91], which were contradicted in other studies [26, 92]. Increased hepatic lipid peroxidation with AR treatment was previously observed [89]. Although, no reduction was observed in the hepatic GSH with AR 0.6 mg/kg b. wt. in ovariectomized female rats, a marked reduction in the hepatic GSH of male rats treated with AR (300 mg/kg) was previously recorded [93]. This discrepancy could have been related to lower dosing and hyperandrogenism [89]. In addition, PCOS group also showed a minor hepatic changes demonstrated by elevated ALT and AST levels with a moderate vacuolation of hepatocytes (Table 1, Figs. 4a, b, 10b), which is consistent with a previous study [94], and could be correlated to LTZ effect. The later findings could interfere with getting a precise conclusion about AR hepatotoxic effect. The mechanism for explaining AR suspected hepatotoxicity is still unclear, nevertheless, AR contains hepatotoxic constituents such as salicylates and alkaloids, as well as, hepatoprotective triterpene glycosides [55].

One interesting finding from our study is that although vitamin C had a minor ovarian effect on PCOS, it had a protective effect when combined with AR which diminished the AR induced mild liver deterioration. The protective effect of vitamin C may be due to its scavenging effect on oxidant molecules that consequentially enhances the antioxidant capacity.

## Conclusion

The present study demonstrated the ability of *Actaea racemosa* and vitamin C combination to mitigate the reproductive and metabolic disorders of PCOS. The AR arsenal of secondary metabolites inhibited the androgen aromatization in the letrozole-induced PCOS rats, offsetting ovarian oxidative stress, which may be involved in the pathogenesis of PCOS, with enhanced hormonal profile, lipid profile, glucose level and liver functions. Furthermore, AR or its vitamin C combination increased Ki-67 expression in the granulosa cell layer along with a significant downregulation of Cyp19-α1 mRNA expression level. Our results of steroidogenesis modulation with a declined risk of hepatic adverse effects by AR and vitamin C combination warrant further studies of this combination in polycystic ovary syndrome.

## Supplementary Information

**Additional file 1: Table S1.** Metabolomic profiling of *Actaea racemosa* using UPLC–MS^n^, List of the identified metabolites in negative mode; structures, MS^n^ ions and/or fragmentation tree (**Figures S1–S12**) and the identified metabolites in positive mode; structures, MS^n^ ions and/or fragmentation tree (**Figures S13–S43**).

## Data Availability

The datasets supporting the conclusion of this article are included within the article and its additional files.

## Abbreviations

ALT: Alanine aminotransferase
AR: *Actaea racemose*
AST: Aspartate amino transferase
CMC: Carboxy methyl cellulose
CV: Coefficient of variation
GNPS: Global Natural Products Social Molecular Networking
GnRH: Gonadotropin-releasing hormone
GSH: Glutathione
HDL: High-density lipoprotein
HDMS: High definition mass spectrometer
HMDB: Human metabolites database
IMS: Ion mobility spectroscopy
LDL: Low-density lipoprotein
LTZ: Letrozole
LH: Luteinizing hormone
MDA: Malondialdehyde
MONA: Massbank of North America
PCOS: Polycystic ovary syndrome
QC: Quality control
Vit. C: Vitamin C
VLDL: Very low-density lipoprotein
