# Supplementary material for: Modulation of steroidogenesis by Actaea racemosa and vitamin C combination, in letrozole induced polycystic ovarian syndrome rat model: promising activity without the risk of hepatic adverse effect
Source: Chin Med. 2021 Apr 29;16:36. doi: 10.1186/s13020-021-00444-z (PMC8086310; doi:10.1186/s13020-021-00444-z)

**Additional file:**

**Modulation of steroidogenesis *by Actaea racemosa* and vitamin C combination, in Letrozole induced polycystic ovarian syndrome rat model: Promising activity without the risk of hepatic adverse effect**

[**Table S1 Metabolomic profiling of *Actaea racemosa* using UPLC-MS^n^** 3](#_Toc39141413)

[**Identified metabolites in negative mode; structures, MS^n^ ions and/or fragmentation tree** 7](#_Toc39141414)

[Figure S1 Trigalloylglucose 7](#_Toc39141415)

[Figure S2 Pentagalloylglucose 8](#_Toc39141416)

[Figure S3 Tetragaloylglucose 9](#_Toc39141417)

[Figure S4 hydrolysable ellagitannin (Rugosin E) 10](#_Toc39141418)

[Figure S5 protocatechuic acid/3,4-Dihydroxybenzoic acid 11](#_Toc39141419)

[Figure S6 Unidentified Triterpene 12](#_Toc39141420)

[Figure S7 Dihydroxydocosapentaenoic acid 13](#_Toc39141421)

[Figure S8 9,12,13-trihydroxyoctadec-10-enoic acid 14](#_Toc39141422)

[Figure S9 Quercitin-3-O-hexoside 15](#_Toc39141423)

[Figure S10 Kaempferol 3-O-(Rhamose-hexoside) 15](#_Toc39141424)

[Figure S11 Nerolidol 3-O-[dirhamnosyl-hexoside] 16](#_Toc39141425)

[Figure S12 Maltotriose 16](#_Toc39141426)

[**Identified metabolites in positive mode; structures, MS^n^ ions and/or fragmentation tree** 17](#_Toc39141427)

[Figure S13 Apigenin-6-C-hexoside-8-C pentoside 17](#_Toc39141428)

[Figure S14 Trihydroxy,methoxy-flavone-hexoside 18](#_Toc39141429)

[Figure S15 Undefined Ellagitannin-1 19](#_Toc39141430)

[Figure S16 Penta-hydroxy-flavone 21](#_Toc39141431)

[Figure S17 Kaempferide-3-rhamnoside 21](#_Toc39141432)

[Figure S18 Kaempferol 22](#_Toc39141433)

[Figure S19 Pentahydroxy-flavanonol 22](#_Toc39141434)

[Figure S20 Quercetin 23](#_Toc39141435)

[Figure S21 12-Oxophytodienoic acid 24](#_Toc39141436)

[Figure S22 Trihydroxy-dimethoxyflavone (3,7 dimethylquercetin) 24](#_Toc39141437)

[Figure S23 Trihydoxy-trimethoxy-flavone (dihydroxy-2-(hydroxy-methoxyphenyl)-dimethoxy-4H-chromen-4-one) 25](#_Toc39141438)

[Figure S24 (E,E)-Piperlonguminine 25](#_Toc39141439)

[Figure S25 3-Oxo-12,18-ursadien-28-oic acid 26](#_Toc39141440)

[Figure S26 Piperine 26](#_Toc39141441)

[Figure S27 LysoPC (18:3(6Z,9Z,12Z)) 27](#_Toc39141442)

[Figure S28 7-Hydroxy-3,4',8-trimethoxyflavone 27](#_Toc39141443)

[Figure S29 (+)-(5S,10R)-10,12-dihydroxy-7-oxo-20-norabieta-8,11,13-triene 28](#_Toc39141444)

[Figure S30 alpha-Irone 28](#_Toc39141445)

[Figure S31 LysoPC(16:0/0:0) 29](#_Toc39141446)

[Figure S32 LPC 18:1 29](#_Toc39141447)

[Figure S33 Undefined Triterpene 30](#_Toc39141448)

[Figure S34 2-Deoxycastasterone 31](#_Toc39141449)

[Figure S35 15,17,19-Nonacosatriynoic acid 31](#_Toc39141450)

[Figure S36 4α-formyl-4β-methyl-5α-cholesta-8,24-dien-3β-ol 32](#_Toc39141451)

[Figure S37 LPC 18:0 32](#_Toc39141452)

[Figure S38 MG(0:0/18:3(9Z,12Z,15Z)/0:0) 33](#_Toc39141453)

[Figure S39 (2β,3β,5β,9ξ,22R)-2,3,14,20,22,26-Hexahydroxycholest-7-en-6-one 33](#_Toc39141454)

[Figure S40 5-Alpha-Pregnane-3-Beta-Ol-Hemisuccinate 33](#_Toc39141455)

[Figure S41 (2E,4E,12E)-13-(1,3-benzodioxol-5-yl)-N-(2-methylpropyl)trideca-2,4,12-trienamide 34](#_Toc39141456)

[Figure S42 Dihydroxy-dimethoxyflavone (Crisimartin) 34](#_Toc39141457)

[Figure S43 13S-Hydroxy-9Z,11E,15Z-octadecatrienoic acid 35](#_Toc39141458)

# **Table S1 Metabolomic profiling of *Actaea racemosa* using UPLC-MS^n^**

| **RT (min)** | **m/z (Da)** | **CCS (Å^2^)** | **Adducts** | **Formula** | **Putative identification** | **Mass Error (ppm)** | **Chemical**  **Class** |
| --- | --- | --- | --- | --- | --- | --- | --- |
| 0.89 | 248.1132 | 152.4908 | M+H-H2O, M+H, M+K | C10H19NO7 | D-1-[(3-Carboxypropyl) amino]-1-deoxyfructose | 1.11 | Carbohydrate |
| 2.25 | 813.0500 | 410.5807 | - | C27H24O29 | Unknown | - | - |
| 2.48 | 565.1554 | 231.4041 | M+H-H2O, M+H, M+Na | C26H28O14 | Apigenin-6-C-hexoside-8-C pentoside | 0.53 | Flavonoids |
| 2.58 | 445.1130 | 204.5222 | M+H-H2O, M+H | C22H22O11 | Trihydroxy-methoxy-flavone-hexoside | 0.16 | Flavonoids |
| 2.60 | 319.0458 | 167.1482 | M+NH4, 2M+H | - | Unknown | - | - |
| 2.62 | 1087.0903 | 308.0089 | M+H-H2O, M+H, M+NH4, M+Na | C48H30O30 | Undefined Ellagitannin | - | Tannins |
| 2.62 | 917.0678 | 270.8768 | M+H-H2O | C41H26O26 | Undefined Ellagitannin | - | Tannins |
| 2.78 | 303.0513 | 162.0388 | M+H-H2O, M+H | C15H10O7 | Penta-hydroxyflavone | 2.30 | Flavonoids |
| 2.80 | 479.0835 | 202.3183 | M+Na, M+K | - | Unknown | - |  |
| 2.81 | 429.1190 | 199.4060 | M+H-H2O, M+H | C22H22O10 | Kaempferide-3-rhamnoside | 2.12 | Flavonoids |
| 2.95 | 287.0556 | 158.7635 | M+H, 2M+H | C15H10O6 | kaempferol | 2.18 | Flavonoids |
| 3.00 | 463.0871 | 198.9574 | M+H, M+Na, M+ACN+Na | C21H18O12 | Kaempferol 3-hexouronide | 0.02 | Flavonoids |
| 3.68 | 287.0556 | 158.7635 | M+H-H2O | C15H12O7 | Pentahydroxy-flavanonol | 1.76 | Flavonoids |
| 3.70 | 303.0508 | 162.0388 | M+H | C15H10O7 | Quercetin | 2.92 | Flavonoids |
| 4.07 | 275.2007 | 166.3909 | M+H-H2O, M+2Na-H | C18H28O3 | 12-Oxophytodienoic acid | 0.45 | Fatty acid/ Lipids |
| 4.29 | 331.0821 | 170.5284 | M+H | C17H14O7 | trihydroxy-dimethoxyflavone (3,7 dimethylquercetin) | 2.58 | Flavonoids |
| 4.47 | 361.0927 | 177.1917 | M+H, M+Na | C18H16O8 | Trihydoxy-trimethoxy-flavone (dihydroxy-2-(hydroxy-methoxyphenyl)-dimethoxy-4H-chromen-4-one) | 2.42 | Flavonoids |
| 4.77 | 315.0874 | 167.2329 | M+H | C17H14O6 | Dihydroxy-dimethoxyflavone (Crisimartin) | 3.20 | Flavonoids |
| 5.17 | 375.1077 | 182.3495 | M+H, M+Na, M+2Na-H | - | Unknown | - | - |
| 5.24 | 274.1451 | 171.9042 | M+H-H2O, M+H | C16H19NO3 | Piperlonguminine | 1.47 | Alkaloids |
| 5.41 | 453.3365 | 213.2860 | M+H-H2O, M+H | C30H44O3 | 3-Oxo-12,18-ursadien-28-oic acid | -0.47 | Triterpene |
| 5.57 | 286.1415 | 169.7464 | M+H-H2O, M+H | C17H19NO3 | Piperine | 1.78 | Alkaloids |
| 6.03 | 518.3246 | 228.3775 | M+H-H2O, M+H, M+Na, 2M+H | C26H48NO7P | LysoPC(18:3(6Z,9Z,12Z)) | 0.93 | Fatty acid/ Lipids |
| 6.19 | 329.1027 | 170.5684 | M+H, M+Na | C18H16O6 | 7-Hydroxy-3,4',8-trimethoxyflavone | 2.23 | Flavonoids |
| 6.30 | 314.1747 | 176.3290 | M+H, M+Na | C19H23NO3 | Unknown | - | - |
| 6.61 | 285.1854 | 169.7718 | M+H-H2O | C19H26O3 | (+)-(5S,10R)-10,12-dihydroxy-7-oxo-20-norabieta-8,11,13-triene | 1.67 | Diterpenes |
| 6.61 | 331.1891 | 175.9578 | M+H-H2O, M+K | C20H28O5 | diterpenoid (like sinularolide C) | -3.77 | Diterpenes |
| 6.66 | 277.2167 | 169.9899 | M+H-H2O | C16H23N2O | 13S-Hydroxy-9Z,11E,15Z-octadecatrienoic acid | 3.63 | Fatty acid/ Lipids |
| 6.70 | 520.3410 | 235.3910 | M+Na, M+2Na-H, M+2Na | C26H51N1O7P1 | PC(18:2/0:0) | - | Fatty acid/ Lipids |
| 6.82 | 224.2019 | 162.5813 | M+NH4 | C14H22O | alpha-Irone | 4.91 | Miscellaneous terpenoids |
| 7.05 | 677.3717 | 261.8748 | M+H-H2O, M+Na, M+K | C35H60O7P2 | Heptaprenyl diphosphate | 1.57 | Miscellaneous terpenoid |
| 7.18 | 496.3406 | 235.6815 | M+H, M+Na, 2M+H, M+H-H2O | C24H50NO7P | LysoPC(16:0/0:0) | 1.64 | Fatty acid/ Lipids |
| 7.33 | 437.3413 | 206.4078 | M+H-H2O, M+H | C30H46O3 | Undefined Triterpene | -0.31 | Triterpenes |
| 7.58 | 437.3410 | 206.4078 | M+H-H2O, M+H | C30H46O3 | Undefined Triterpene | 0.98 | Triterpenes |
| 7.59 | 522.3560 | 237.1251 | M+H-H2O, M+H, M+K, 2M+H | C26H52NO7P | LPC 18:1 | -13.12 | Fatty acid/ Lipids |
| 7.71 | 471.3441 | 213.0488 | M+Na | C28H48O4 | 2-Deoxycastasterone | -0.76 | Steroids |
| 7.83 | 409.3465 | 205.0505 | M+H-H2O, M+H | C29H46O2 | 15,17,19-Nonacosatriynoic acid | 0.06 | Fatty acid/ Lipids |
| 7.83 | 437.3416 | 208.1851 | M+H-H2O, M+H | C30H46O3 | Undefined Triterpene | 0.31 | Triterpenes |
| 7.97 | 437.3412 | 208.1851 | M+H-H2O, M+H, M+K | C30H46O3 | Undefined Triterpene | -0.48 | Triterpenes |
| 7.98 | 409.3466 | 205.0505 | M+H-H2O, M+H | C29H46O2 | 4α-formyl-4β-methyl-5α-cholesta-8,24-dien-3β-ol | 0.25 | Steroids |
| 8.74 | 524.3718 | 242.3696 | M+H, M+Na | C26H54NO7P | LPC 18:0 | 1.43 | Fatty acid/ Lipids |
| 8.92 | 375.2511 | 200.2811 | M+H-H2O, M+H, M+Na | C21H36O4 | MG (0:0/18:3(9Z,12Z,15Z)/0:0) | 2.58 | Fatty acid/ Lipids |
| 8.94 | 463.3032 | 213.1525 | M+H-H2O | C27H44O7 | (2β,3β,5β,9ξ,22R)-2,3,14,20,22,26-Hexahydroxycholest-7-en-6-one | -4.51 | Steroids |
| 8.95 | 419.2773 | 206.6752 | M+H | C25H38O5 | 5-Alpha-Pregnane-3-Beta-Ol-Hemisuccinate | -4.45 | Steroids |
| 8.95 | 507.3299 | 221.4533 | M+H, M+NH4, M+Na | C27H48O7 | Cholestane-heptol | -0.09 | Steroids |
| 9.12 | 384.2536 | 198.3296 | M+H | C24H33NO3 | (2E,4E,12E)-13-(1,3-benzodioxol-5-yl)-N-(2-methylpropyl)-trideca-2,4,12-trienamide [Guineensine] | 2.62 | Alkaloids |
| 11.15 | 621.3076 | 255.3745 | M+H, M+Na | C29H48O14 | Unknown | - | - |
| 1.07 | 503.1600 | 200.7911 | M-H2O-H, M-H, M+FA-H, 2M-H | C18H32O16 | Trihexoside (Maltotriose) | 3.94 | Carbohydrates |
| 2.20 | 860.0801 | 407.9614 | M-2H | C75H54O48 | Hydrolysable ellagitannins (like rugosin E) | -2.10 | Tannins |
| 2.25 | 784.0774 | 390.8312 | M-2H | C68H50O44 | Hydrolysable tannins (like Heterophylliin F) | 1.21 | Tannins |
| 2.30 | 785.0839 | 264.9880 | M-H | C34H26O22 | Tetragaloylglucose | -0.47 | Tannins |
| 2.32 | 860.5837 | 411.4385 | - | - | unknown | - | - |
| 2.34 | 1085.0727 | 297.7776 | M-H2O-H, M-H | C48H32O31 | Hydrolysable tannins (like Sanguiin H2) | -2.05 | Tannins |
| 2.40 | 859.0788 | 407.9652 | - | - | unknown | - | - |
| 2.40 | 709.0775 | 373.6648 | - | - | unknown | - | - |
| 2.42 | 635.0918 | 222.5784 | M-2H, M-H | C27H24O18 | tri-O-galloylglucose/Tannic acid | -1.22 | Tannins |
| 2.42 | 1176.1129 | 479.4972 | - | - | unknown | - | - |
| 2.46 | 563.1396 | 228.4460 | M+FA-H, 2M-H | - | unknown | - | - |
| 2.57 | 787.1000 | 275.2782 | M-H | C34H28O22 | Tetragaloylglucose | 0.02 | Tannins |
| 2.58 | 479.0805 | 199.2752 | - | - | unknown | - | - |
| 2.62 | 1085.0726 | 297.7776 | M+Na-2H, M+Cl, M+K-2H | - | unknown | - | - |
| 2.62 | 542.0350 | 328.7312 | M-2H | C48H30O30 | Hydrolysable ellagitannins (like Castacrenin D) | 2.17 | Tannins |
| 2.69 | 939.1069 | 294.9626 | M-2H, M-H | C41H32O26 | pentagalloylglucose | 2.06 | Tannins |
| 2.72 | 860.0818 | 414.9171 | - | - | unknown | - | - |
| 2.72 | 784.0771 | 397.8176 | - | - | unknown | - | - |
| 2.78 | 463.0833 | 197.6804 | M+Na-2H, M+Cl | - | unknown | - | - |
| 2.80 | 457.1204 | 190.5983 | M-H, M+Cl | - | unknown | - | - |
| 2.82 | 593.1487 | 228.1720 | M-H | C27H30O15 | Kaempferol 3-O-(Rhamose-hexoside) | -4.26 | Flavonoids |
| 2.87 | 300.9984 | 149.3356 | M+Na-2H, M+Cl, 2M-H | - | unknown | - | - |
| 2.95 | 447.0934 | 197.8819 | M-H | C21H20O11 | Quercitin-3-O-hexoside | 0.29 | - |
| 2.97 | 461.0733 | 195.9186 | M-H2O-H, M-H, 3M-H | C7H6O4 | protocatechuic acid/3,4-Dihydroxybenzoic acid | -1.09 | Phenolic acids |
| 4.09 | 327.2163 | 180.0495 | 2M+FA-H, 2M+Hac-H | - | Unknown | - | - |
| 4.29 | 329.2319 | 181.8274 | M-H2O-H, M-H, M+Na-2H | C18H34O5 | 9,12,13-trihydroxyoctadec-10-enoic acid | -4.47 | Fatty acid/ Lipids |
| 5.41 | 487.3405 | 222.2344 | M-H, M+FA-H, 2M-H | C30H48O5 | Undefined Triterpene | -4.93 | Triterpenes |
| 5.86 | 487.3392 | 222.2344 | M-H | C30H48O5 | Undefined Triterpene | -2.68 | Triterpenes |
| 5.91 | 721.3643 | 267.1128 | M-H, M+Cl, M+FA-H | C33H56O14 | Nerolidol 3-O-[dirhamnosyl-hexoside] | -2.15 | Miscellaneous terpenoids |
| 6.61 | 285.1865 | 173.7475 | M-H2O-H | C19H28O3 | 11beta,17beta-Dihydroxy-4-androsten-3-one | 1.56 | Steroids |
| 6.65 | 293.2090 | 175.3713 | M+Na-2H | C16H32O3 | 6-Hydroxyhexadecanoic acid | -2.95 | Fatty acid/ Lipids |
| 7.04 | 699.3800 | 265.5467 | - | - | unknown | - | - |
| 7.58 | 471.3462 | 218.9031 | M-H, 2M-H, 2M+FA-H | - | unknown | - | - |
| 7.80 | 471.3430 | 220.6718 | M-H, M+FA-H | C30H48O4 | Undefined Triterpene | -0.07 | Triterpene |
| 7.97 | 471.3471 | 218.9031 | M-H, M+FA-H | C30H48O4 | Undefined Triterpene | -1.78 | Triterpenes |
| 8.17 | 577.2682 | 231.8176 | M-H, M+FA-H, 2M-H | - | unknown | - | - |
| 8.35 | 358.2103 | 188.4969 | - | - | unknown | - | - |
| 8.60 | 345.2067 | 185.1287 | M-H | C21H30O4 | unknown | -1.19 | - |
| 8.81 | 383.2241 | 202.4412 | M-H, M+Na-2H, M+K-2H | C22H34O4 | Di-hydroxy-docosapentaenoic acid | -2.49 | Fatty acid/ Lipids |

# **Identified metabolites in negative mode; structures, MS^n^ ions and/or fragmentation tree**

## Figure S1 Trigalloylglucose


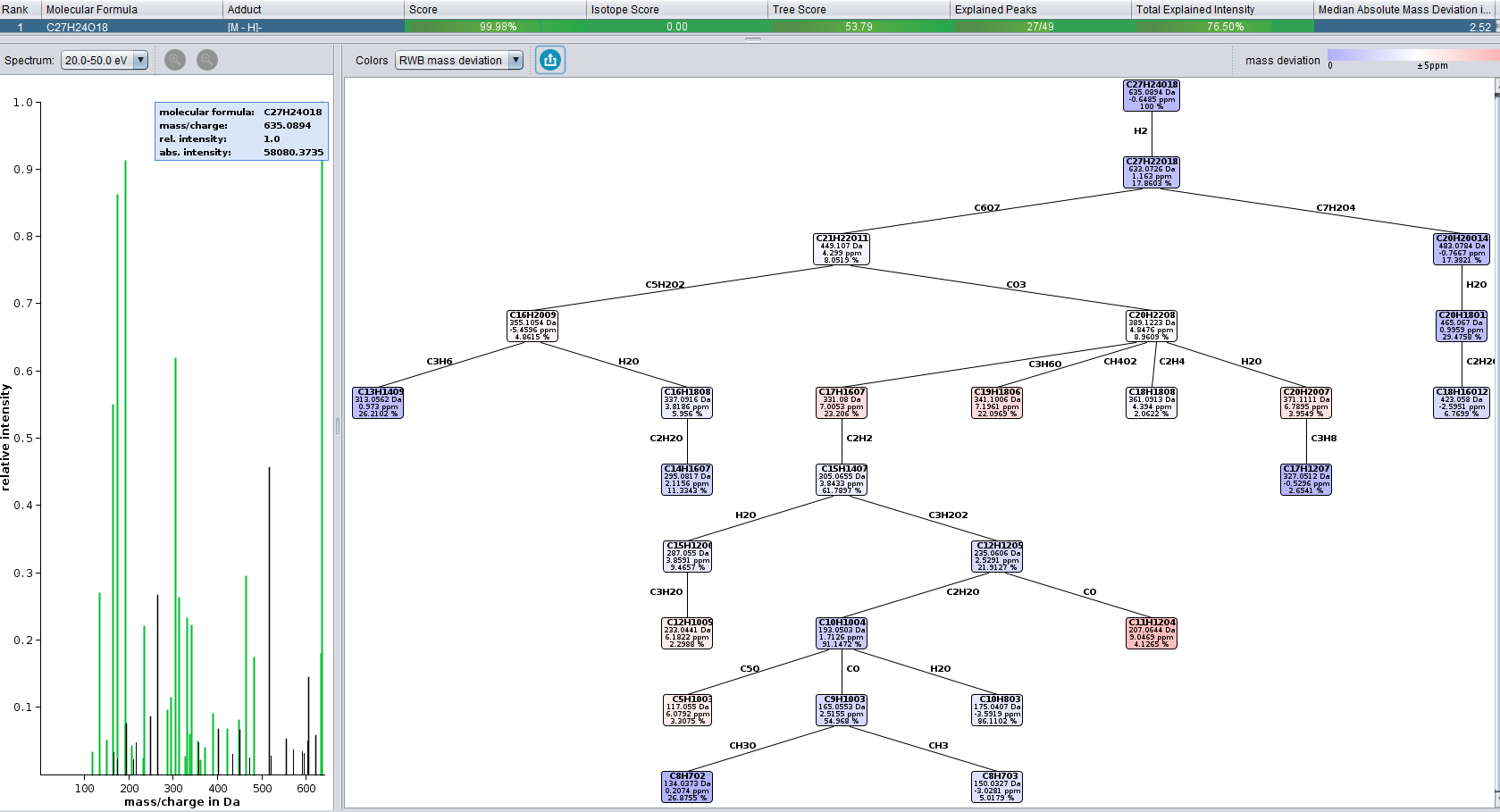

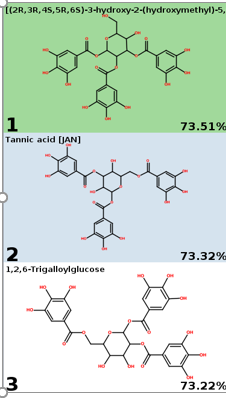


Matched fragments Unmatched fragments

## Figure S2 Pentagalloylglucose


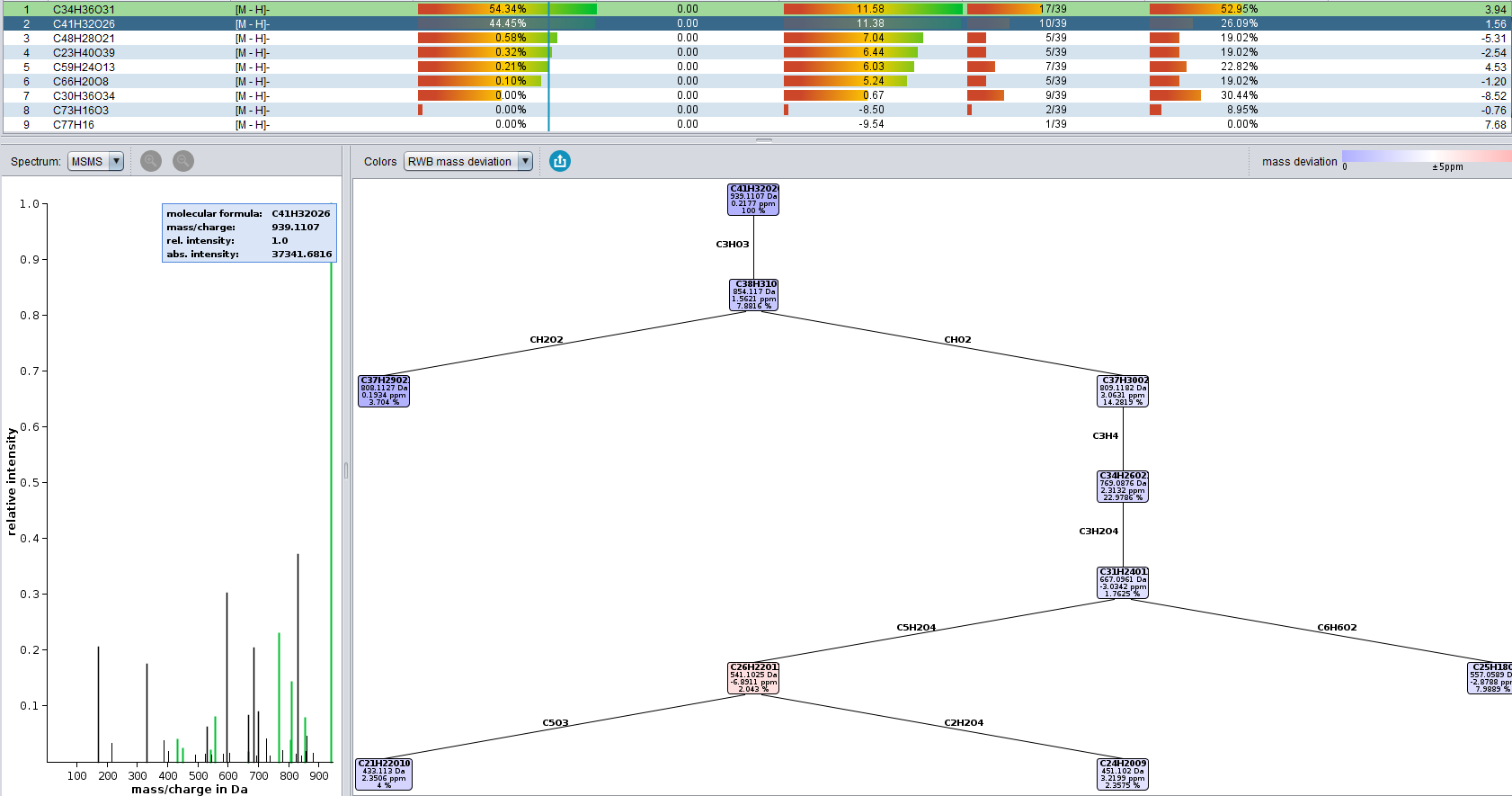

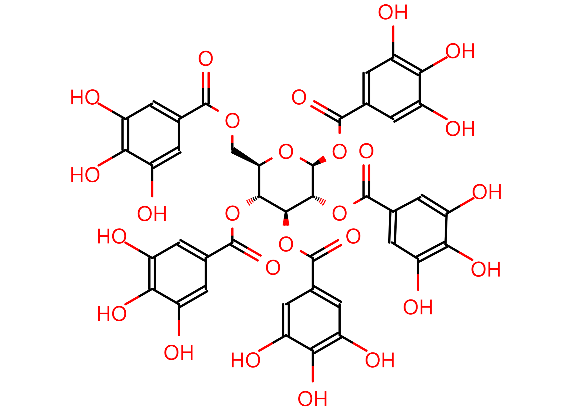


## Figure S3 Tetragaloylglucose


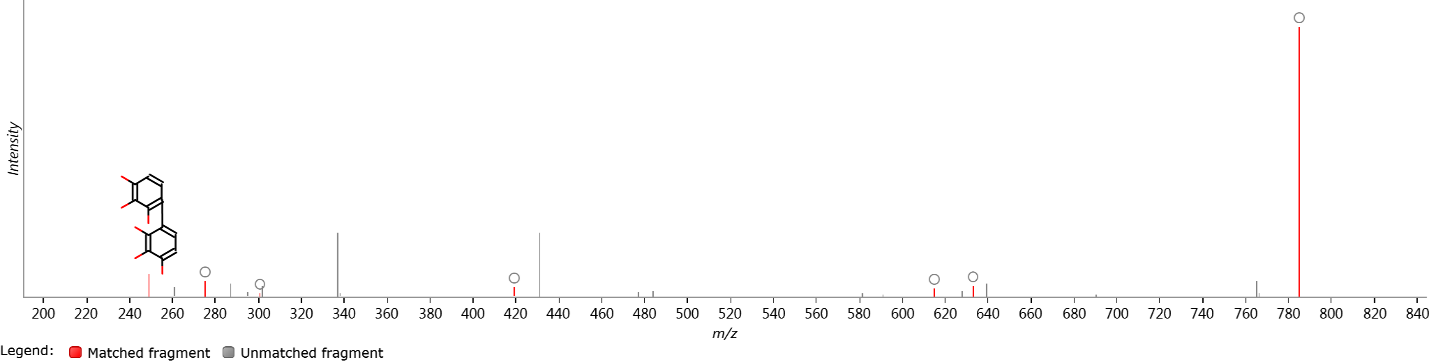

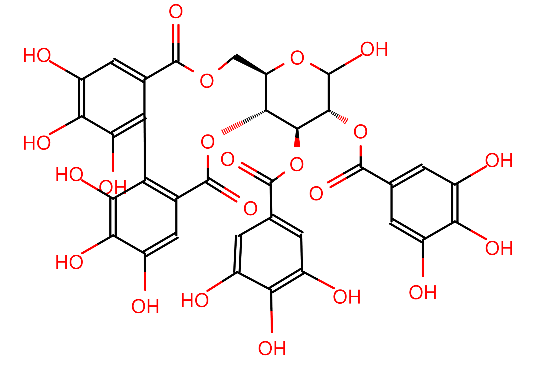

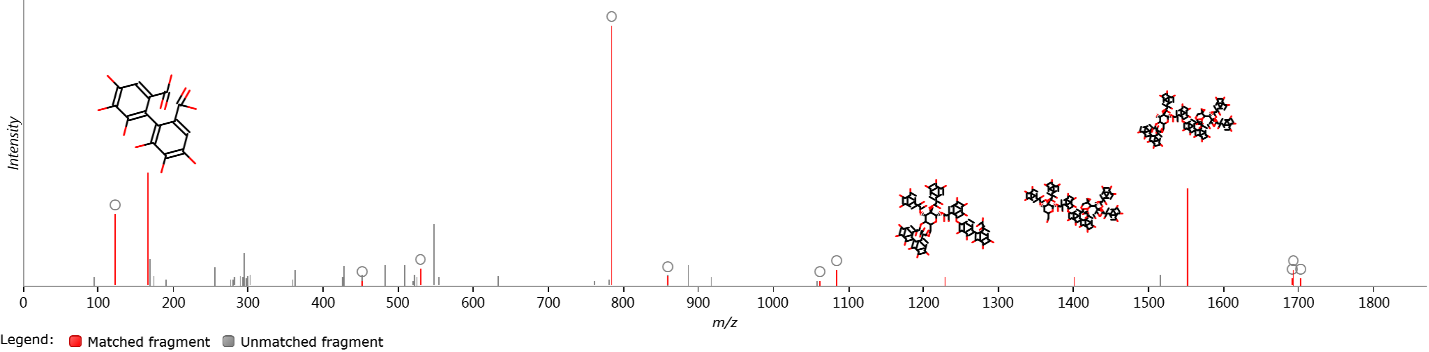

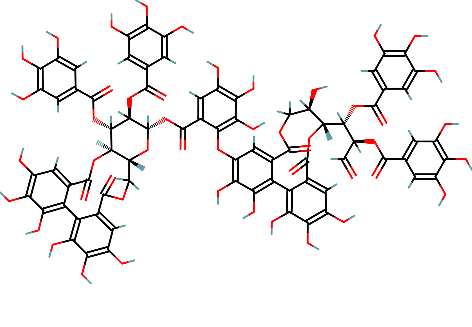


## Figure S4 hydrolysable ellagitannin (Rugosin E)

## Figure S5 protocatechuic acid/3,4-Dihydroxybenzoic acid


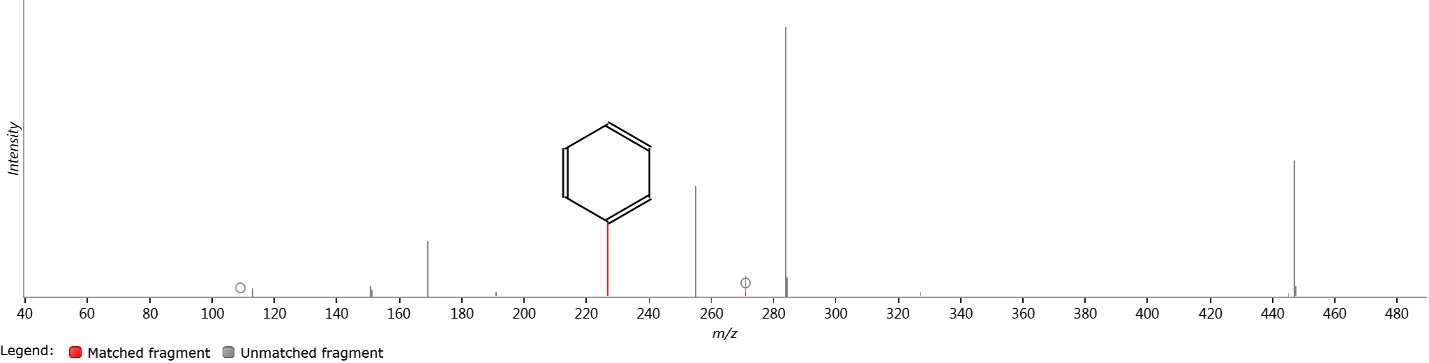

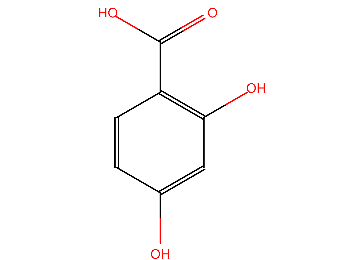


## Figure S6 Unidentified Triterpene


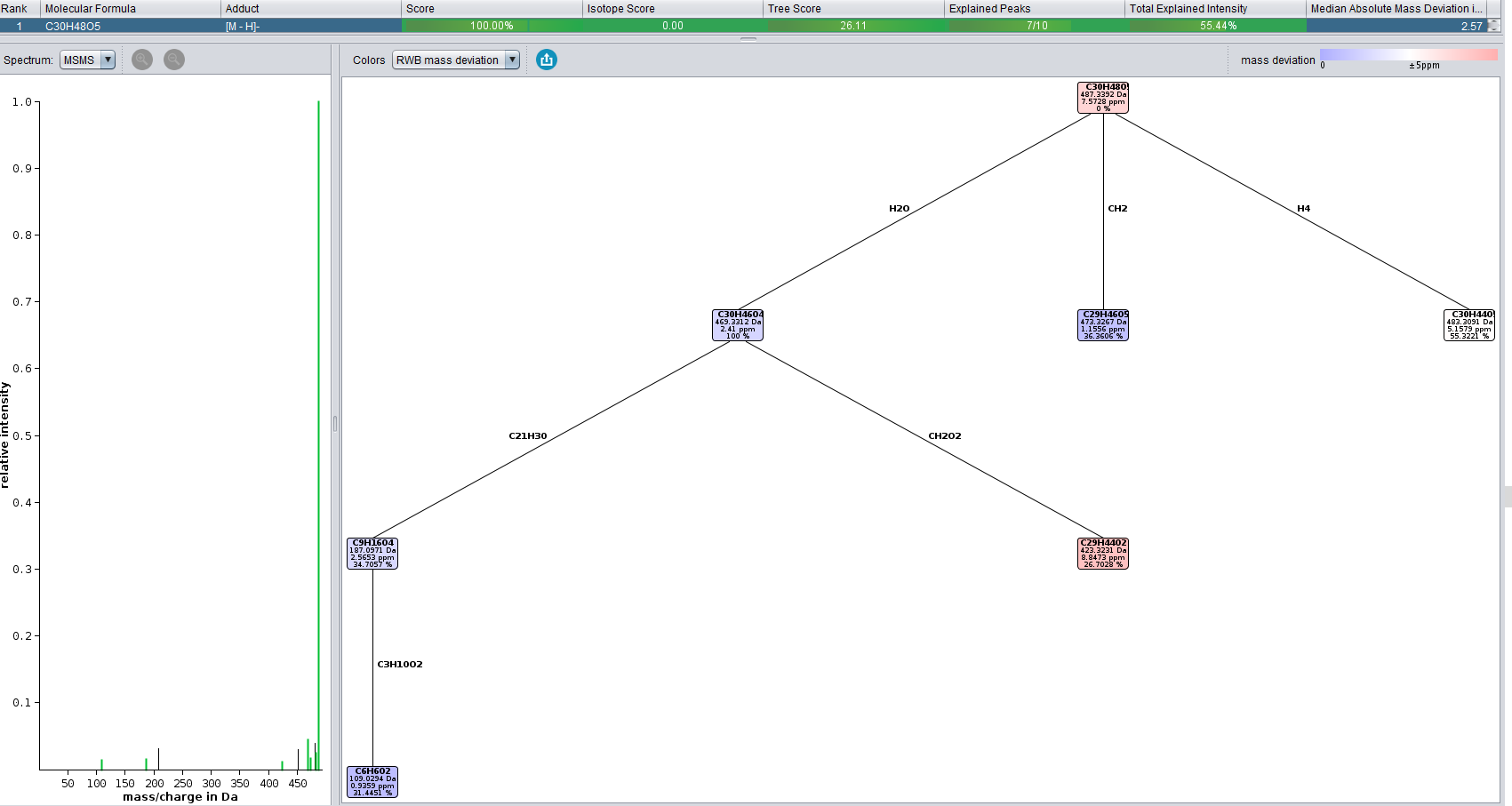

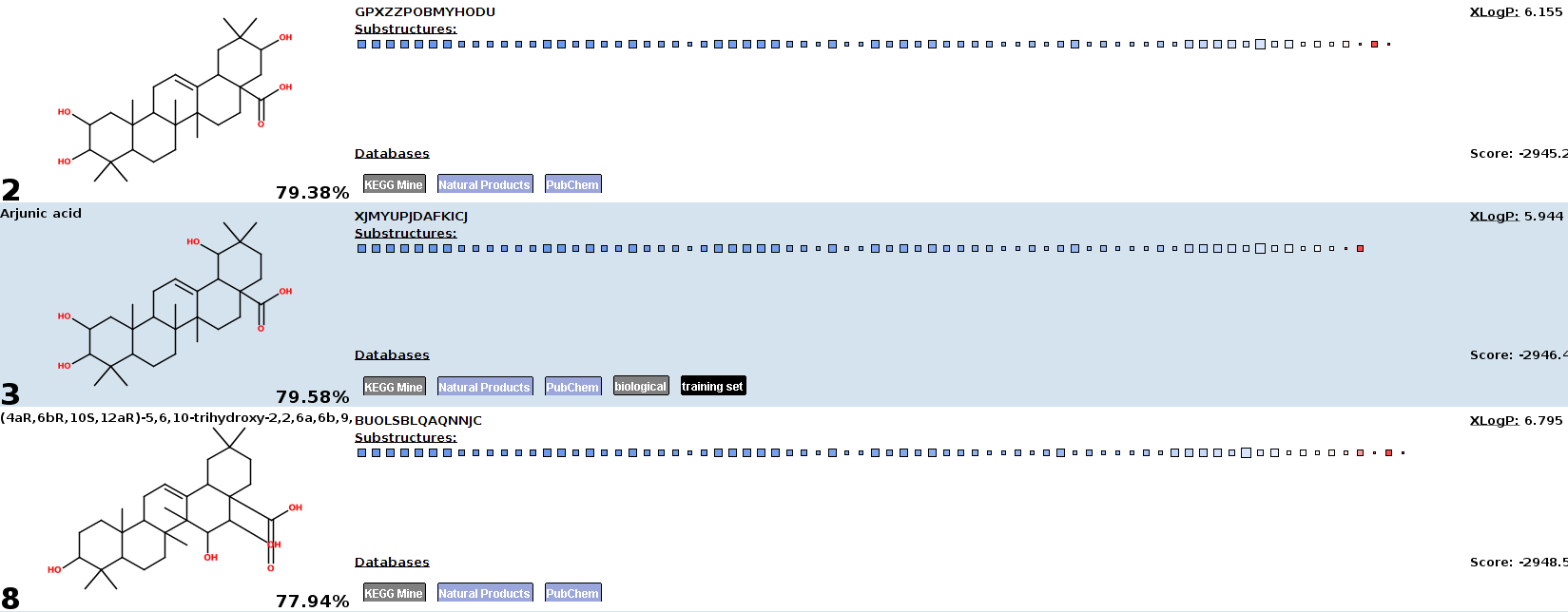


## Figure S7 Dihydroxydocosapentaenoic acid


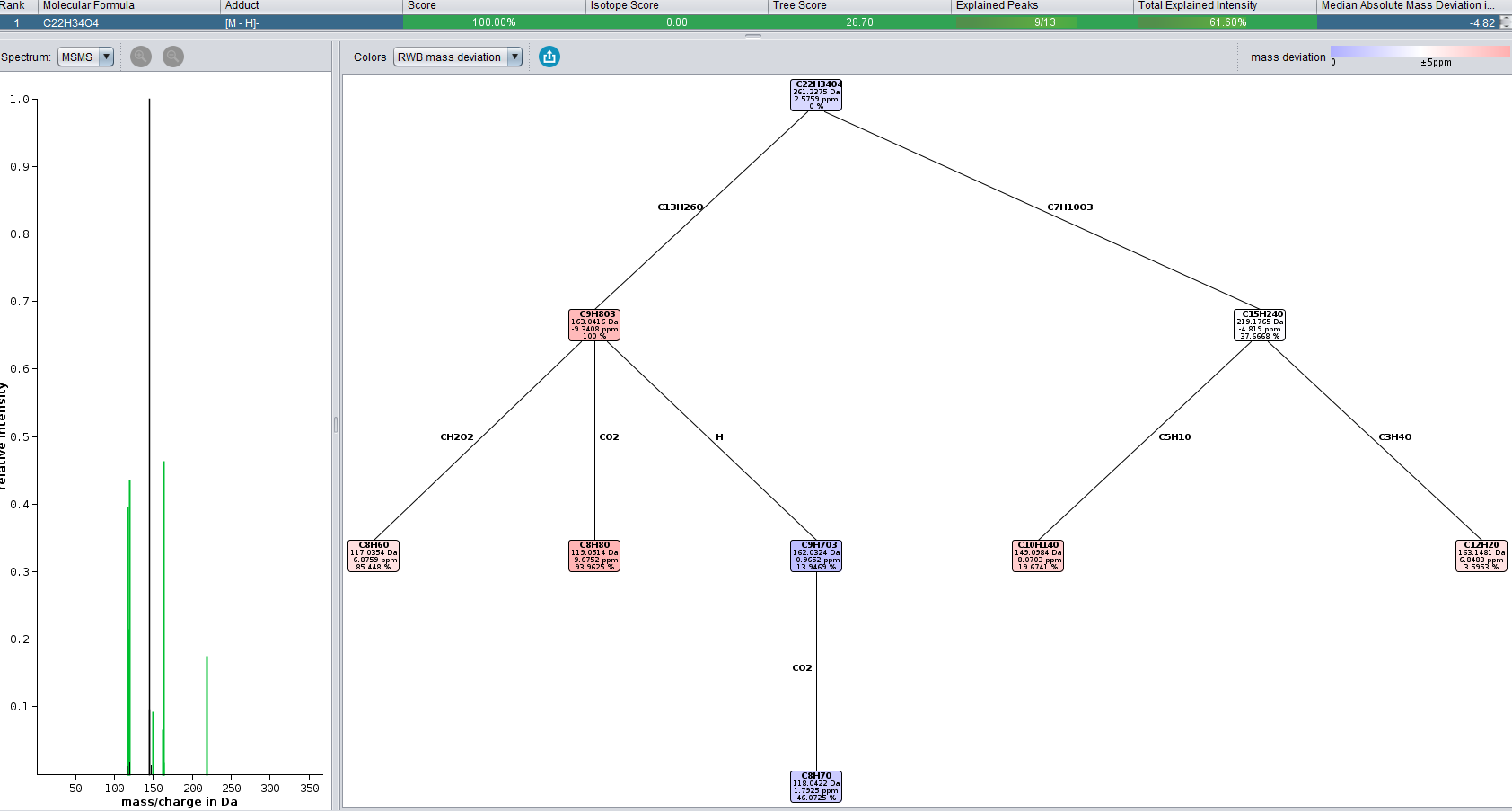

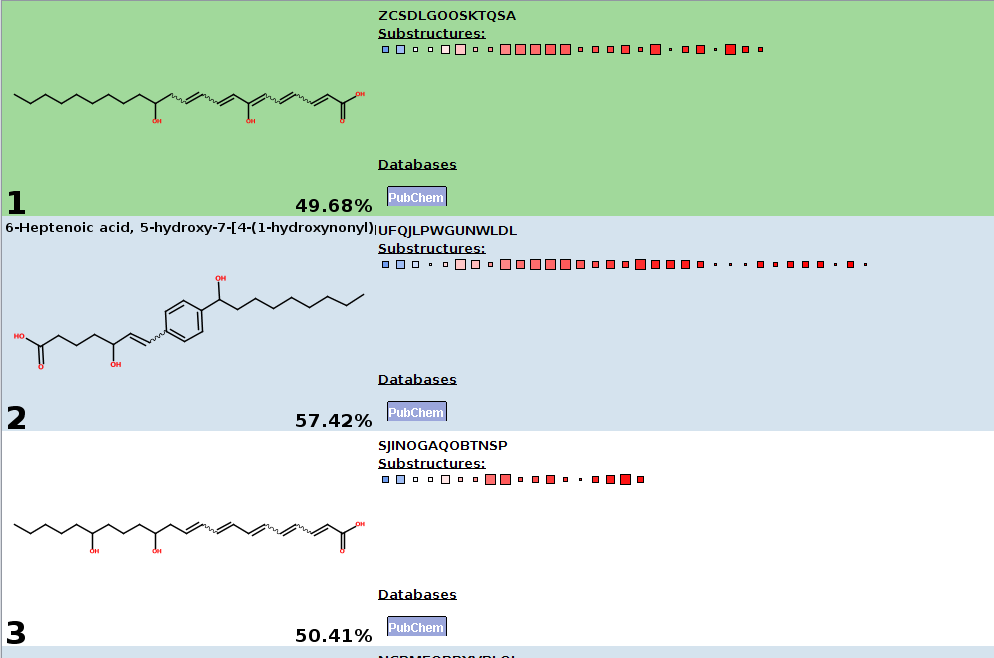

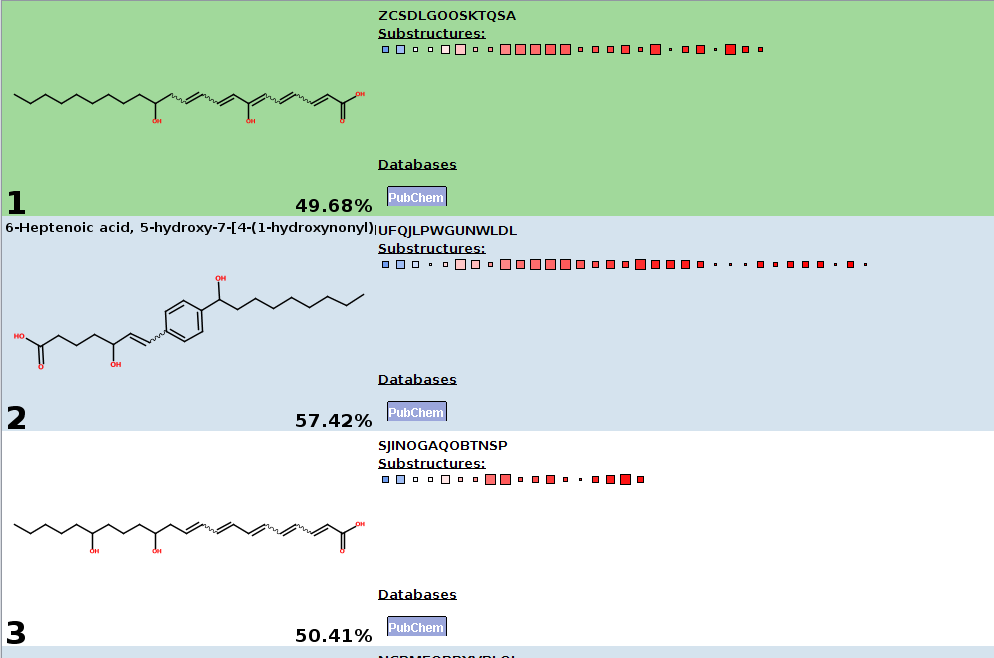

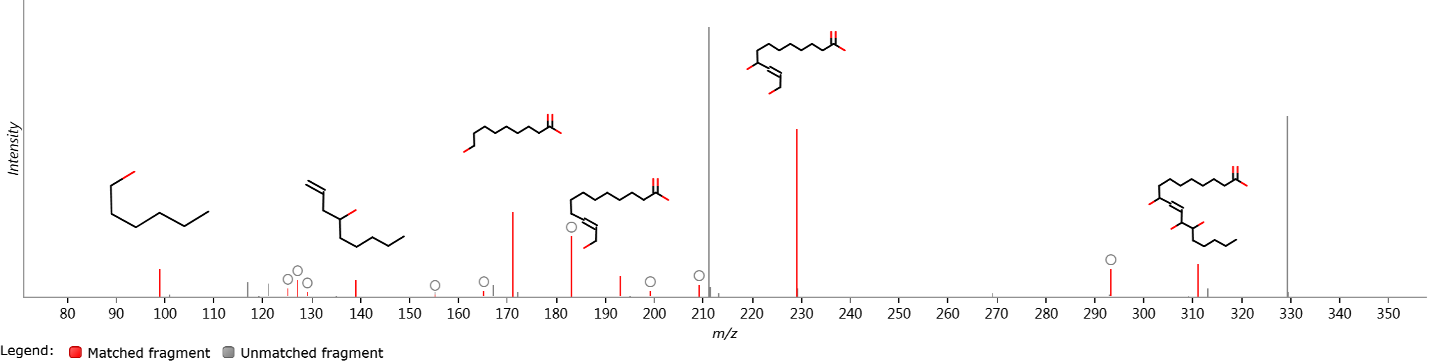

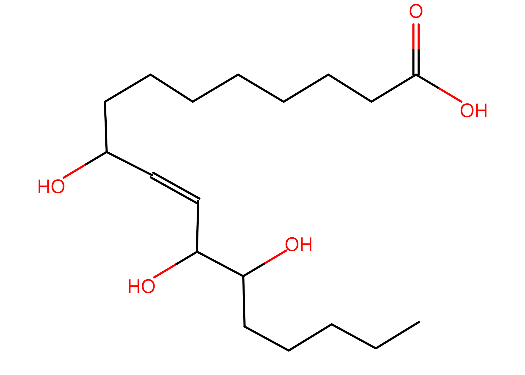


## Figure S8 9,12,13-trihydroxyoctadec-10-enoic acid

## Figure S9 Quercitin-3-O-hexoside


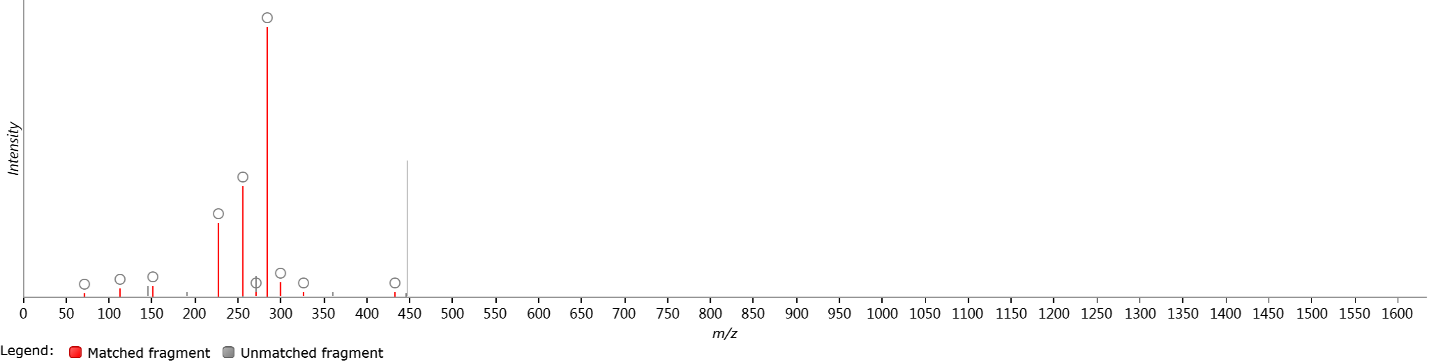

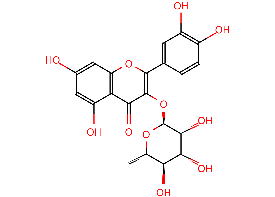


## Figure S10 Kaempferol 3-O-(Rhamose-hexoside)


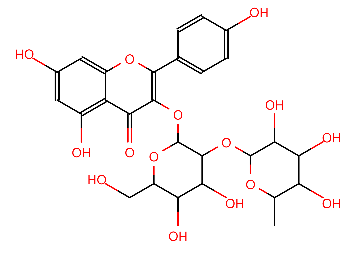

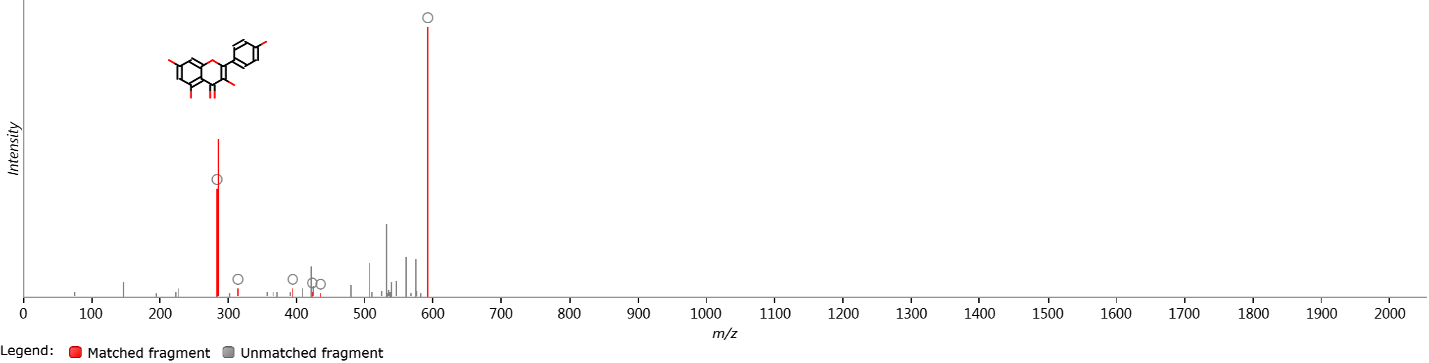


## Figure S11 Nerolidol 3-O-[dirhamnosyl-hexoside]


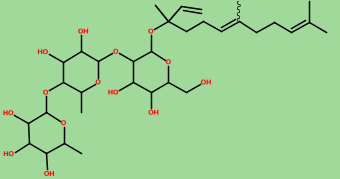

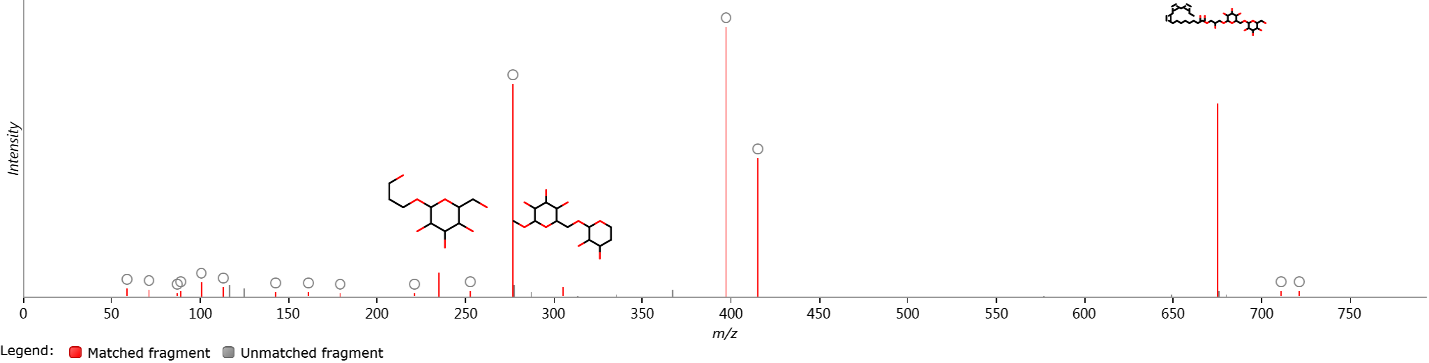


## Figure S12 Maltotriose


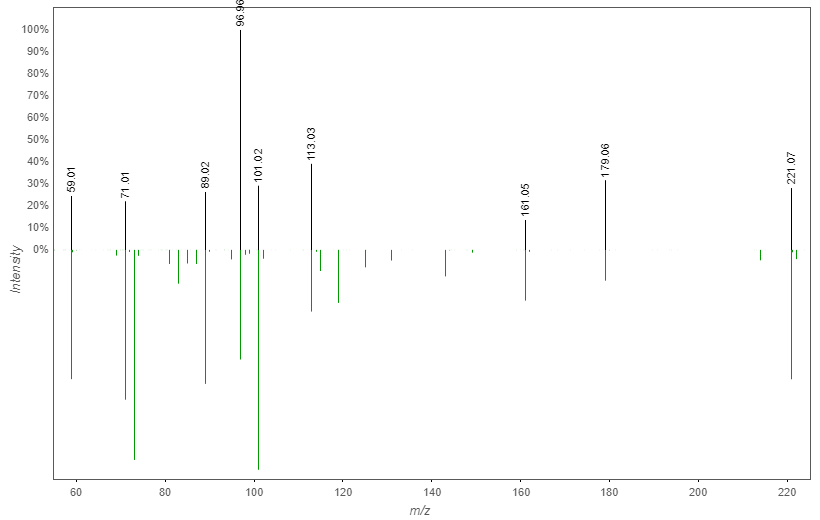

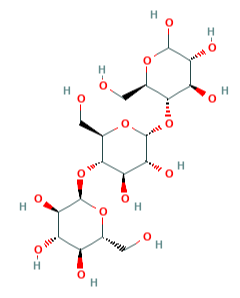


GNPS library

Measured

# **Identified metabolites in positive mode; structures, MS^n^ ions and/or fragmentation tree**

##
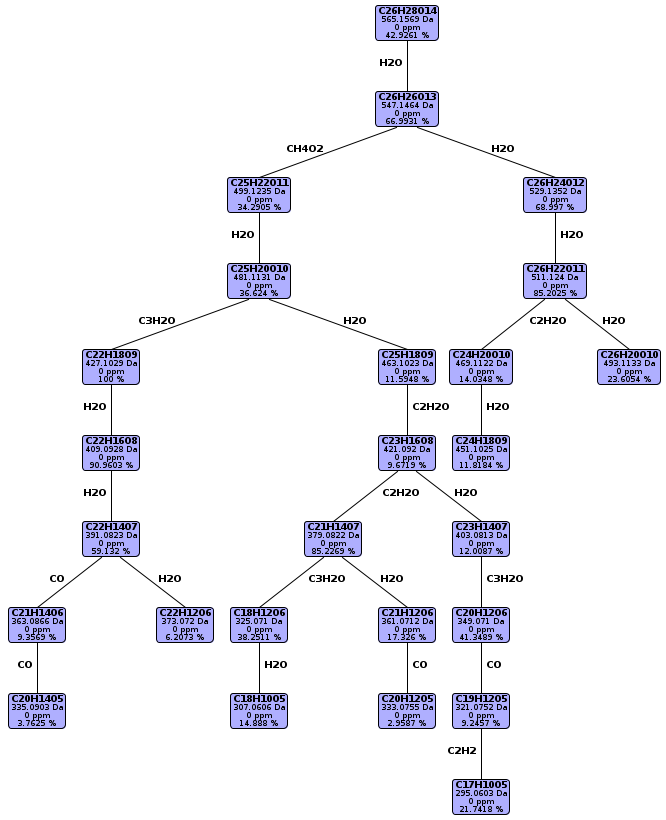
Figure S13 Apigenin-6-C-hexoside-8-C pentoside


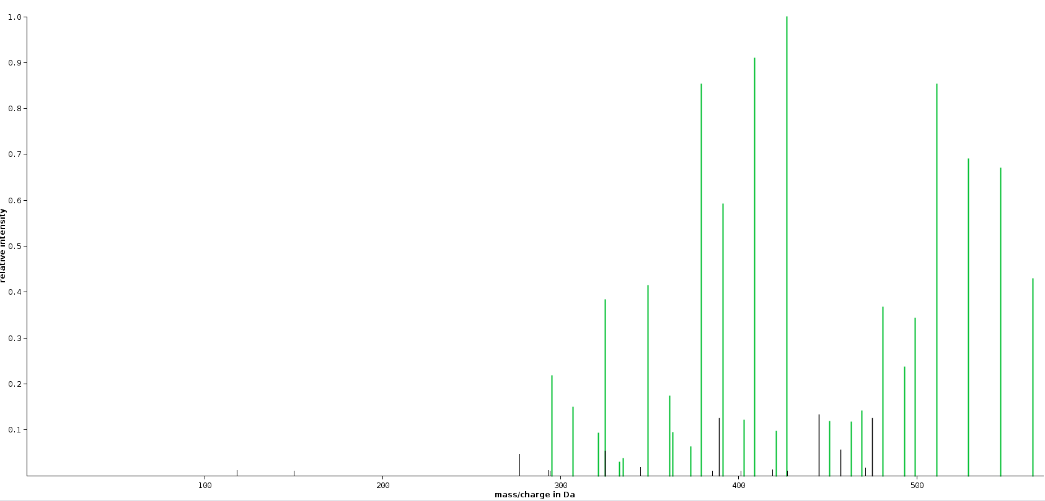

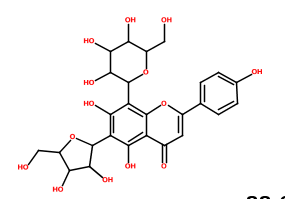


## Figure S14 Trihydroxy,methoxy-flavone-hexoside


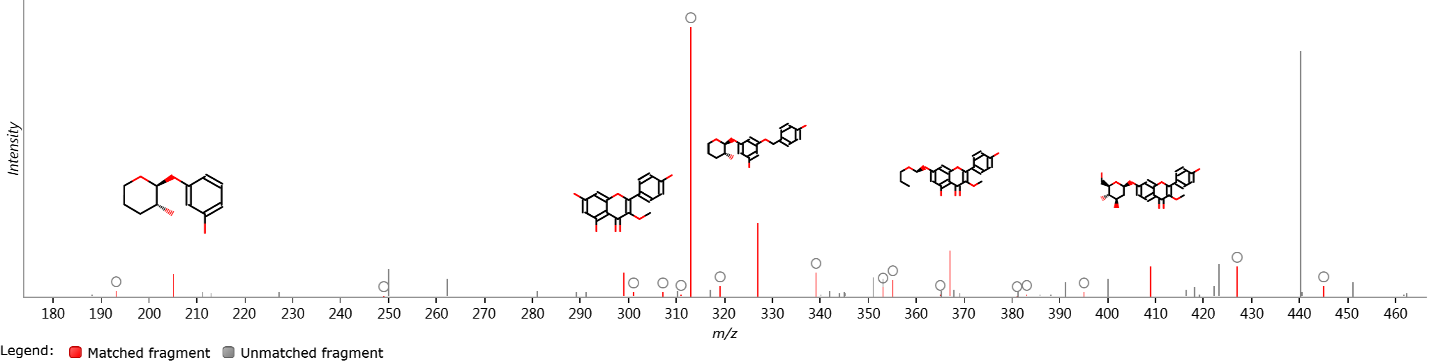

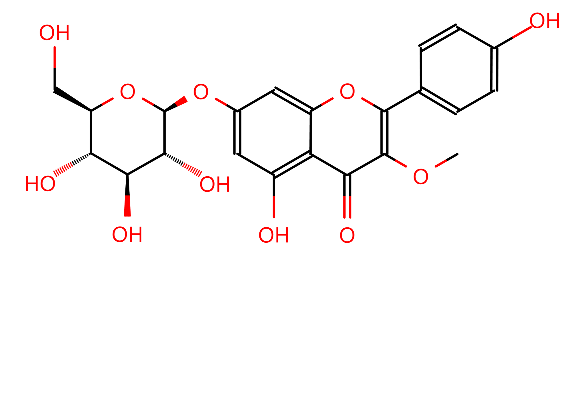


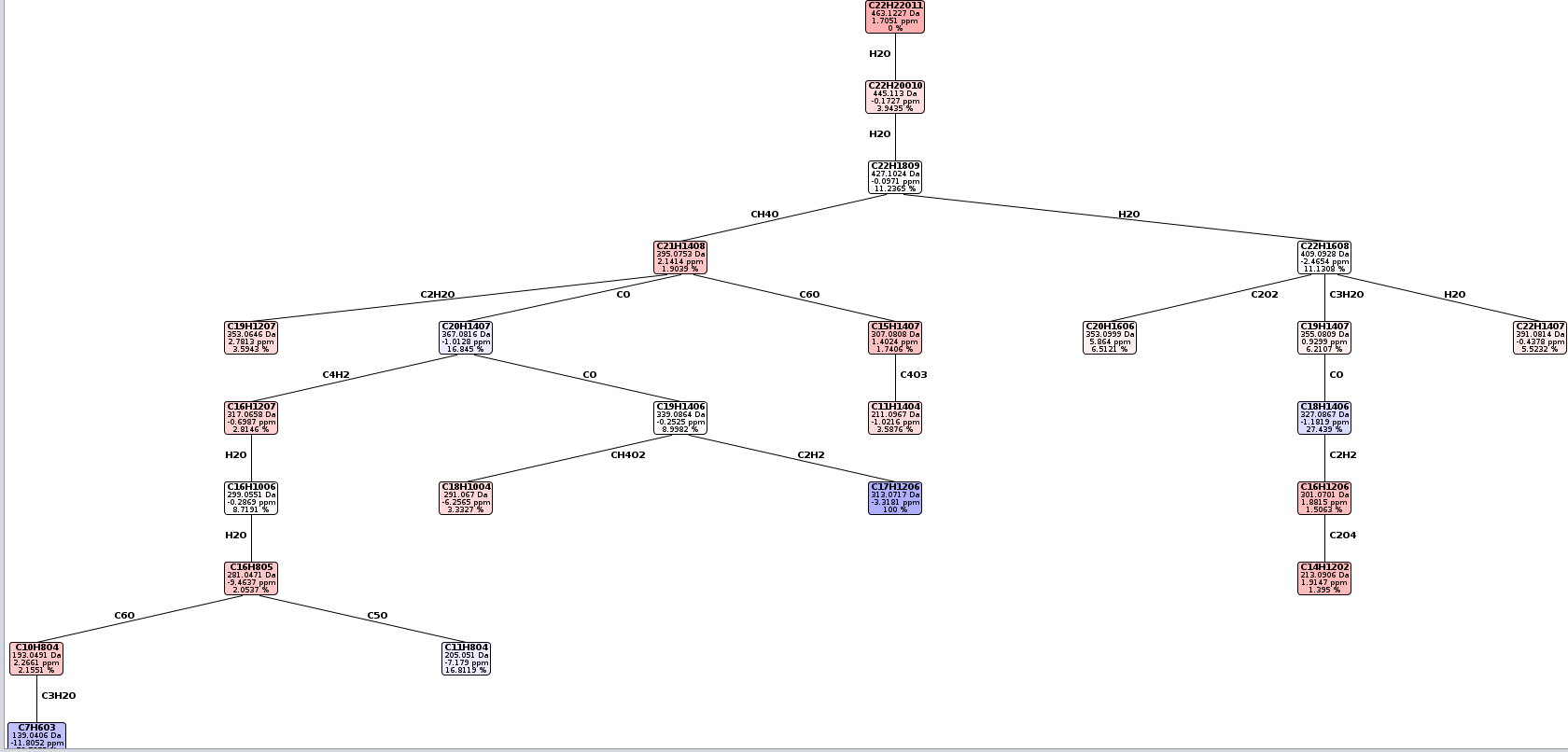


##
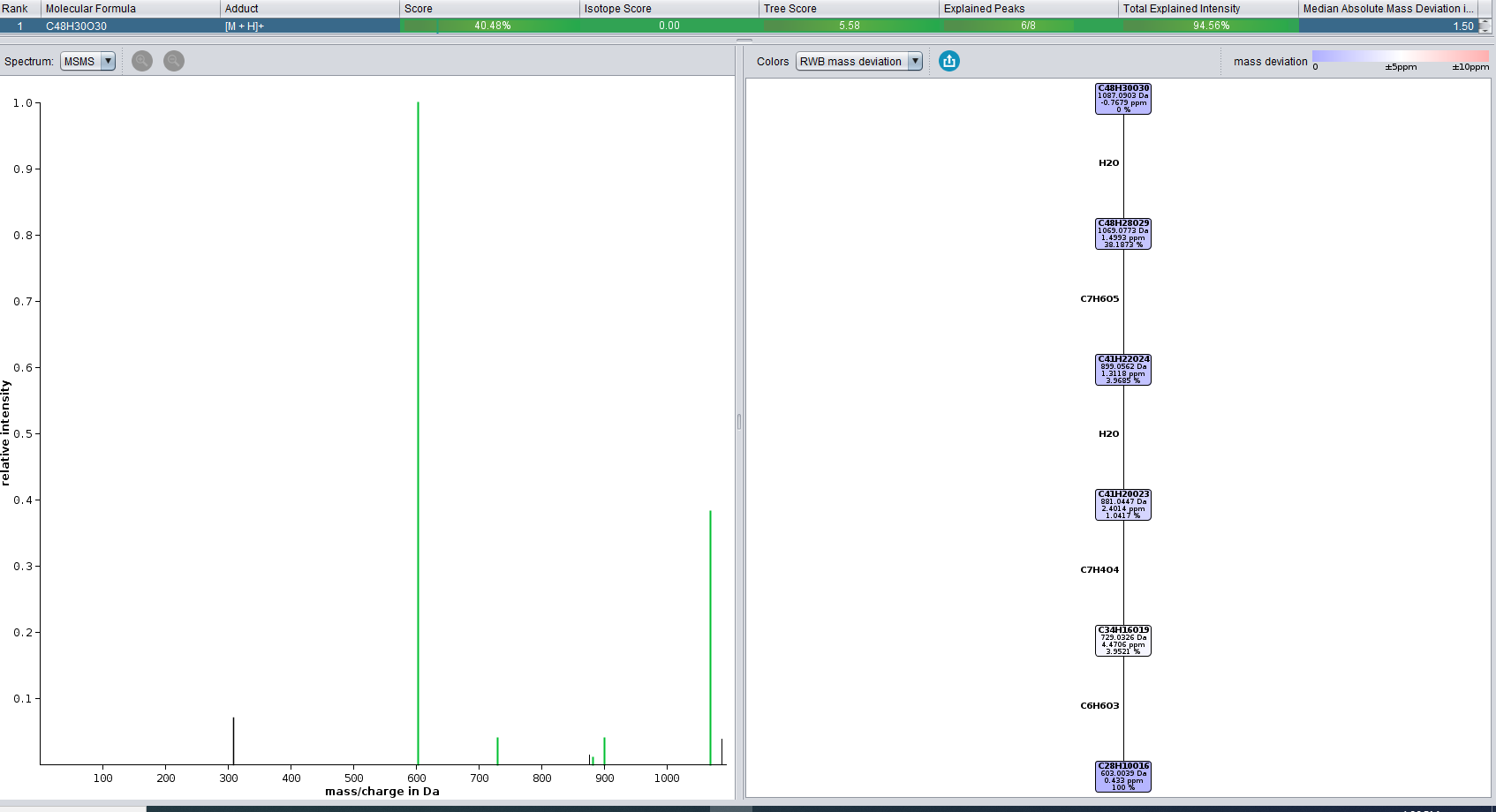
Figure S15 Undefined Ellagitannin-1


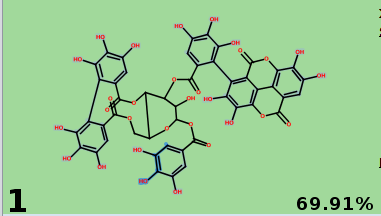

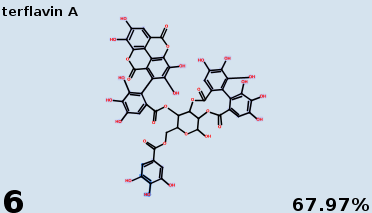


Undefined Ellagitannin-2


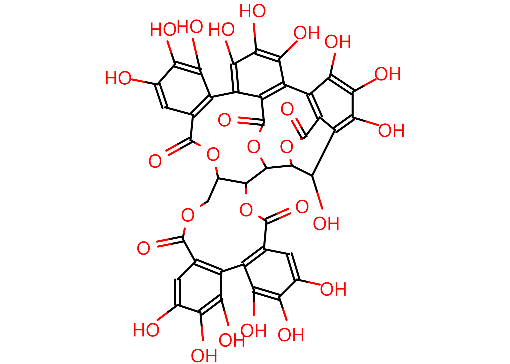

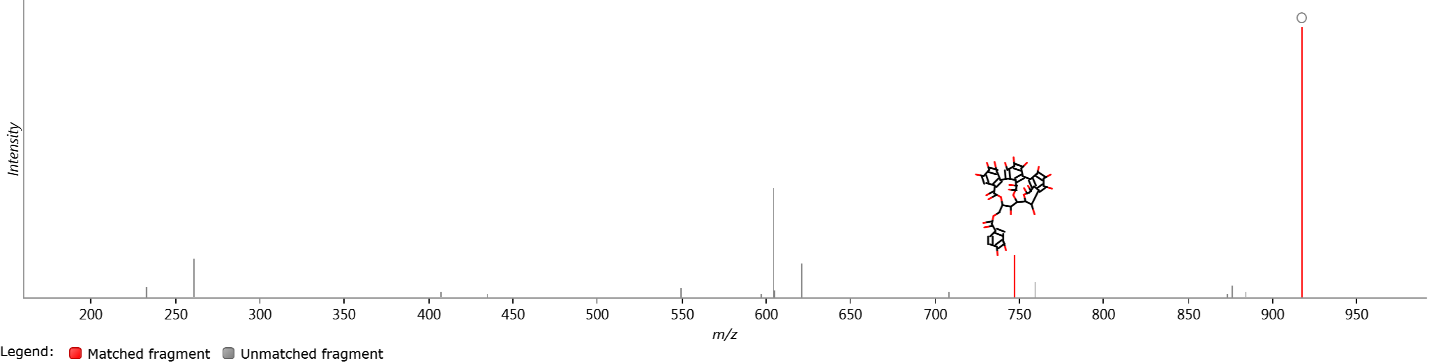


Undefined Ellagitannin-3


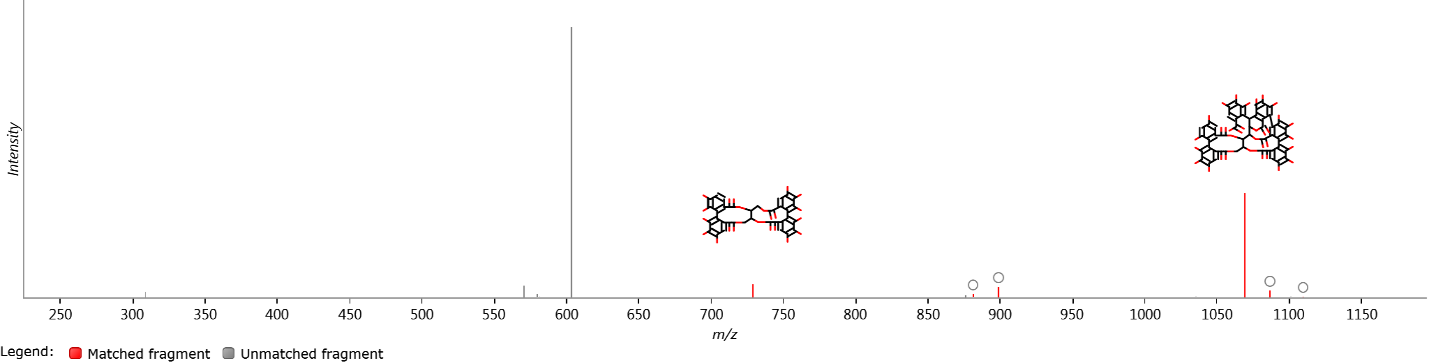

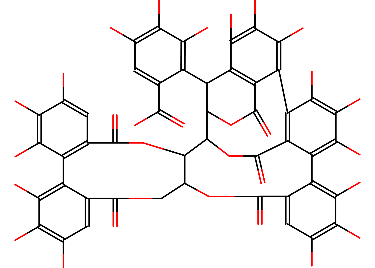


## Figure S16 Penta-hydroxy-flavone


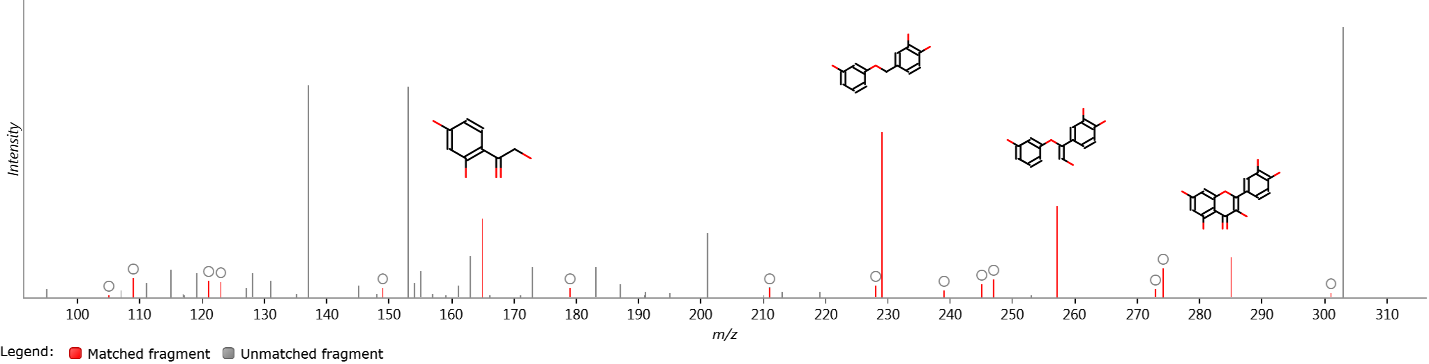

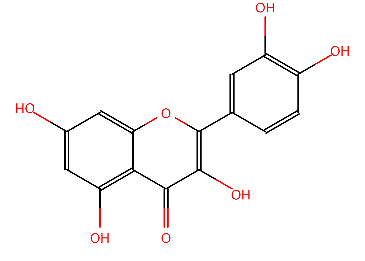


##
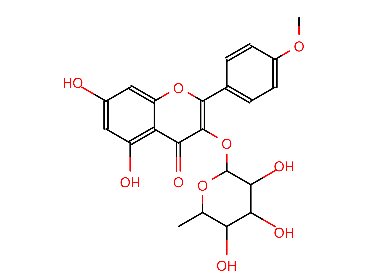

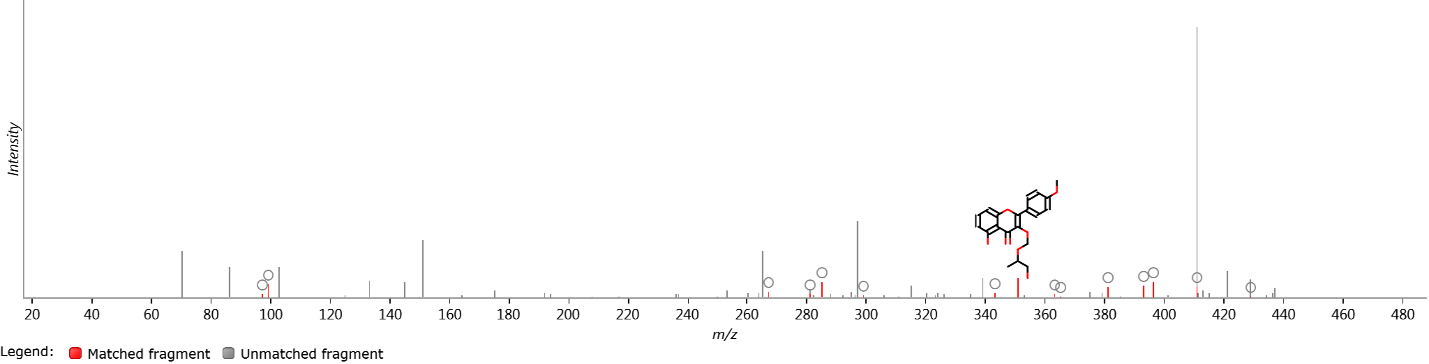
Figure S17 Kaempferide-3-rhamnoside

## Figure S18 Kaempferol


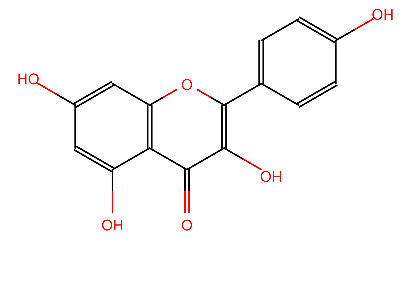

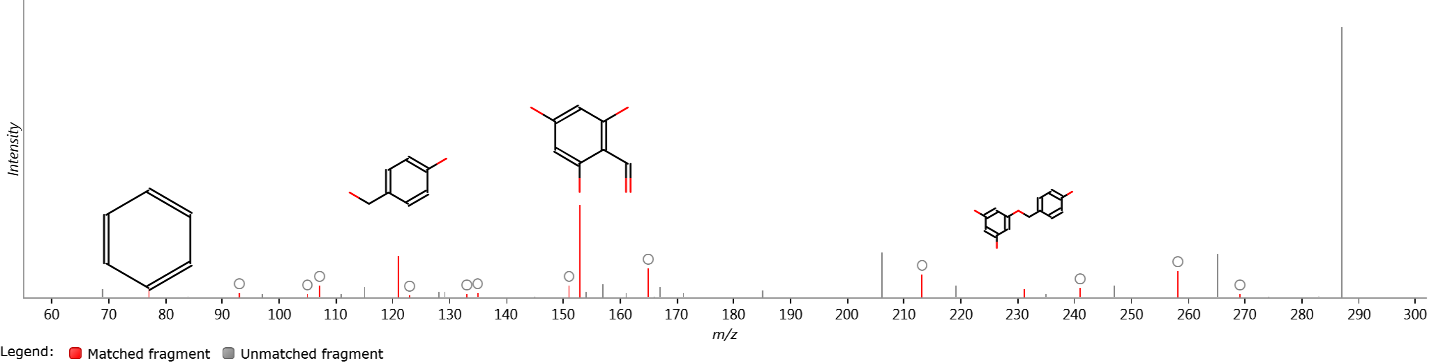


## Figure S19 Pentahydroxy-flavanonol


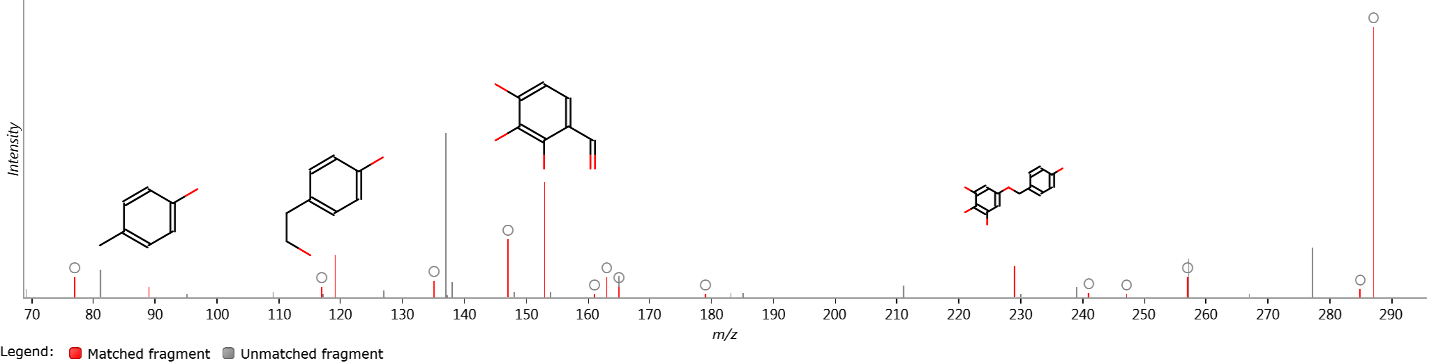

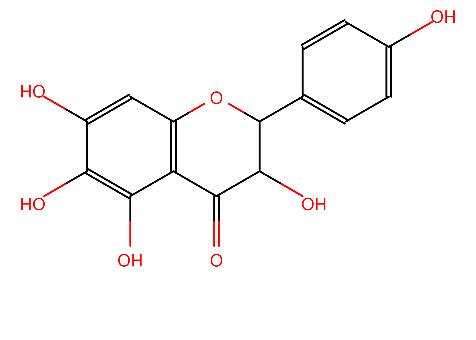


## Figure S20 Quercetin


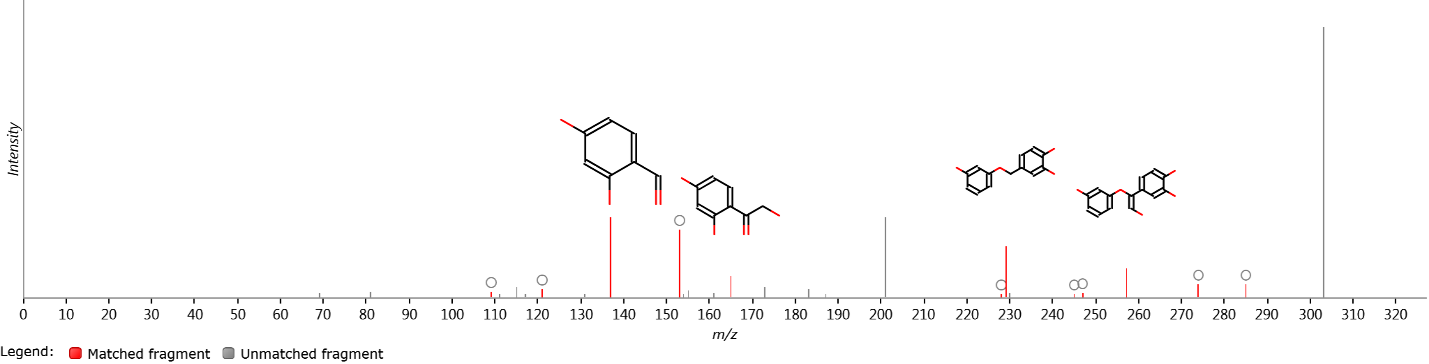

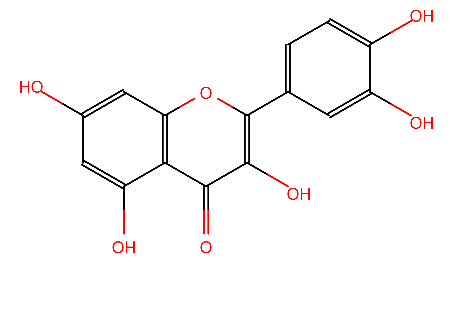


##
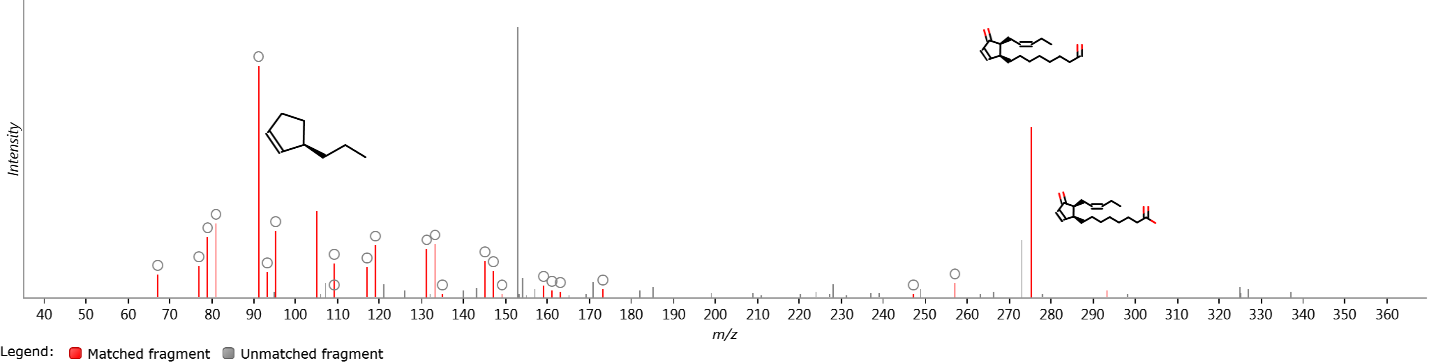
Figure S21 12-Oxophytodienoic acid


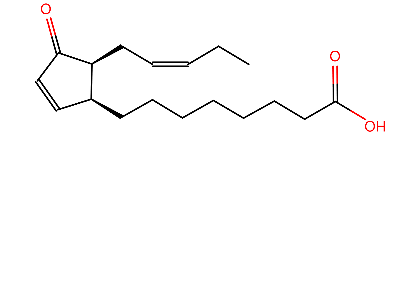


## Figure S22 Trihydroxy-dimethoxyflavone (3,7 dimethylquercetin)


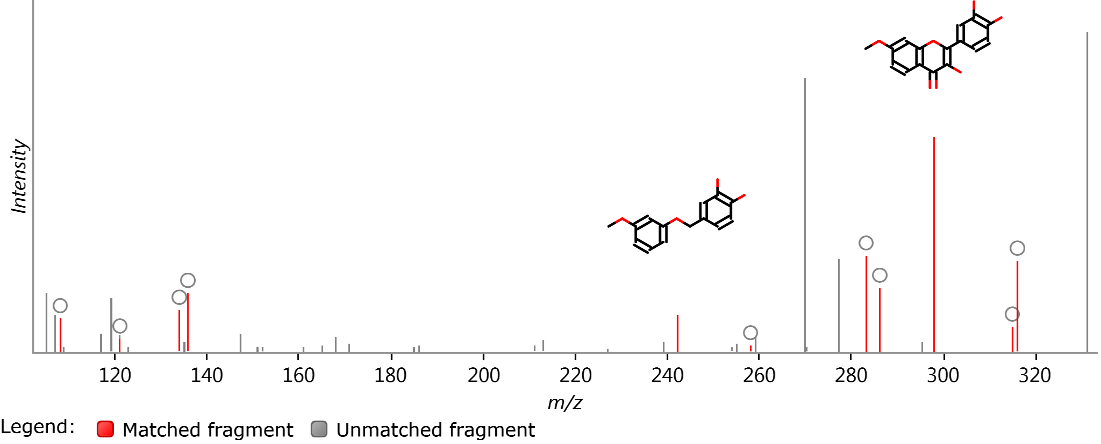

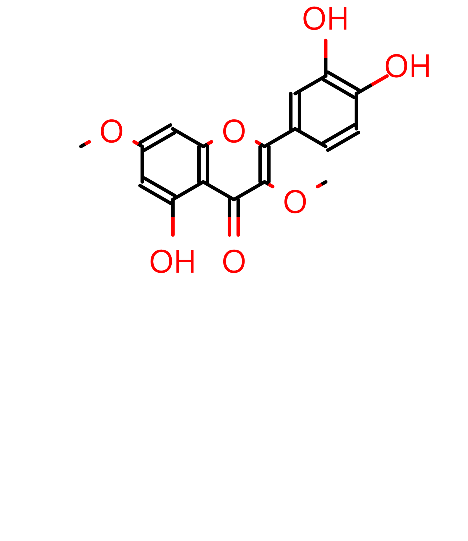


## Figure S23 Trihydoxy-trimethoxy-flavone (dihydroxy-2-(hydroxy-methoxyphenyl)-dimethoxy-4H-chromen-4-one)


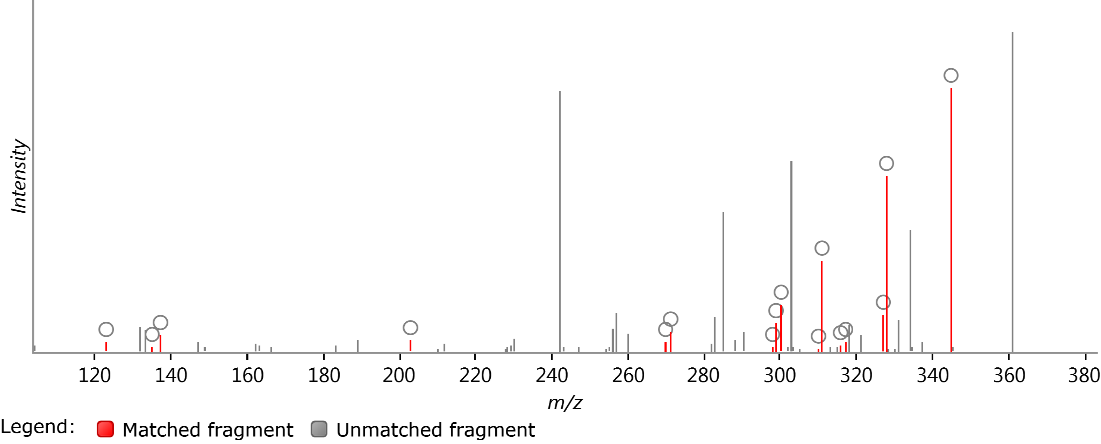

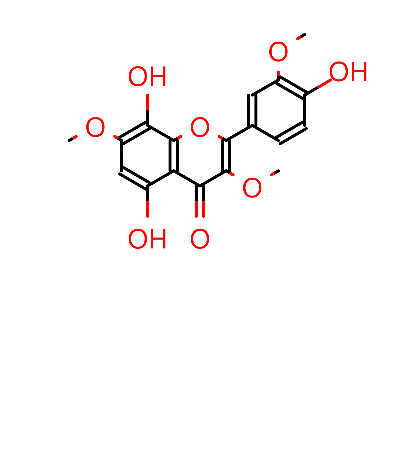


## Figure S24 (E,E)-Piperlonguminine


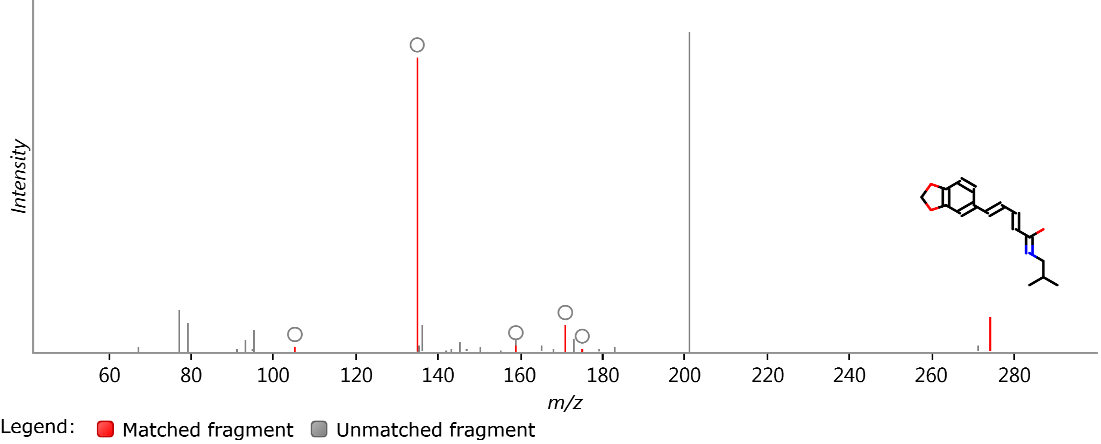

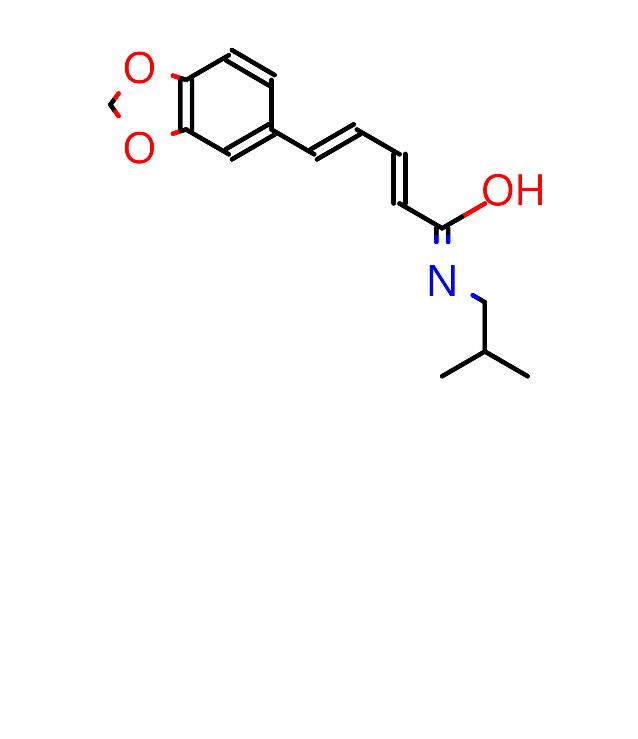


## Figure S25 3-Oxo-12,18-ursadien-28-oic acid


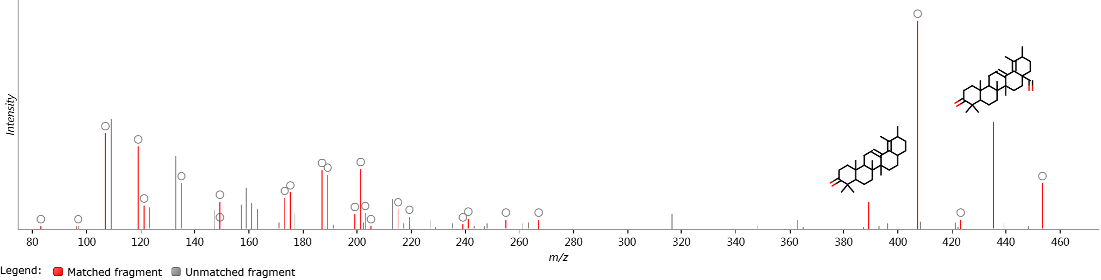

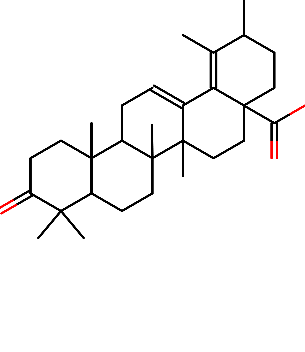


## Figure S26 Piperine


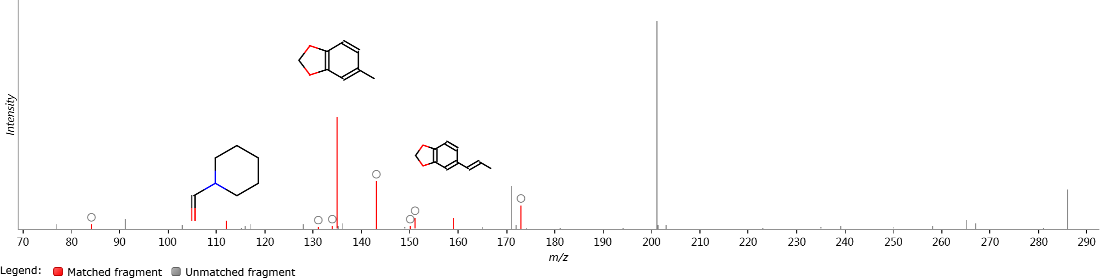

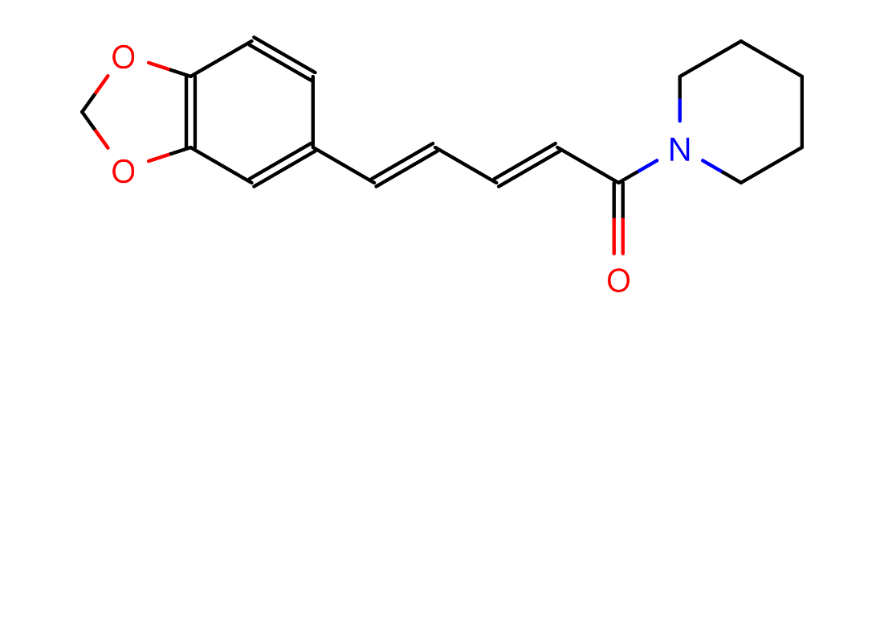


## Figure S27 LysoPC (18:3(6Z,9Z,12Z))


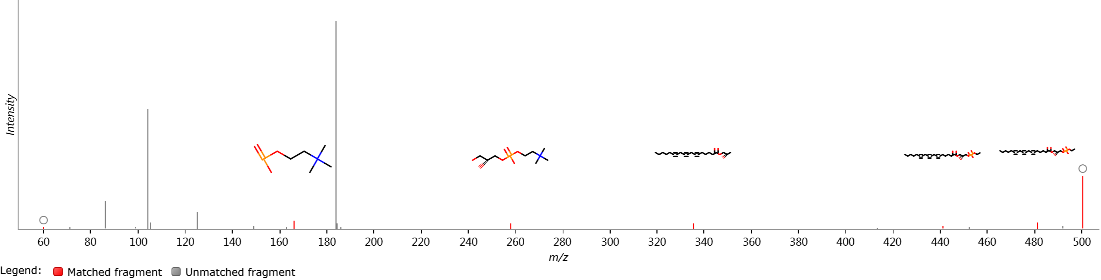

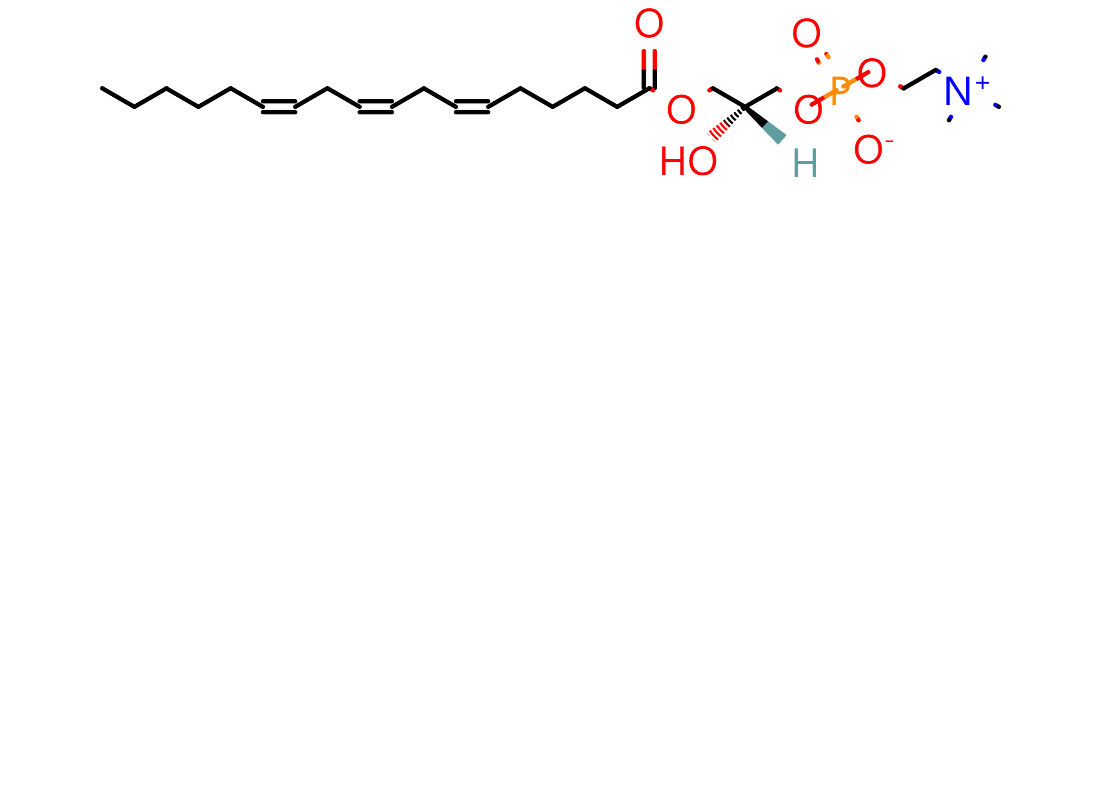


## Figure S28 7-Hydroxy-3,4',8-trimethoxyflavone


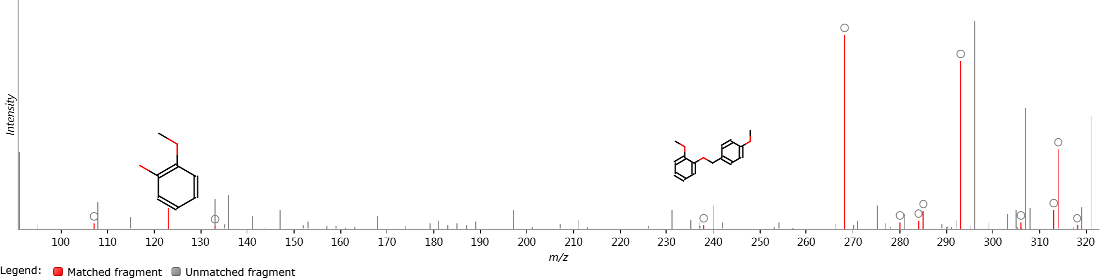

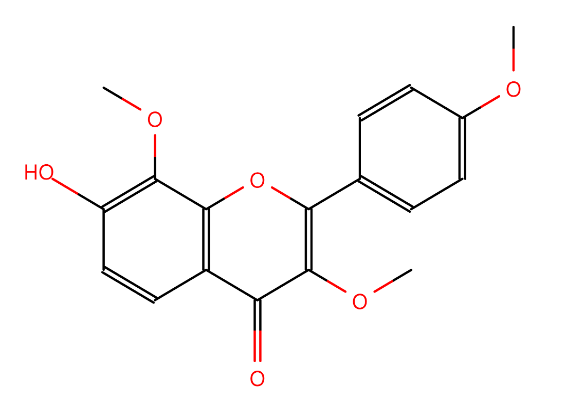


## Figure S29 (+)-(5S,10R)-10,12-dihydroxy-7-oxo-20-norabieta-8,11,13-triene


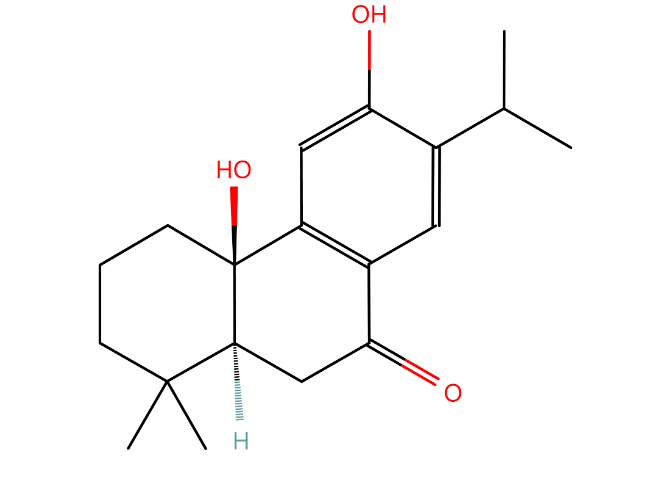

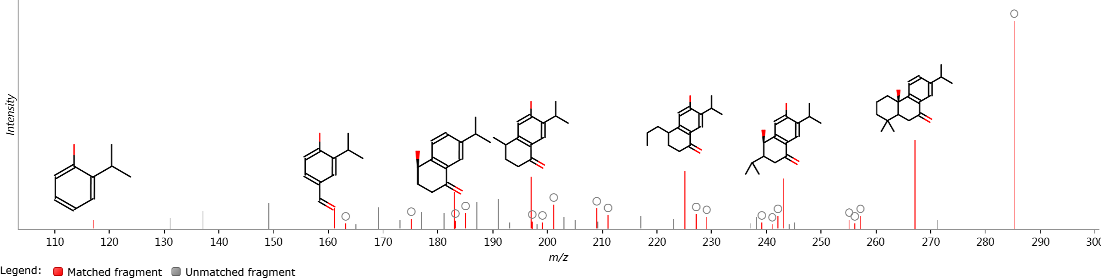


## Figure S30 alpha-Irone


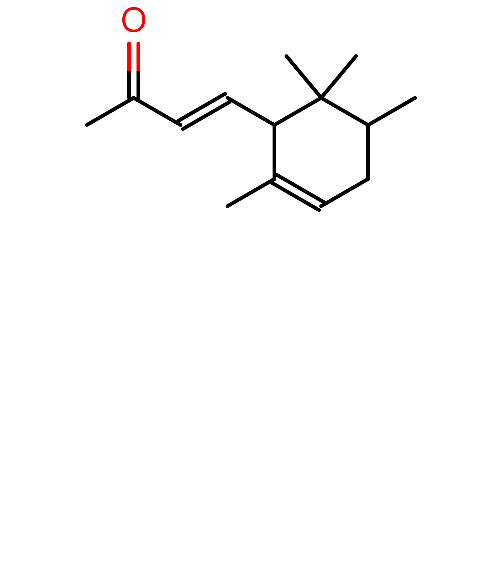

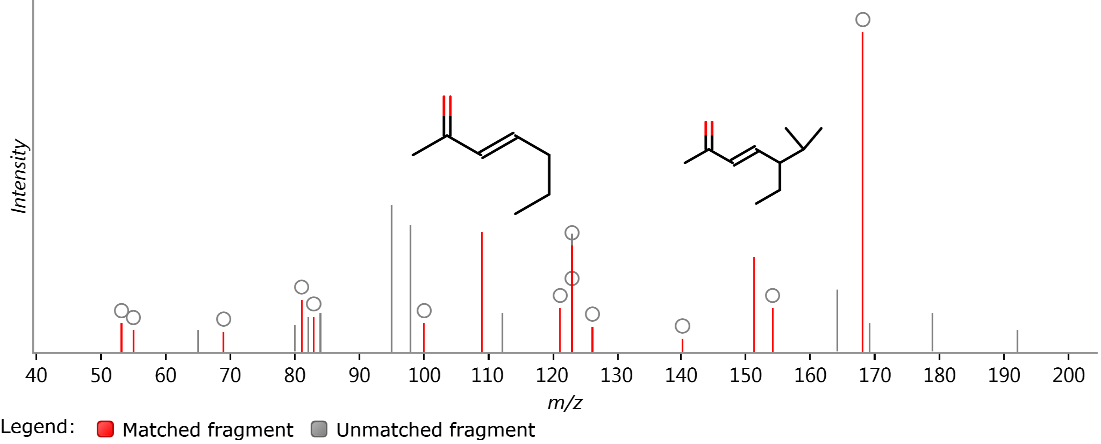


## Figure S31 LysoPC(16:0/0:0)


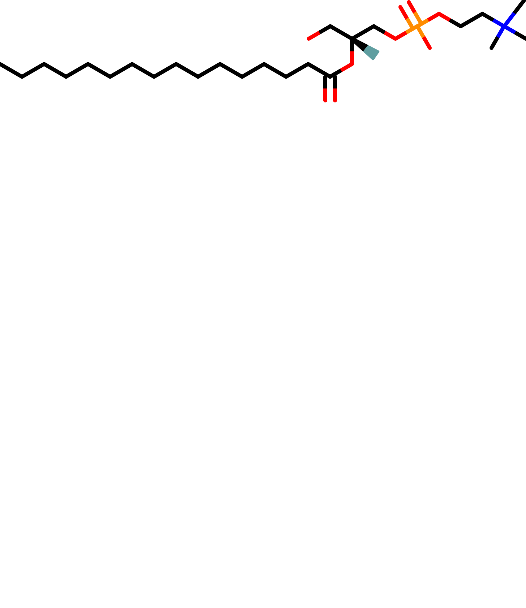

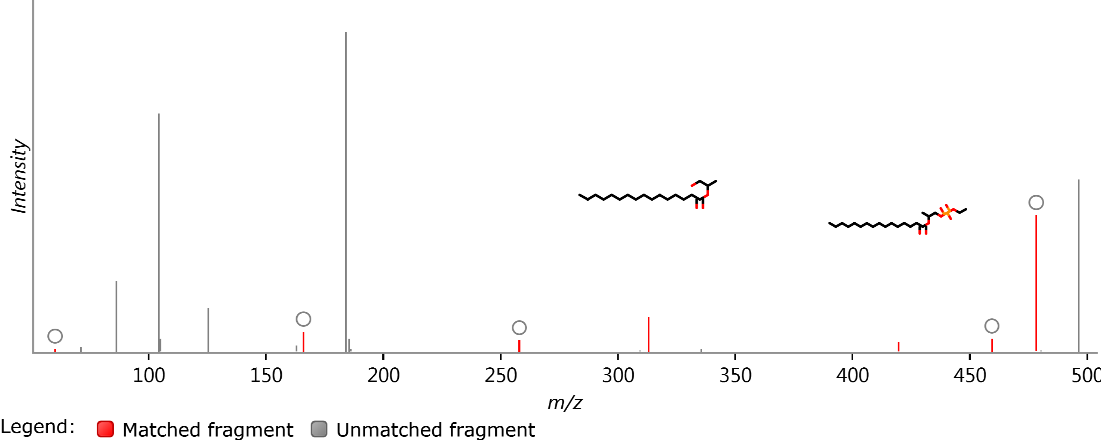


##
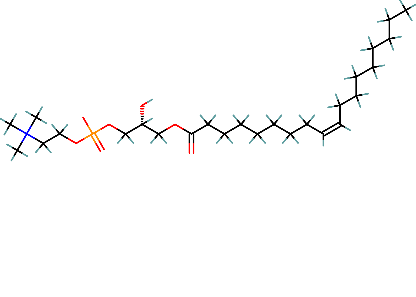
Figure S32 LPC 18:1


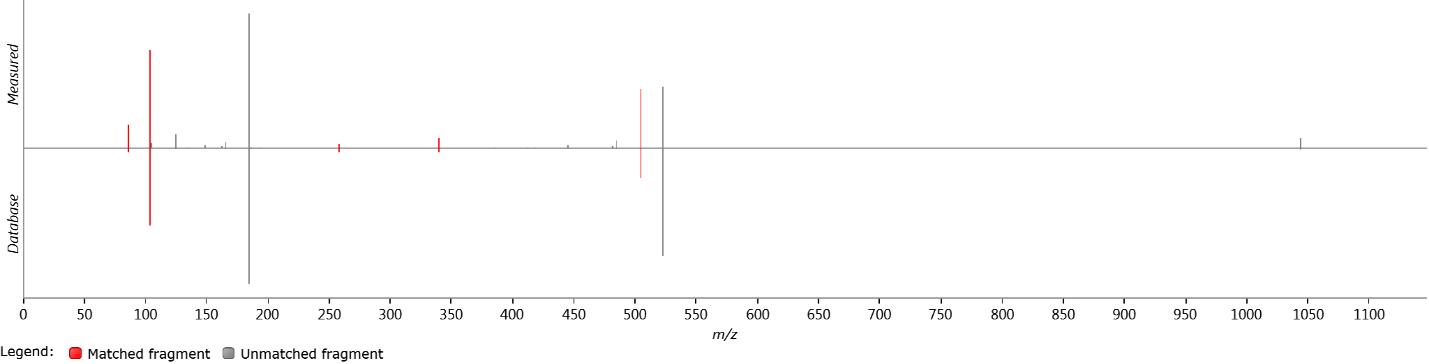


## Figure S33 Undefined Triterpene


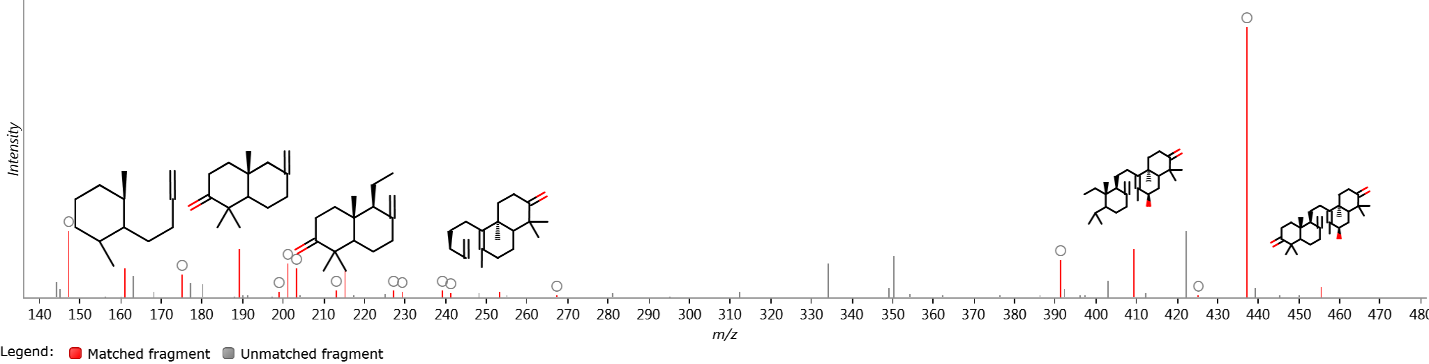

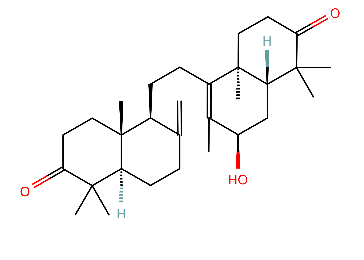


A


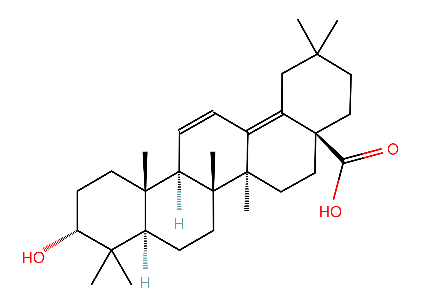

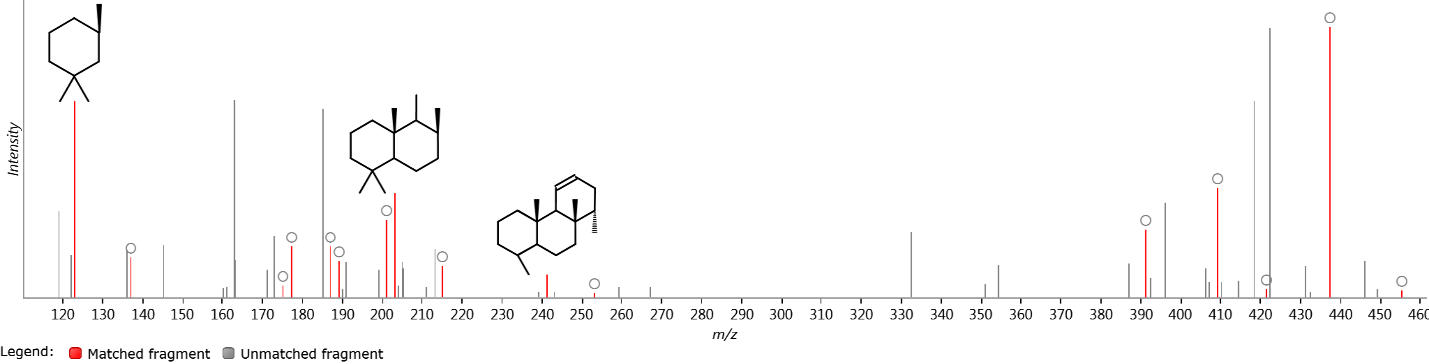


B


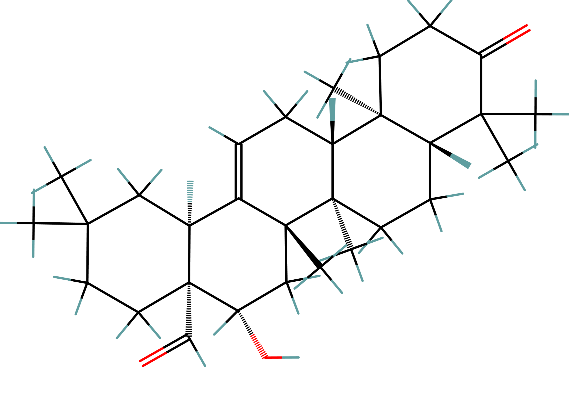

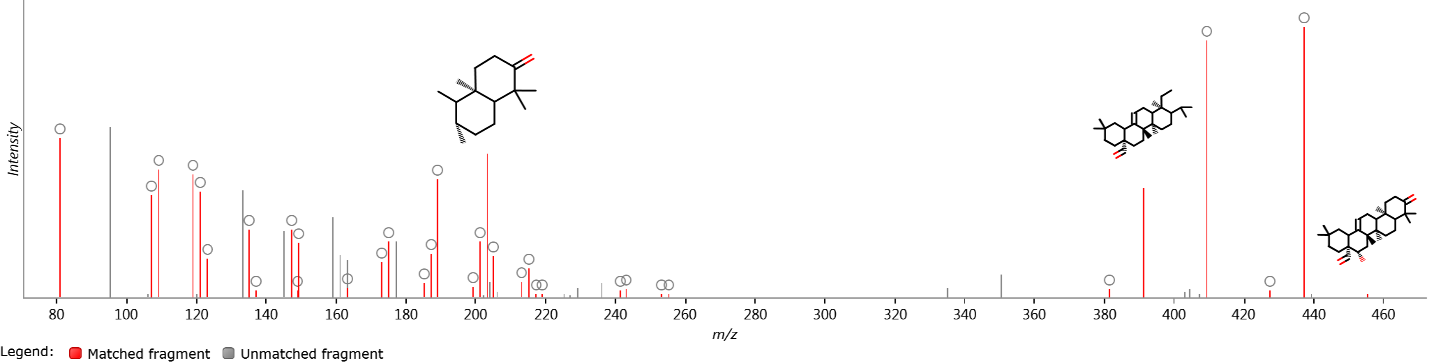


C

## Figure S34 2-Deoxycastasterone


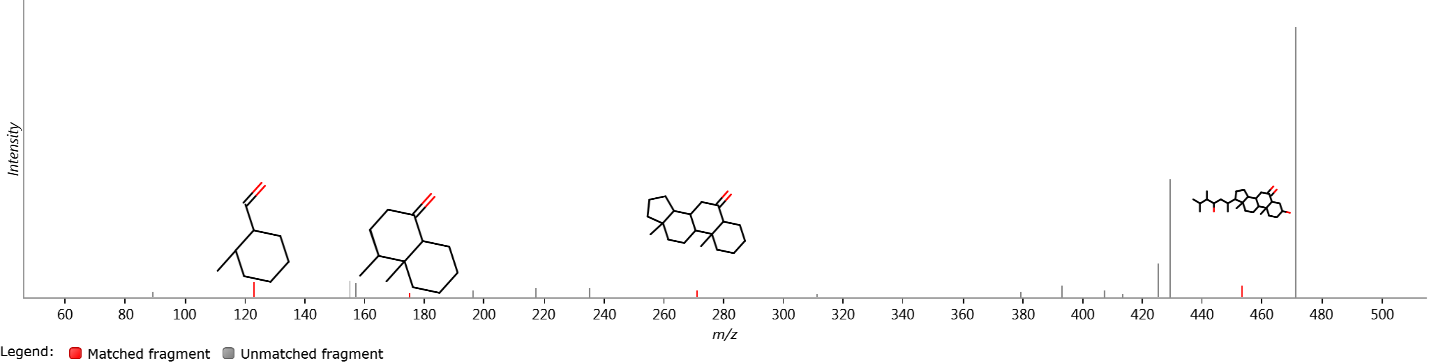

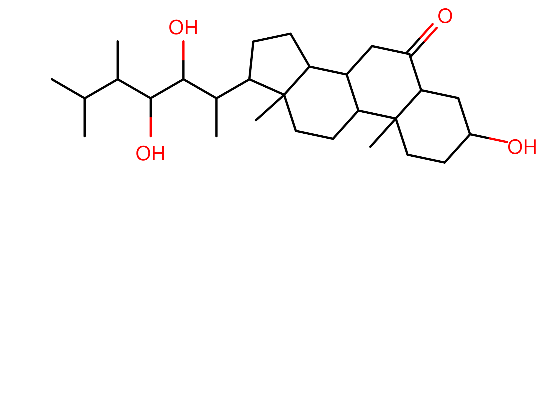

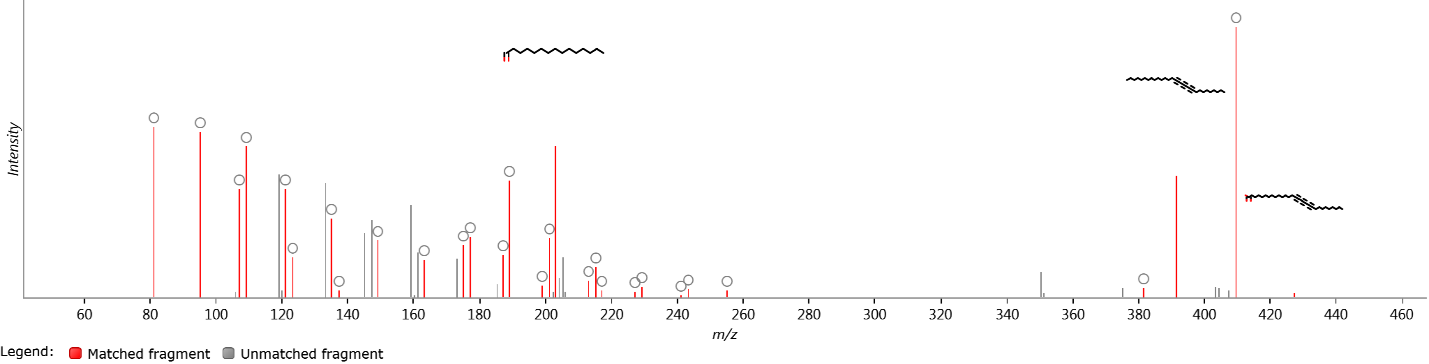

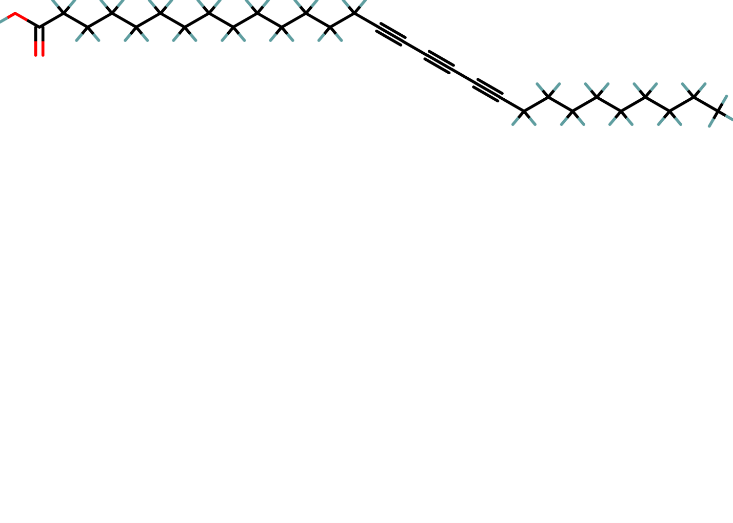


## Figure S35 15,17,19-Nonacosatriynoic acid

## Figure S36 4α-formyl-4β-methyl-5α-cholesta-8,24-dien-3β-ol


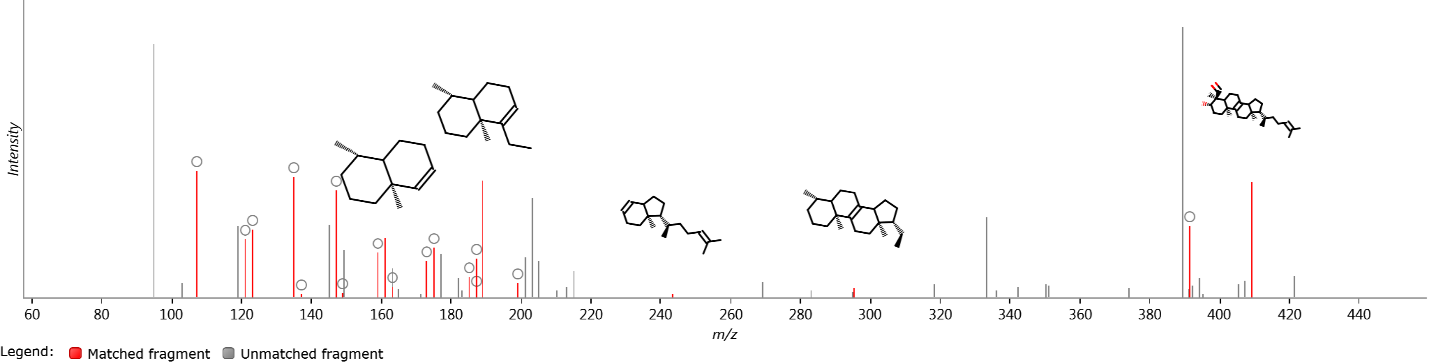

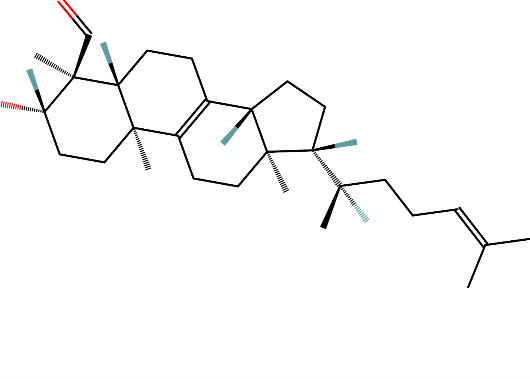


## Figure S37 LPC 18:0


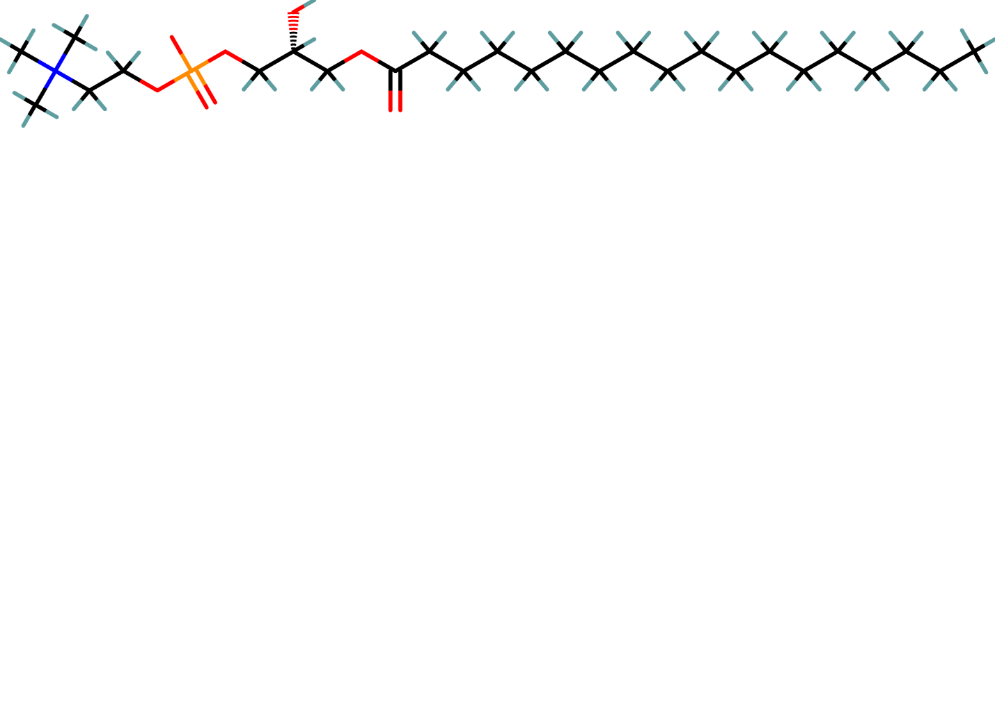

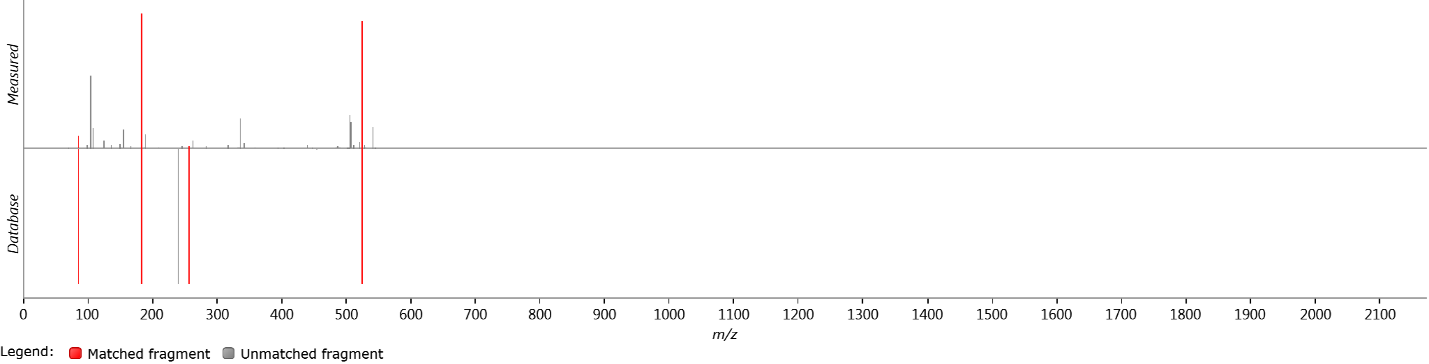


## Figure S38 MG(0:0/18:3(9Z,12Z,15Z)/0:0)


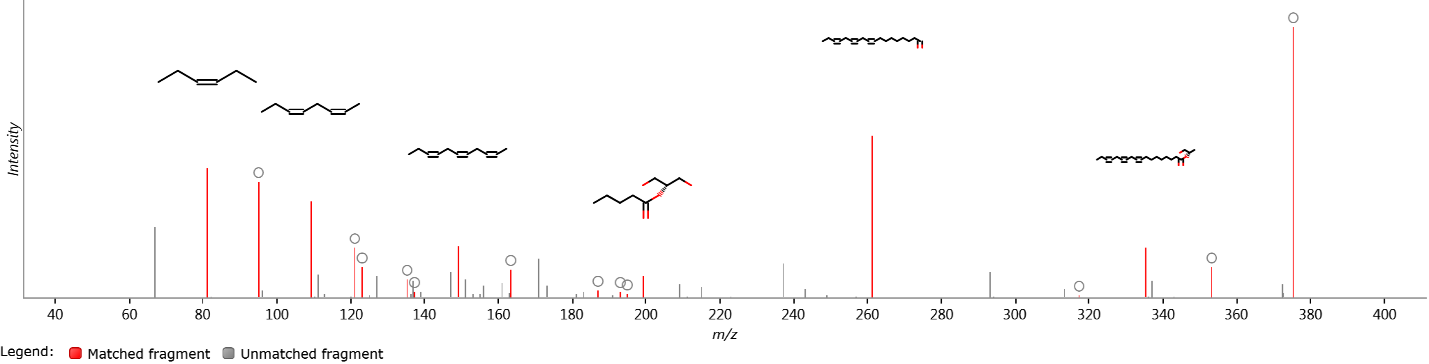

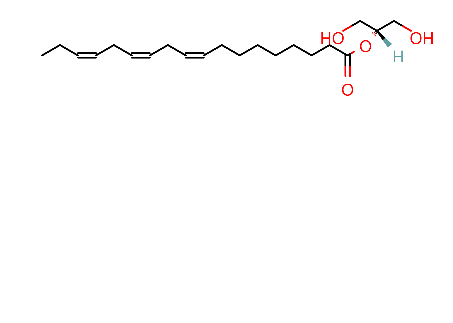


## Figure S39 (2β,3β,5β,9ξ,22R)-2,3,14,20,22,26-Hexahydroxycholest-7-en-6-one


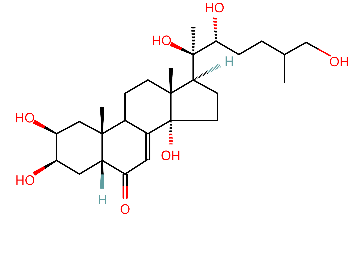

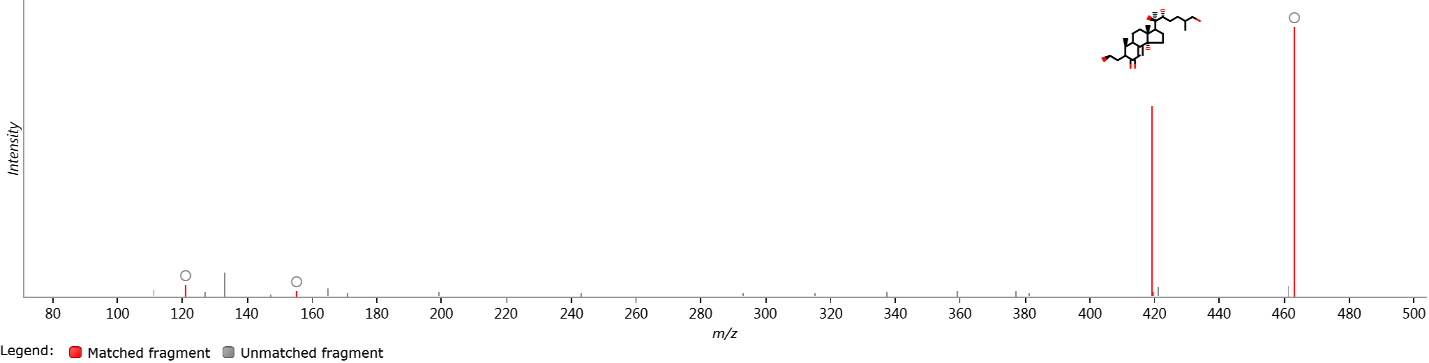


## Figure S40 5-Alpha-Pregnane-3-Beta-Ol-Hemisuccinate


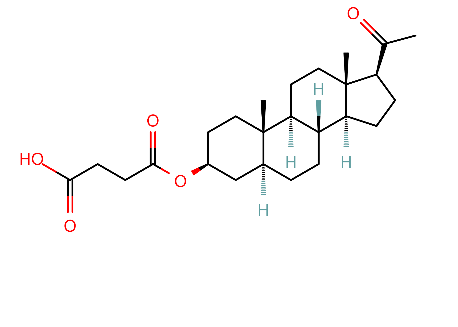

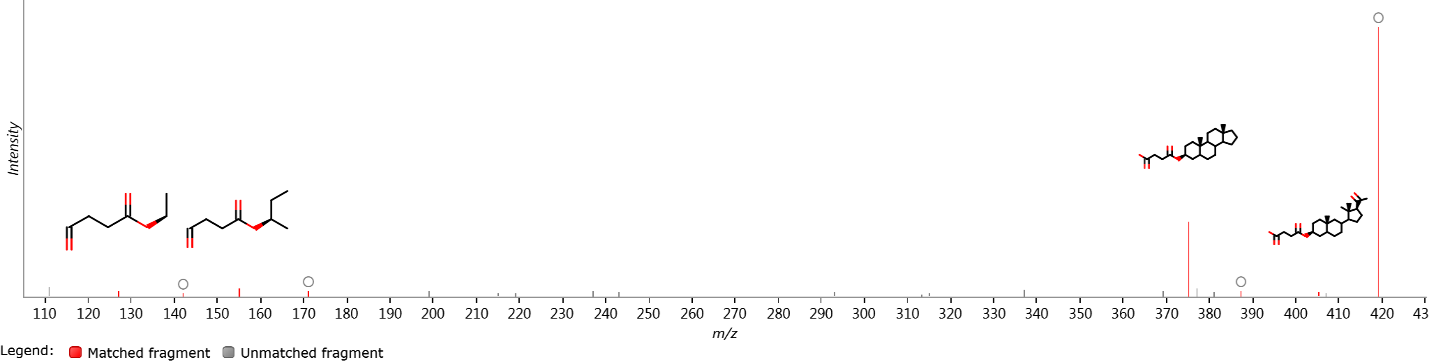


## Figure S41 (2E,4E,12E)-13-(1,3-benzodioxol-5-yl)-N-(2-methylpropyl)trideca-2,4,12-trienamide


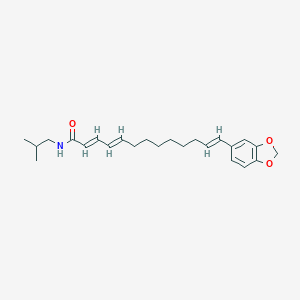

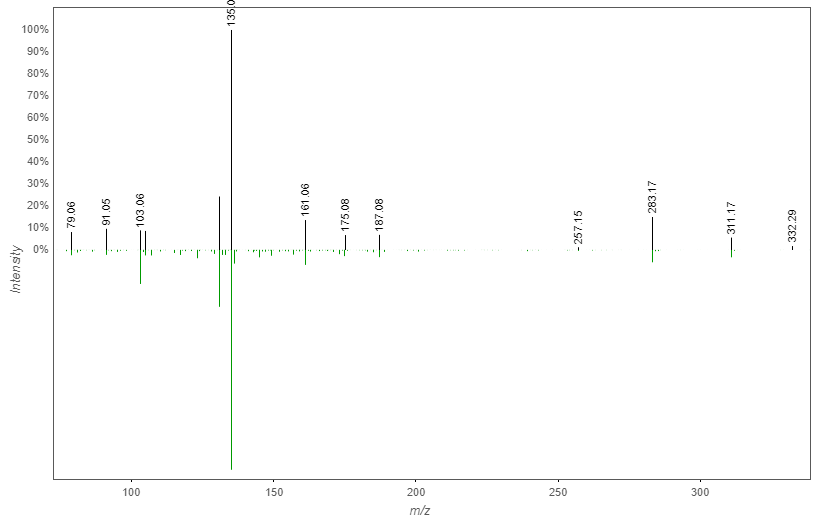


## Figure S42 Dihydroxy-dimethoxyflavone (Crisimartin)


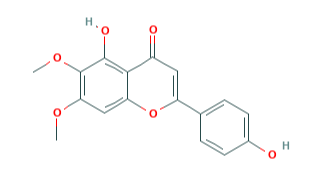

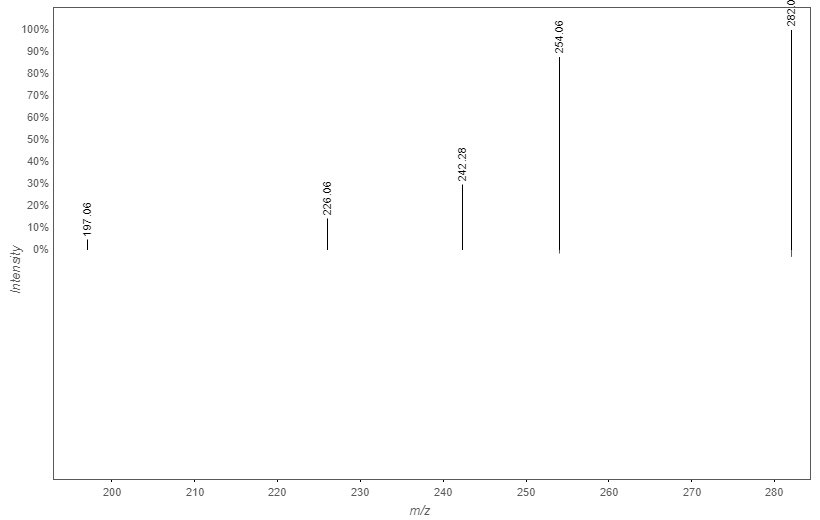


## Figure S43 13S-Hydroxy-9Z,11E,15Z-octadecatrienoic acid


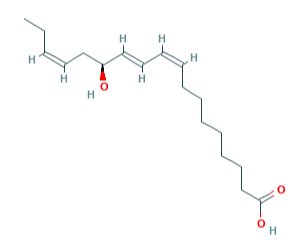

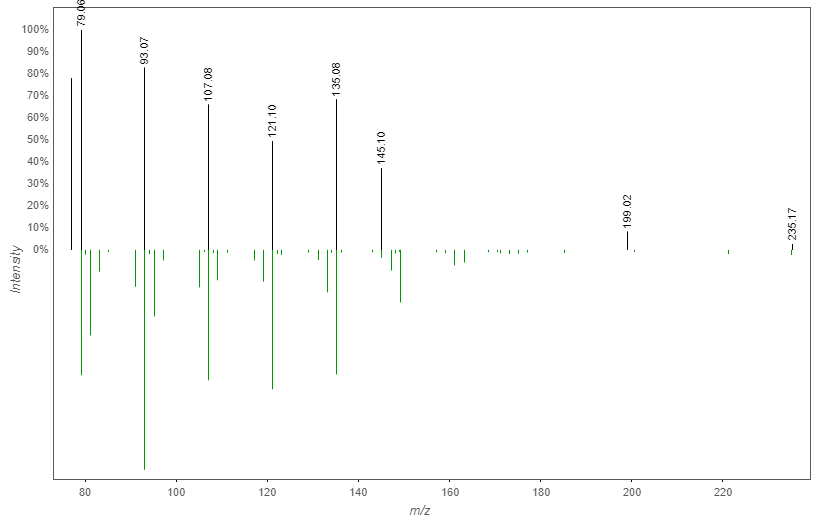

Supplement: Supplementary file 1 — Additional file 1: Table S1. Metabolomic profiling of Actaea racemosa using UPLC–MSn, List of the identified metabolites in negative mode; structures, MSn ions and/or fragmentation tree (Figures S1–S12) and the identified metabolites in positive mode; structures, MSn ions and/or fragmentation tree (Figures S13–S43). [file 13020_2021_444_MOESM1_ESM.docx]
